# Concurrently Boosting Activity and Stability of Oxygen Reduction Reaction Catalysts via Judiciously Crafting Fe–Mn Dual Atoms for Fuel Cells

**DOI:** 10.1007/s40820-024-01580-5

**Published:** 2024-12-16

**Authors:** Lei Zhang, Yuchen Dong, Lubing Li, Yuchuan Shi, Yan Zhang, Liting Wei, Chung-Li Dong, Zhiqun Lin, Jinzhan Su

**Affiliations:** 1 International Research Center for Renewable Energy, State Key Laboratory of Multiphase Flow in Power Engineering, Xi’an Jiaotong University, Xi’an, 710049 People’s Republic of China; 2 Department of Physics, Tamkang University, New Taipei City, Taiwan, 25137 People’s Republic of China; 3 Department of Chemical and Biomolecular Engineering, National University of Singapore, Engineering Drive 4, Singapore, 117585 Singapore

**Keywords:** Doping-adsorption-pyrolysis, Dual-atom catalysts, Oxygen reduction reaction, Fuel cells

## Abstract

The ability to unlock the interplay between the activity and stability of oxygen reduction reaction (ORR) represents an important endeavor toward creating robust ORR catalysts for efficient fuel cells. Herein, we report an effective strategy to concurrent enhance the activity and stability of ORR catalysts via constructing atomically dispersed Fe–Mn dual-metal sites on N-doped carbon (denoted (FeMn-DA)–N–C) for both anion-exchange membrane fuel cells (AEMFC) and proton exchange membrane fuel cells (PEMFC). The (FeMn-DA)–N–C catalysts possess ample dual-metal atoms consisting of adjacent Fe-N_4_ and Mn-N_4_ sites on the carbon surface, yielded via a facile doping-adsorption-pyrolysis route. The introduction of Mn carries several advantageous attributes: increasing the number of active sites, effectively anchoring Fe due to effective electron transfer to Mn (revealed by X-ray absorption spectroscopy and density-functional theory (DFT), thus preventing the aggregation of Fe), and effectively circumventing the occurrence of Fenton reaction, thus reducing the consumption of Fe. The (FeMn-DA)–N–C catalysts showcase half-wave potentials of 0.92 and 0.82 V in 0.1 M KOH and 0.1 M HClO_4_, respectively, as well as outstanding stability. As manifested by DFT calculations, the introduction of Mn affects the electronic structure of Fe, down-shifts the *d*-band Fe active center, accelerates the desorption of OH groups, and creates higher limiting potentials. The AEMFC and PEMFC with (FeMn-DA)–N–C as the cathode catalyst display high power densities of 1060 and 746 mW cm^−2^, respectively, underscoring their promising potential for practical applications. Our study highlights the robustness of designing Fe-containing dual-atom ORR catalysts to promote both activity and stability for energy conversion and storage materials and devices.

**Supplementary Information:**

The online version contains supplementary material available at 10.1007/s40820-024-01580-5.

## Highlights

- Fe–Mn dual-atom catalysts exhibit superior oxygen reduction reaction (ORR) activity and stability, with high half-wave potentials in both alkaline and acidic conditions.
- Synergistic Mn incorporation effectively anchors Fe atoms, mitigates the Fenton reaction, and enhances the durability of ORR catalysts.
- Advanced characterization and density-functional theory calculations reveal Mn-induced electronic structure modifications, promoting superior ORR kinetics and active site performance.
- (FeMn-DA)-N-C catalysts show remarkable potential for practical fuel cell applications.

**Supplementary Information:**

The online version contains supplementary material available at 10.1007/s40820-024-01580-5.

## Graphical abstract

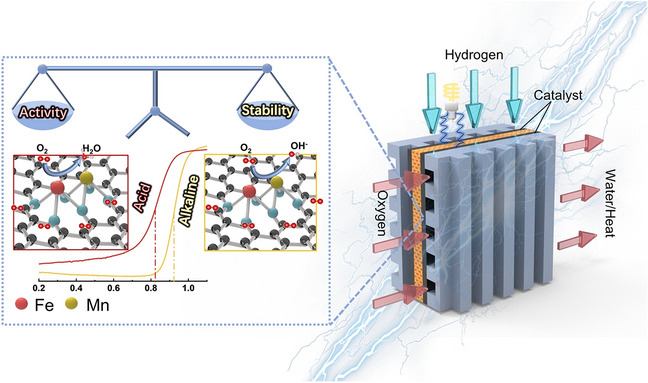

## Introduction

The past several decades witnessed the advances in platinum group metal-free catalysts as the cathode for hydrogen fuel cells [1–4]. Single-atom catalysts (SACs) have garnered much attention due to their excellent activity, selectivity, and maximum atomic utilization [5–7]. Among them, owing to low cost and outstanding oxygen reduction reaction (ORR) activity, iron–nitrogen co-doped carbon (Fe–N–C) SACs (denoted (Fe-SA)–N–C) with highly dispersed Fe-N_x_ are regarded as the most promising, low-cost alternative to platinum-based catalysts [8–10]. However, Fe–N–C catalysts are prone to participate in the Fenton reaction, in which dissolved Fe ions combine with H_2_O_2_ produced by partial two-electron reaction to produce hydroxyl radicals. As such, it leads to carbon oxidation and demetallization, thereby reducing ORR stability [11–14]. In contrast to SACs, dual-atom catalysts (DACs) carry more complex and flexible synergistic active sites [15–17]. Recent studies revealed that the introduction of additional active sites (e.g., Co, Ni, and Mn) into the Fe-N_4_ structure could effectively regulate the electronic structure of the latter and optimize the adsorption free energy of intermediates [2, 12, 18–20]. More importantly, the Fenton reaction between Mn ions and H_2_O_2_ is difficult to occur, thereby conferring high stability to the catalyst [13, 21–23]. Thus, the introduction of Mn sites into the Fe-N_4_ sites may inhibit the Fenton reaction and in turn improve the ORR activity.

The key to render the practical application of non-noble metal ORR catalysts for fuel cells lies in improving the effective active sites and accelerating the transfer of oxygen in electrode. Notably, many ORR catalysts composed of M–N–C (M = Fe, Co, Mn, etc.) structures have low performance in real proton exchange membrane fuel cells (PEMFC) and anion-exchange membrane fuel cells (AEMFC), despite their excellent performance on rotating disk electrodes (RDE) [8, 24, 25]. This is because in fuel cell, the active sites of catalysts distributed on the surface layer contact with reactive gases and ionomers, forming a three-phase interface conducive to the reaction [26–30]. However, the M–N–C ORR catalysts obtained by capitalizing on zeolite imidazolium framework (ZIF) nanocrystals as precursors, followed by high-temperature carbonization have most of the M-N_x_ active sites buried within the carbon matrix, leading to inaccessibility to the ionomer [31]. In contrast, the shallowly distributed catalytic sites can shorten the diffusion distance of O_2_ and H_2_O and promote the formation of the three-phase interface of fuel cell [32–34]. Hemin, which contains a FeN_4_ chelate structure and two carboxylated metal macrocycles, is a natural porphyrin-iron complex with potential for use in the preparation of ORR catalysts [35, 36]. It is larger than the ZIF cage and can be adsorbed on the ZIF surface instead of entering the nanocavity [37]. At the same time, the cage of ZIF is broken by Hemin after pyrolysis at high temperature, thereby releasing the microcavity confinement and forming mesoporous and macroporous structures that are favorable for the substance transport of fuel cell.

Herein, we developed an effective strategy to concurrently optimize the activity and stability of ORR catalysts composed of atomically dispersed Fe–Mn dual-metal sites on N-doped carbon (referred to as (FeMn-DA)–N–C) for AEMFC and PEMFC. Specifically, (FeMn-DA)–N–C catalysts synthesized using a doping-adsorption-pyrolysis approach possess a FeMn-N_6_ structure, derived from the Mn-N_4_ site combined with an adjacent Fe-N_4_, as revealed by X-ray absorption spectroscopy. The density-functional theory (DFT) calculations show that the introduction of Mn atom modulates the electronic structure of the active Fe site and optimizes the adsorption energy of the reaction intermediate and the *d*-band center of the Fe center. The half-wave potentials of (FeMn-DA)–N–C in 0.1 M KOH and 0.1 M HClO_4_ are found to be 0.92 and 0.82 V, respectively. Concurrently, the stability of (FeMn-DA)–N–C outperforms (Fe-SA)–N–C counterpart. Notably, the metal consumption in the (FeMn-DA)–N–C catalysts is greatly reduced compared to that of the (Fe-SA)–N–C catalyst, as corroborated by the inductively coupled plasma-mass spectrometry (ICP-MS) as well as energy dispersive X-ray spectroscopy (EDS) mapping before and after the accelerated degradation tests (ADT). The strong interaction between the neighboring Mn site and Fe site not only increases the activity of catalysts, but also efficiently anchors the Fe atoms. Meanwhile, the addition of Mn active site also profoundly prevents Fenton reaction and reduces the loss of metal sites. Consequently, the ORR activity and stability of the (FeMn-DA)-N–C catalysts are greatly enhanced. Furthermore, the (FeMn-DA)–N–C-based AEMFC and PEMFC manifest high power densities of 1060 and 746 mW cm^−2^, respectively, outpacing (Fe-SA)–N–C-based counterparts, underscoring their promising potential in practical fuel cell applications.

## Experimental Section

### Synthesis of Mn-ZIF-8

2.79 g of Zn (NO_3_)_2_·6H_2_O and 1.116 g Mn (OAc)_2_·4H_2_O were dissolved in 75 mL methanol. 3.08 g 2-MeIM in 75 mL methanol was subsequently injected into the above solution and aged for 24 h at room temperature. The as-obtained precipitates were centrifuged, washed with methanol several times and dried under vacuum at 343 K for 10 h to obtain Mn-ZIF-8@Hemin.

### Synthesis of Mn-ZIF-8@Hemin

0.5 g Mn-ZIF-8 was dissolved in 100 mL DMF. 0.05 g of Hemin in 100 mL DMF was subsequently injected into the above solution and aged for 24 h at room temperature. The as-obtained precipitates were centrifuged, washed with methanol several times and dried under vacuum at 343 K for 10 h to yield Mn-ZIF-8@Hemin.

### Synthesis of (FeMn-DA)–N–C

The as-synthesized Mn-ZIF-8@Hemin was placed in a tube furnace and then heated to 1193 K for 2 h at a heating rate of 275 K min^−1^ under flowing Ar gas and then naturally cooled to room temperature to yield (FeMn-DA)–N–C (other conditions are equal; pyrolysis temperatures of 1173 and 1223 K were chosen as controls to explore the temperature effect). More details can be found in Supporting Information.

## Results and Discussion

### Synthesis and Characterization of (FeMn-DA)–N–C

**Fig. 1:**
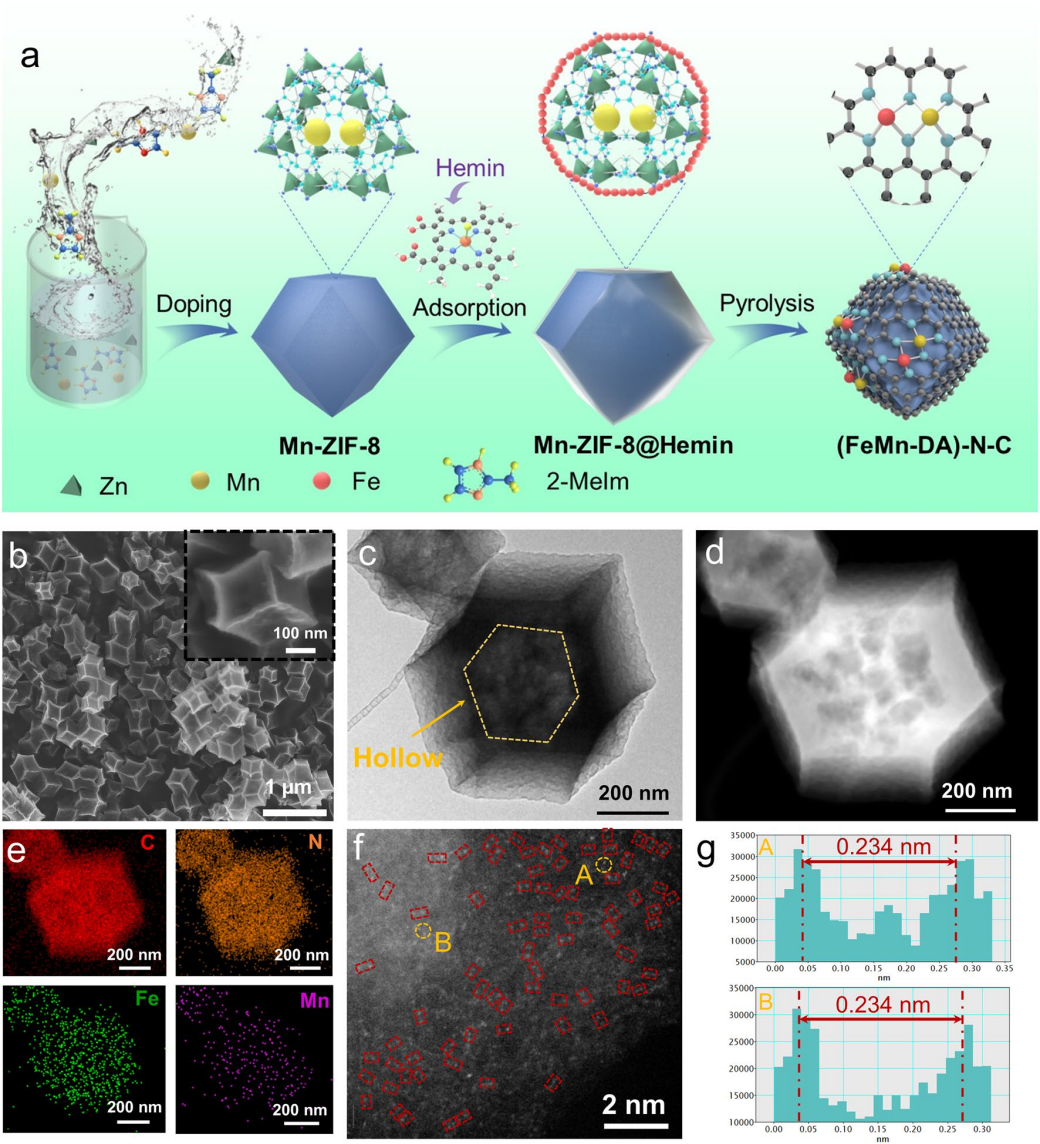
**a** Schematic illustration of the route to (FeMn-DA)–N–C, where the Hemin is represented by red balls in the close-up image of Mn-ZIF-8@Hemin. **b** SEM, **c** TEM, **d** HAADF-STEM, **e** EDS mapping, **f** aberration-corrected HAADF-STEM, and **g** intensity profiles of (FeMn-DA)–N–C

The synthetic route to (FeMn-DA)–N–C is illustrated in Fig. 1a. The guest macromolecule Hemin was introduced to the surface of the host Mn-ZIF-8 to form a Mn-ZIF-8@Hemin structure. The dimension of Hemin is 15.1 × 15.3 × 10.6 Å^3^, while the cavity diameter of the ZIF cage is 11.6 Å. As a result, Hemin adsorbed on the surface of Mn-ZIF-8 instead of entering to the pores. After high-temperature pyrolysis, most of the Fe sites remain on the surface of catalyst, forming more readily available dual-metal atom active sites with the adjacent Mn sites. Moreover, the adsorption of Hemin broke the confinement effect of the microcavity of ZIF, and induced the Kirkendall effect, forming mesoporous and macroporous structures that are favorable for transport of substances in fuel cell. As shown in Fig. S1, the X-ray diffraction (XRD) patterns of the catalysts before high-temperature pyrolysis show that the crystal structures of manganese–nitrogen co-doped carbon (Mn–N–C) SACs (denoted (Mn-SA)–N–C), (Fe-SA)–N–C, and (FeMn-DA)–N–C were matched well with those of ZIF-8, implying that the as-prepared catalyst precursors maintained the original zeolite-like structures [27]. After high-temperature carbonization (Fig. S2), only two broad diffraction peaks were observed at 25° and 44°, corresponding to the (002) and (101) faces of graphitic carbon, respectively, and no metal crystalline phases or clusters were detected, confirming that the metal exists in the form of atoms within the carbon skeleton [38]. Scanning electron microscopy (SEM) and transmission electron microscopy (TEM) studies revealed that after high-temperature pyrolysis, (FeMn-DA)–N–C retained the original rhombic dodecahedral structure, and only the surface underwent a distortion, resulting in an increase in the specific surface area and presenting a three-dimensional hierarchical porous structure with a rough surface (Figs. 1b, c, and S3-S7). Moreover, due to the rapid diffusion rate of Zn^2+^ and the slow diffusion rate of Fe^3+^ during pyrolysis, Kirkendall effect was induced [39, 40], leading to the formation of a hollow structure inside, which reduces the resistance to mass transfer and facilitates the transfer of electrons. These features are conducive to the performance of catalysts in fuel cells [39–41]. We used FeCl_3_·6H_2_O with the same molar quantity as Hemin, as a small molecule iron source to conduct control experiment to verify the advantage of Hemin. TEM images show that the large molecule Hemin as the iron source displayed obvious hollow structure, While the small molecule FeCl_3_·6H_2_O as the iron source had no obvious hollow structure (Fig. S5). High-angle annular dark-field scanning transmission electron microscopy (HAADF-STEM) and the corresponding EDS mapping show that C, N, Fe, and Mn were uniformly distributed in (FeMn-DA)–N–C, and no metal particles were observed, suggesting a high degree of dispersion of the metals (Fig. 1d, e). In addition, the TEM and EDS mapping of (Fe-SA)–N–C and (FeMn-DA)–N–C before and after ADT cycling in acidic and alkaline electrolytes were also tested (Figs. S8-S12). Clearly, the Fe content of (Fe-SA)–N–C became significantly less after the ADT test, while the Fe and Mn contents in (FeMn-DA)–N–C did not change significantly, confirming the stability of the (FeMn-DA)–N–C structure. Meanwhile, the Fe and Mn contents of (FeMn-DA)–N–C were determined by ICP-MS, which are 3.27% and 1.48%, respectively. The changes of Fe and Mn metal contents before and after ADT tests in 0.1 M KOH and 0.1 M HClO_4_, respectively, are shown in Table S1. The results indicate that the amount of metal changes in (FeMn-DA)–N–C is smaller than that in (Fe-SA)–N–C. The aberration-corrected HADDF-STEM image shows that a large number of bimetallic single-atom pairs (labeled with red boxes) were uniformly dispersed on the nitrogen-doped carbon nanostructures, and the distances between the metal pairs in the two regions of A and B are approximately 0.234 nm (Fig. 1f, g). Raman spectra show that the as-prepared catalysts had d and g bands at 1344 and 1592 cm^−1^ (Fig. S13), representing carbon lattice defects and *sp*^2^ hybridized graphitic carbon atoms, respectively. Among them, the I_D_/I_G_ value of the (Mn-SA)–N–C catalyst is 1.19, while the *I*_D_/*I*_G_ values of the (Fe-SA)–N–C and (FeMn-DA)–N–C catalysts are 1.29 and 1.31, respectively (the higher the ratio, the higher the degree of defects). From Fig. S6, the surface of (Mn-SA)–N–C is relatively smooth and undistorted, whereas the surface becomes rough and undergoes surface distortion after the Fe addition (Figs. S7 and 1b). This accounts for the higher degree of defects in (Fe-SA)–N–C and (FeMn-DA)–N–C, and the formation of defects facilitates the ORR [42].

**Fig. 2:**
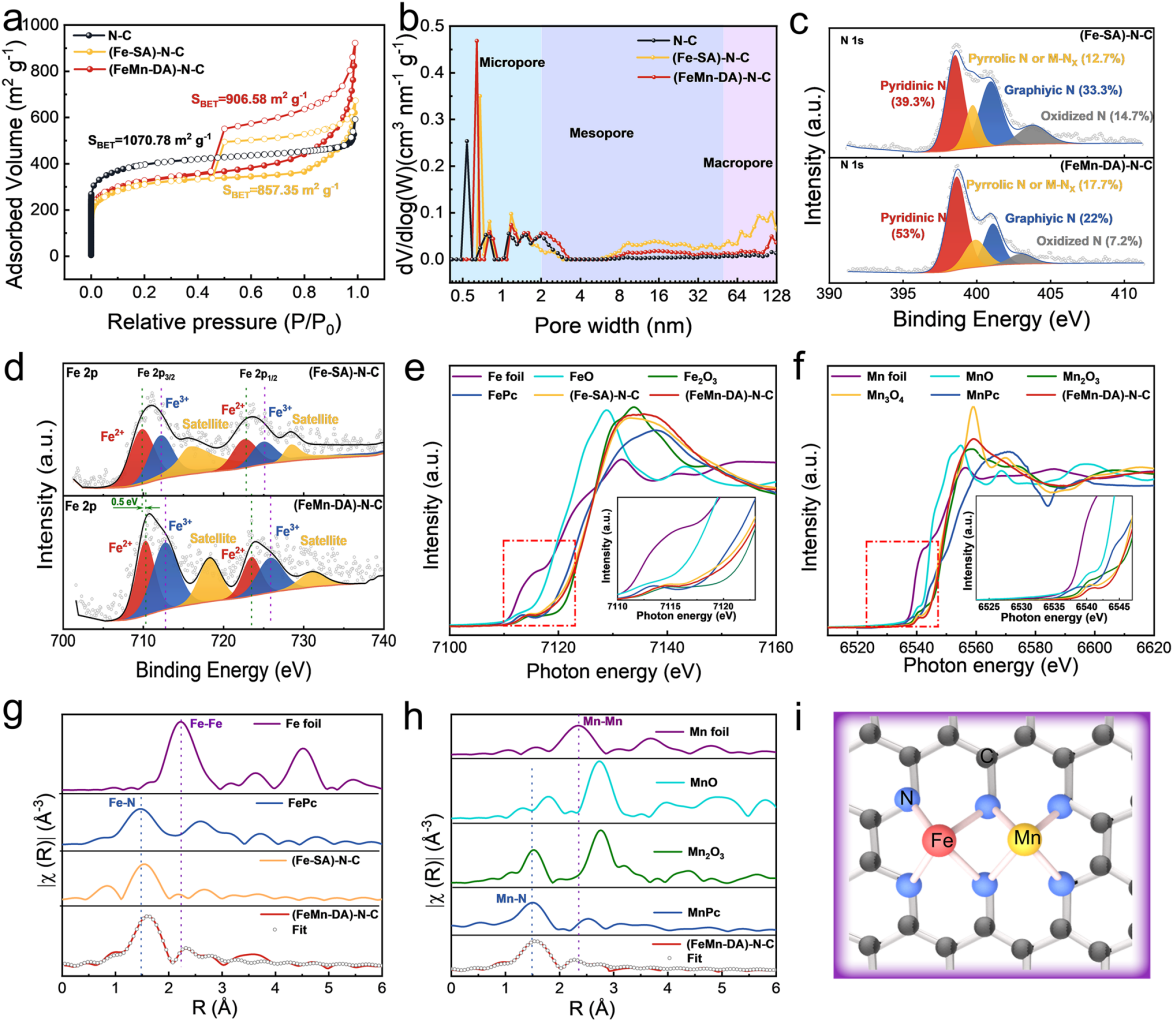
**a** N_2_ adsorption–desorption isotherm. **b** Pore size distributions. **c** High-resolution N 1*s* and comparison of different types of nitrogen content. **d** High-resolution Fe 2*p*. **e, f** XANES spectra of the Fe K-edge and Mn K-edge. **g, h** Fourier-transform EXAFS spectra and fitting curves of (FeMn-DA)–N–C. **i** Structure of (FeMn-DA)–N–C

The N_2_ adsorption/desorption isotherm analysis is shown in Fig. 2a, where N–C exhibited a type I isotherm, indicating that microporous structure predominates in the sample [43]. In contrast, (Fe-SA)–N–C and (FeMn-DA)–N–C showed significant hysteresis loops at high relative pressures (P/P_0_ = 0.45–1.0), suggesting type IV isotherms that confirm mesopore formation [44]. The Brunauer–Emmett–Teller (BET) specific surface area of N–C (1070.78 m^2^ g^−1^) was significantly larger than those of (Fe-SA)–N–C and (FeMn-DA)–N–C (857.35 and 906.58 m^2^ g^−1^, respectively), substantiating the transformation of micropores to mesopores. The non-local density-functional theory (NLDFT) fitting curves show that the N–C samples have only microporous structures (< 2 nm), whereas (Fe-SA)–N–C and (FeMn-DA)–N–C contain micropores, mesopores, and macropores (Fig. 2b). The formation of mesopores and macropores is due primarily to the addition of the macromolecule Hemin which breaks the microcavity confinement effect of the ZIF cage as well as the Kirkendall effect [37, 41]. Specifically, the molecular size of Hemin (Fig. S14, 15.1 × 15.3 × 10.6 Å^3^) is larger than that of ZIF cage (lumen diameter, 11.6 Å), leading to the adsorption of Hemin on the surface of ZIF to form the Mn-ZIF-8@Hemin structure. During pyrolysis, some Hemin breaks the microcavity confinement effect of ZIF cage, forming mesopores. Meanwhile, the aggregation of Hemin molecules results in the Kirkendall effect that continues to induce the formation of mesopores or macropores. We further compared the differences in chemical composition between (Fe-SA)–N–C and (FeMn-DA)–N–C by X-ray photoelectron spectroscopy (XPS). Figure S15 shows the comparison of the C 1*s* peaks between the (Fe-SA)–N–C and (FeMn-DA)–N–C, and the results show that the structure of the C shows no change. Figure 2c compares different types of nitrogen contents between (Fe-SA)–N–C and (FeMn-DA)–N–C. In particular, the content of pyrrolic N or M-Nx (M = Fe, Mn) in (FeMn-DA)–N–C (17.7%) is higher than that in (Fe-SA)–N–C (12.7%), confirming the presence of more M-Nx active sites. In contrast, (FeMn-DA)–N–C contains more pyridinic N than (Fe-SA)–N–C, which is widely recognized as the catalytic active center [45–47]. The Fe 2*p* XPS spectra show a positive peak shift for the 2*p*_1/2_ peak and 2*p*_3/2_ peak of Fe^2+^ and Fe^3+^ in (FeMn-DA)–N–C compared to that of (Fe-SA)–N–C (Fig. 2d), suggesting that Mn altered the electronic structure of Fe, thereby resulting in more electron loss and higher valence state of the Fe atoms. The Mn 2*p* XPS spectra show a negative peak shift for the 2*p*_1/2_ peak and 2*p*_3/2_ peak of Mn^2+^ and Mn^3+^ in (FeMn-DA)–N–C compared to that of (Mn-SA)–N–C, suggesting that Fe causes Mn to acquire more electrons and thus exists in a lower valence state (Fig. S16).

The local structure and coordination environments of Fe and Mn were analyzed via X-ray absorption near edge structure (XANES) and K-edge extended X-ray absorption fine structure (EXAFS) measurements. Figure 2e shows the K-edge XANES spectra of Fe. The positions of the absorption edges of (Fe-SA)–N–C and (FeMn-DA)–N–C are located between FeO and Fe_2_O_3_, confirming that the Fe valence in the catalyst is between divalent and trivalent, in good agreement with the results of the XPS analysis. Figure 2f displays the K-edge XANES spectrum of Mn, and the position of the absorption edge of (FeMn-DA)–N–C was very close to that of Mn_3_O_4_, substantiating the presence of both divalent and trivalent manganese in the catalyst. As shown in the Fourier-transform (FT) k^3^-weighted EXAFS spectrum of Fe K-edge in Fig. 2g, (Fe-SA)–N–C and (FeMn-DA)–N–C show distinct peak at around 1.5 Å corresponding to Fe–N coordination. The fact that (FeMn-DA)–N–C has a peak at 2.32 Å, yet is absent in (Fe-SA)–N–C suggests an interaction between Mn and Fe. Furthermore, as shown in the wavelet transform (WT) plot (Fig. S17), the K value of (FeMn-DA)–N–C was close to FePc yet away from Fe foil, demonstrating again the presence of Fe–N bond. The peak at approximately 1.5 Å for (FeMn-DA)–N–C in Fig. 2h corresponds to the Mn-N bond, and the peak at 2.26 Å signified the Mn–Fe interaction. In order to obtain the specific active site structure of (FeMn-DA)–N–C, the least-squares EXAFS fitting was used. The R-space fitting diagram (Fig. 2g, h) and the K-space fitting diagram (Figs. S18 and S19) show that the structure in Fig. 2i exhibits the best fits. The fitting parameters are shown in Table S2, with coordination numbers of 4.06 ± 0.46, 4.59 ± 0.23, 0.7 ± 1.38, and 1.09 ± 0.56 for Fe–N, Mn–N, Fe–Mn, and Mn–Fe, respectively. Based on the above analysis, the (FeMn-DA)–N–C structure with the FeMn-N_6_ model is shown in Fig. 2i, in which four N atoms are coordinated with each Fe and Mn.

### Electrochemical Measurements

The ORR performance of the as-crafted catalysts was evaluated in acidic and alkaline environments using the rotating ring-disk electrode (RRDE) technique in a standard three-electrode system. First, we investigated the effects of temperature (Figs. S20–S25), the amount of metal added (Figs. S26 and S27), and different iron sources (Fig. S28) to identify the optimal experimental conditions.

**Fig. 3:**
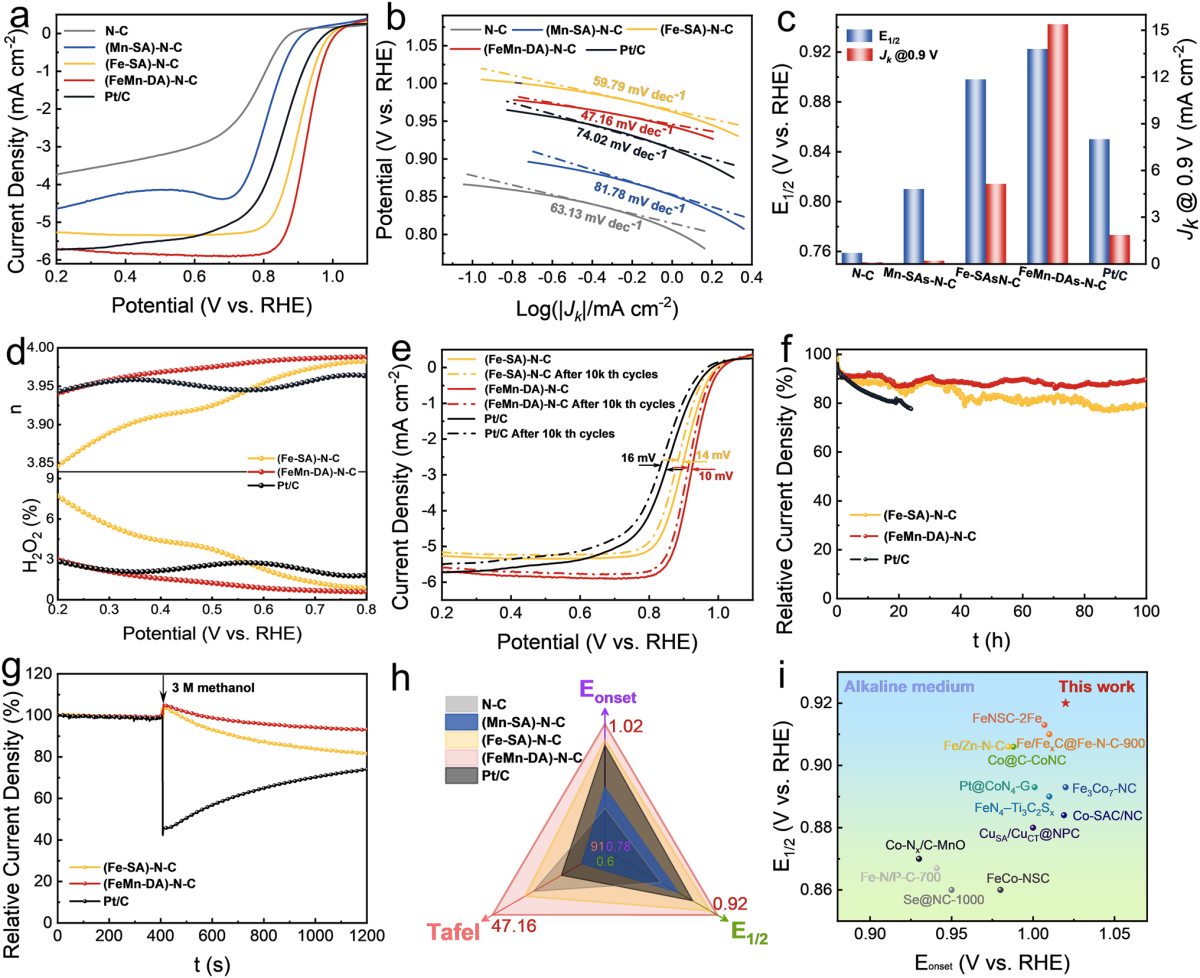
ORR performance in 0.1 M KOH. **a** Polarization curves. **b** Tafel slope. **c** *E*_1/2_ and *J*_*k*_. **d** H_2_O_2_% and *n*. **e** LSV curves after 10k th CV cycles at 0.6–1.1 V. **f** Chronoamperometric curves at 0.8 V (vs. RHE). **g** Methanol resistance. **h** Comparison of performance metrics of various catalysts prepared in this study and Pt/C. **i** Comparison of *E*_1/2_ and *E*_onset_ in this work with the previously reported catalysts in alkaline solution

The cyclic voltammetry (CV) study showed that (FeMn-DA)–N–C has the largest oxygen reduction peak in 0.1 M KOH, indicating it has the highest oxygen reduction potential (Fig. S29). Furthermore, the linear scanning voltammetry (LSV) measurements showed that (FeMn-DA)–N–C has the highest onset potential (*E*_onset_ = 1.02 V) and half-wave potential (*E*_1/2_ = 0.92 V) (Fig. 3a), compared to control samples and Pt/C, suggesting that the synergy of double atoms promotes the higher oxygen reduction activity. In addition, Fig. 3b displays a lower Tafel slope of (FeMn-DA)–N–C (47.16 mV dec^−1^) than control catalysts and Pt/C (74.02 mV dec^−1^), suggesting that it possesses a faster electron transfer rate and an enhanced ORR kinetics. The kinetic current density, *J*_*k*_, of (FeMn-DA)–N–C at 0.9 V was 15.42 mA cm^−2^ (Fig. 3c), threefold over that of (Fe-SA)–N–C and 8.3 times over that of Pt/C, further signifying that the (FeMn-DA)–N–C with the coexistence of Fe and Mn diatomic sites manifests greater ORR kinetics. The ring current and disk current were obtained by RRDE test (Fig. S30). The H_2_O_2_ yield (H_2_O_2_%) and electron transfer number, *n*, were calculated according to Eqs. (S3) and (S4) (Fig. 3d). The *n* of (FeMn-DA)–N–C was found to be in the range of 3.95–4, similar to that of Pt/C. Yet, the *n* of (Fe-SA)–N–C is in the range of 3.85–4. This result suggests that the ORR of (FeMn-DA)–N–C follows nearly a four-electron-transfer step, whereas a part of the reaction of (Fe-SA)–N–C proceeds via a two-electron-transfer step, which is in good agreement with *K*–*L* equation calculations (Fig. S31). The H_2_O_2_% of (FeMn-DA)–N–C was similar to that of commercial Pt/C (lower than 3%), and yet much lower than that of (Fe-SA)–N–C, indicating that the incorporation of Mn sites significantly reduced the H_2_O_2_% of the catalyst. The accelerated degradation test (ADT; Fig. 3e) and chronoamperometry (Fig. 3f) were used to evaluate the stability of the catalyst. The ADT tests reveal that after 10,000 CV cycles, the *E*_1/2_ losses of (Fe-SA)–N–C, (FeMn-DA)–N–C, and Pt/C were 14, 10, and 16 mV, respectively, signifying that (FeMn-DA)–N–C exhibited the best stability. The chronoamperometry results show that only 10.6% of the current loss after a 100-h stability test (Fig. 3f). This compares to a loss of 20.5% for the (Fe-SA)–N–C under the same conditions, and a loss of 22.2% for the Pt/C after only 24 h. Figure 3g presents that after injecting 3 M methanol in 0.1 M KOH, (FeMn-DA)–N–C and (Fe-SA)–N–C showed a less current attenuation over Pt/C, suggesting a higher methanol tolerance of the as-prepared catalysts. Figure 3h and Table S3 show the ORR of (FeMn-DA)–N–C outperformed various control catalysts prepared in this study and Pt/C. The ORR performance of (FeMn-DA)-N–C in 0.1 M KOH was found to be superior to most of the catalysts reported so far (Fig. 3i and Table S5).

**Fig. 4:**
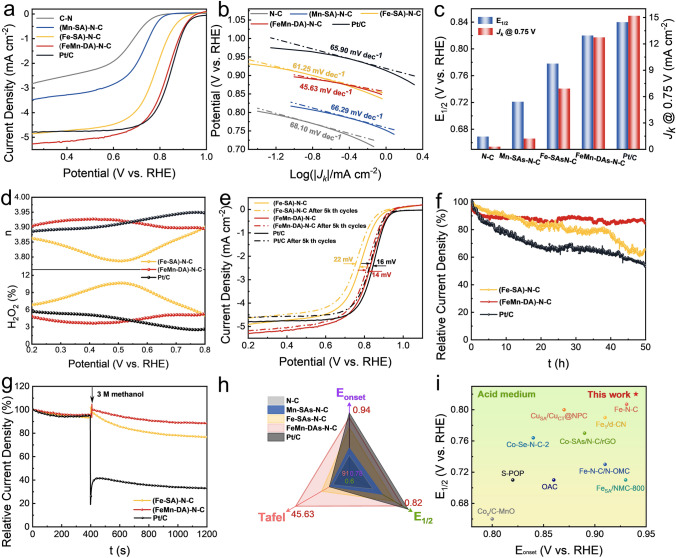
ORR performance in 0.1 M HClO_4_. **a** Polarization curves. **b** Tafel slope. **c** *E*_1/2_ and *J*_*k*_. **d** H_2_O_2_% and *n*. **e** LSV curves after 5k th CV cycles at 0.4–1.0 V. **f** Chronoamperometric curves at 0.5 V (vs. RHE). **g** Methanol resistance. **h** Comparison of metrics of as-prepared catalysts with control samples. **i** Comparison of ORR performance of the as-prepared catalysts with previously reported catalysts in acid solution

The oxygen reduction peak (Fig. S32), onset potential (*E*_onset_ = 0.94 V), and half-wave potential (*E*_1/2_ = 0.82 V; Fig. 4a) of (FeMn-DA)-N–C are larger than those of control catalysts prepared, and only slightly lower than those of Pt/C catalysts in 0.1 M HClO_4_. In addition, (FeMn-DA)–N–C exhibits a good ORR kinetics with the lowest Tafel slope (45.63 mV dec^−1^; Fig. 4b) and a slightly lower kinetic current density than Pt/C (*J*_*k*_ = 12.74 mA cm^−2^ at 0.75 V; Fig. 4c). The RRDE test results show that the *n* of (FeMn-DA)–N–C is approximately 3.9 (Fig. S33 and Fig. 4d), similar to that of Pt/C yet significantly larger than that of (Fe-SA)–N–C (similar to that calculated by *K–L* equation in Fig. S34). The H_2_O_2_% of (FeMn-DA)–N–C was similar to that of commercial Pt/C, below 6%, yet much lower than that of (Fe-SA)–N–C (6%-12%). It is well known that Fenton reaction between Fe^2+^ and H_2_O_2_ is more likely to occur under acidic conditions. It is notable that the addition of Mn markedly reduced the H_2_O_2_% and effectively prevented more Fenton reaction, thus effectively circumventing metal depletion. The ADT test results revealed that the *E*_1/2_ losses of (Fe-SA)–N–C, (FeMn-DA)–N–C, and Pt/C are 22, 14, and 16 mV, respectively, after 5k th CV cycles (Fig. 4e). The chronoamperometry study shows that after 50 h of testing, only 35%, 14%, and 46% of the current was lost for (Fe-SA)–N–C, (FeMn-DA)–N–C, and Pt/C, respectively (Fig. 4f), further demonstrating the effectiveness of introducing Mn in successfully avoiding demetallization and thus improving the stability of the catalyst. Notably, (FeMn-DA)–N–C displays an enhanced methanol tolerance over commercial Pt/C (Fig. 4g). The ORR performance of (FeMn-DA)–N–C outpaces the other prepared catalysts in this work (Fig. 4h and Table S4). Moreover, the as-prepared catalysts outperform the most of reported catalysts in acidic electrolytes (Fig. 4i and Table S6).

### Investigation into Reaction Mechanism via DFT Calculation

**Fig. 5:**
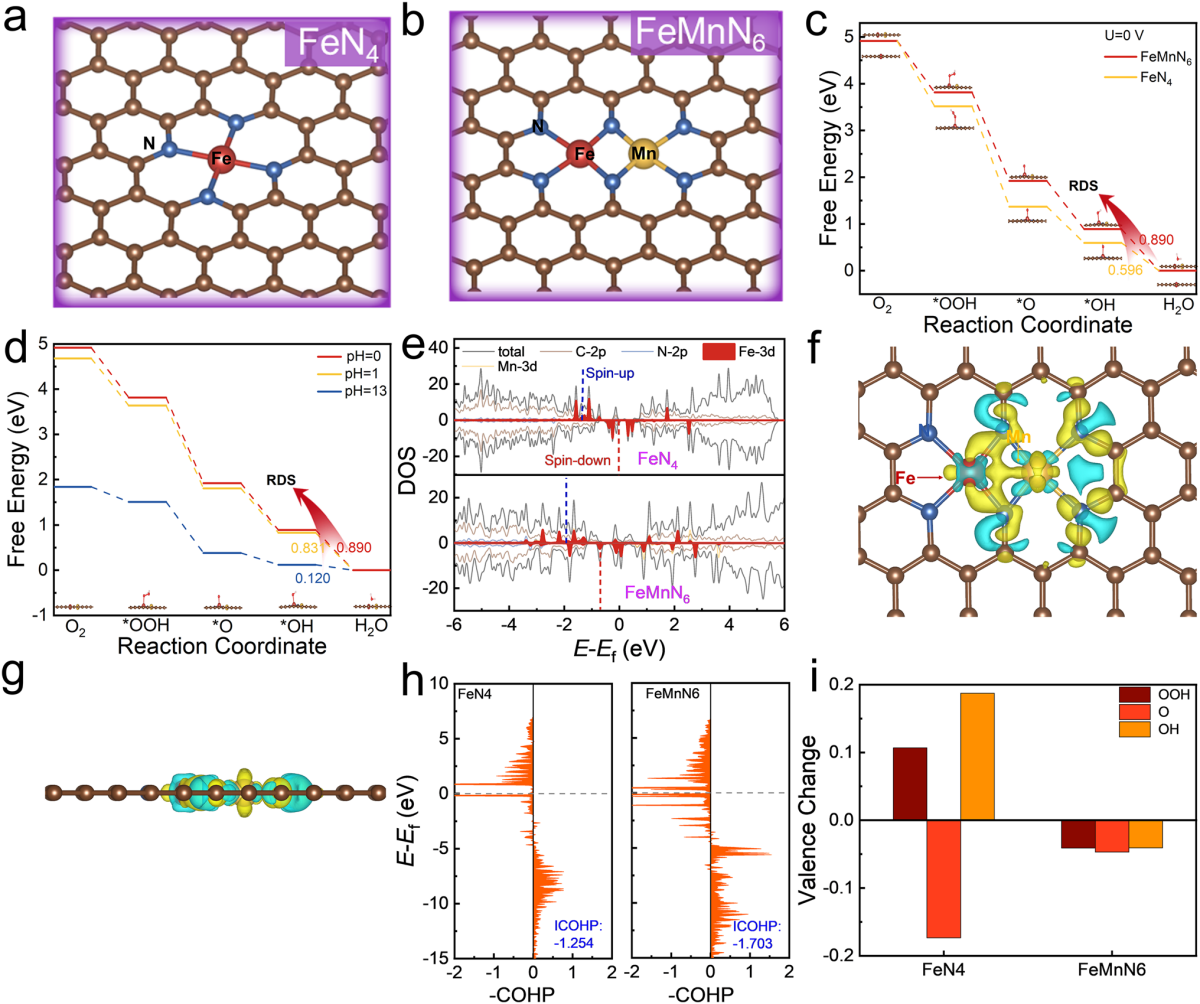
**a, b** Optimized FeN_4_ and FeMnN_6_ structures. The brown, blue, red, and yellow balls represent the C, N, Fe, and Mn atoms, respectively. **c** Free energy diagrams of ORR on FeN_4_ and FeMnN_6_. The limiting potentials are shown as the inserts. **d** Free energy diagram of different pH, where *OH → H_2_O is the RDS. **e** Density of states of FeN_4_ and FeMnN_6_. The *d*-band centers of the Fe atom are labeled with the red dashed lines. **f, g** Charge density of (**f**) top-view and (**g**) side-view of FeMnN_6_, where the yellow and blue clouds represent electron accumulation and deficiency, respectively. The isosurface value is 0.01 e Å^−3^. **h** COHP spectrum of FeN_4_ and FeMnN_6_. **i** Valence state change of Fe active site during ORR

DFT calculations were performed to scrutinize the reaction mechanism. The optimized structures of FeN_4_ and FeMnN_6_ are shown in Fig. 5a, b, respectively. Each metal atom binds with four nitrogen (N) atoms in FeMnN_6_, which is similar to that of Fe in FeN_4_. The Fe atom and Mn atom are bridged by two N atoms, signifying the possibility of indirect electron transfer between the two metal atoms through a M–N–M path. The corresponding length of different bonds is shown in Fig. S38.

The ORR begins with adsorption of oxygen molecules on metal sites, proceeding in two ways: (a) parallel to the graphite plane, where both O atoms bind to metal atoms, or (b) perpendicular to the graphite plane, where one O atom binds to Fe or Mn (Fig. S35a-c). The parallel structure, with an adsorption energy of − 1.135 eV, is the most stable (Table S7) and forms the basis for further reduction reactions.

As shown in Fig. S35d, proton reduction on O_Fe_ weakens the O–O bond, forming a Fe–OH + Mn–O structure, following the reverse O–O radical coupling mechanism (ORCM) with a rate-limiting step of OH desorption at 0.422 V (Table S8). Strong Mn–O binding lowers the adsorption free energy by 0.679 and 0.468 eV compared to Fe (Table S9), causing Mn site poisoning and making reverse ORCM unsuitable for ORR. As shown in Fig. S36, the AEM-Mn path mirrors the ORCM-O_Fe_ path (Fig. S35e), where OH desorption is the rate-limiting step, with a limiting potential of 0.422 V (Table S10). Similarly, the ORCM-O_Mn_ path has a limiting potential of 0.421 V (Table S11), also hindered by OH desorption on the Mn site. The strong Mn-OH bond impedes the reaction, making these pathways ineffective. In contrast, the AEM-Fe path, which bypasses Mn atom interaction, is the most favorable for ORR in our dual-metal system.

In-situ Raman results confirm the presence of OOH intermediates under both acidic and alkaline conditions (Fig. S37), whereas the ORCM-O_Fe_ and ORCM-O_Mn_ reaction paths in Fig. S35d discussed above are free of OOH intermediates (see Tables S8 and S11 for details). For the AEM-Mn path, the limiting potential can only reach 0.421 V, which is inconsistent with the experimental situation. Taken together, we confirmed that in the Fe and Mn dual sites, Mn plays the role of charge transfer, but does not participate in ORR as the active site.

Due to different electrochemical reaction paths in acidic and alkaline environments, specific reaction paths are shown in Eqs. (S9) and (S10). The intermediates of ORR include *OOH, *O, and *OH (Figs. S39 and S40).

The ORR Gibbs free energy changes were calculated. Figure 5c shows the rate-determining step (RDS) is the desorption of the OH group in both FeN_4_ and FeMnN_6_ structures. Figures S41 and S42 illustrate that this finding remains across various potentials. The Fe atom in FeMnN_6_ functions as the active site for ORR (Fig. 5b), and the Mn atom does not directly participate in the ORR process due to the insufficient adsorption of oxygen [48]. The limiting potentials of FeN_4_ and FeMnN_6_ are 0.890 and 0.596, respectively. It is worth noting that the adsorption energy of all intermediates in the dual-metal-atom structure is higher than that of single metal structure counterpart, which can be attributed to the attenuated reduction ability caused by the loss of valence electrons of Fe atoms. Figure 5d shows the change of Gibbs free energy at different pH. The results reveal that the change of pH does not alter the trend of ORR. On the other hand, the *d*-band center of the Fe active site is lower in FeMnN_6_ than FeN_4_ (Fig. 5e, − 1.926 eV for spin-up and − 0.690 eV for spin-down in FeMnN_6_, and − 1.810 eV for spin-up and − 0.034 eV for spin-down in FeN_4_; obtained from Eq. (S11)), indicates that the anti-bonding state between Fe atom and intermediates is more likely to be located below the Fermi energy level and filled by electrons, leading to a higher adsorption energy of OH group (the intermediate of RDS) and improved the limiting potential of FeMnN_6_. As illustrated in the charge density difference diagram (Figs. 5f, g, S43, and S44), the valence electrons of Fe partially transfer to the MnN_4_ region, which is further substantiated by the increasing Fe valence state and decreasing N valence state compared to the FeN_4_ structure (Table S12). This result correlates well with the XPS study discussed above (Fig. 2d).

The stability differences are examined from the following perspectives: (1) the formation energy difference; (2) the bonding characteristics between active site; (3) the adjacent N atoms, and valence state variations of active site during reaction.

1. The formation energy difference was firstly calculated according to Eq. (S12). The chemical potential of each element is shown in Table S16. The formation energy of FeMnN_6_ is 0.088 eV, less than that of FeN_4_, suggesting that the FeMnN_6_ structure is more stable.
2. The LOBSTER program [49, 50] was employed to calculate the COHP and ICOHP of the Fe–N bonds, to evaluate the stability of FeN_4_ and FeMnN_6_. The COHP spectrum is shown in Fig. 5h. The Fe–N bonds in the two systems exhibit the bonding characteristics in the low-energy region, while anti-bonding characteristics appear around the Fermi level. The ICOHP can quantitatively assess the bonding characteristics of system. The more negative the value, the more pronounced the bonding interactions, indicating greater stability of the system. The ICOHP results are − 1.254 and − 1.703 for FeN_4_ and FeMnN_6_, respectively, suggesting enhanced stability of the FeMnN_6_ system.
3. The variation in the valence states of Fe active site during ORR can be obtained through the Bader analysis. As shown in Fig. 5i, the valence state of Fe atom exhibits notable variation upon adsorption of intermediates in FeN_4_, with a maximum difference of 0.361 eV (between *O and *OH), as the value decreases to 0.041 eV in the FeMnN_6_ system (between *O and *OH). This is mainly attributed to the insertion of Mn atoms, bringing additional valence electrons to maintain the chemical environment of Fe active site during the reaction process and improves the stability of the catalytic system.

### Performance of Fuel Cells

To further explore the application of the as-prepared ORR catalysts for practical AEMFC and PEMFC, (Fe-SA)–N–C and (FeMn-DA)–N–C were used as cathodic oxygen reduction catalysts to construct membrane electrode assemblies (MEA; Figs. S45–S47).

**Fig. 6:**
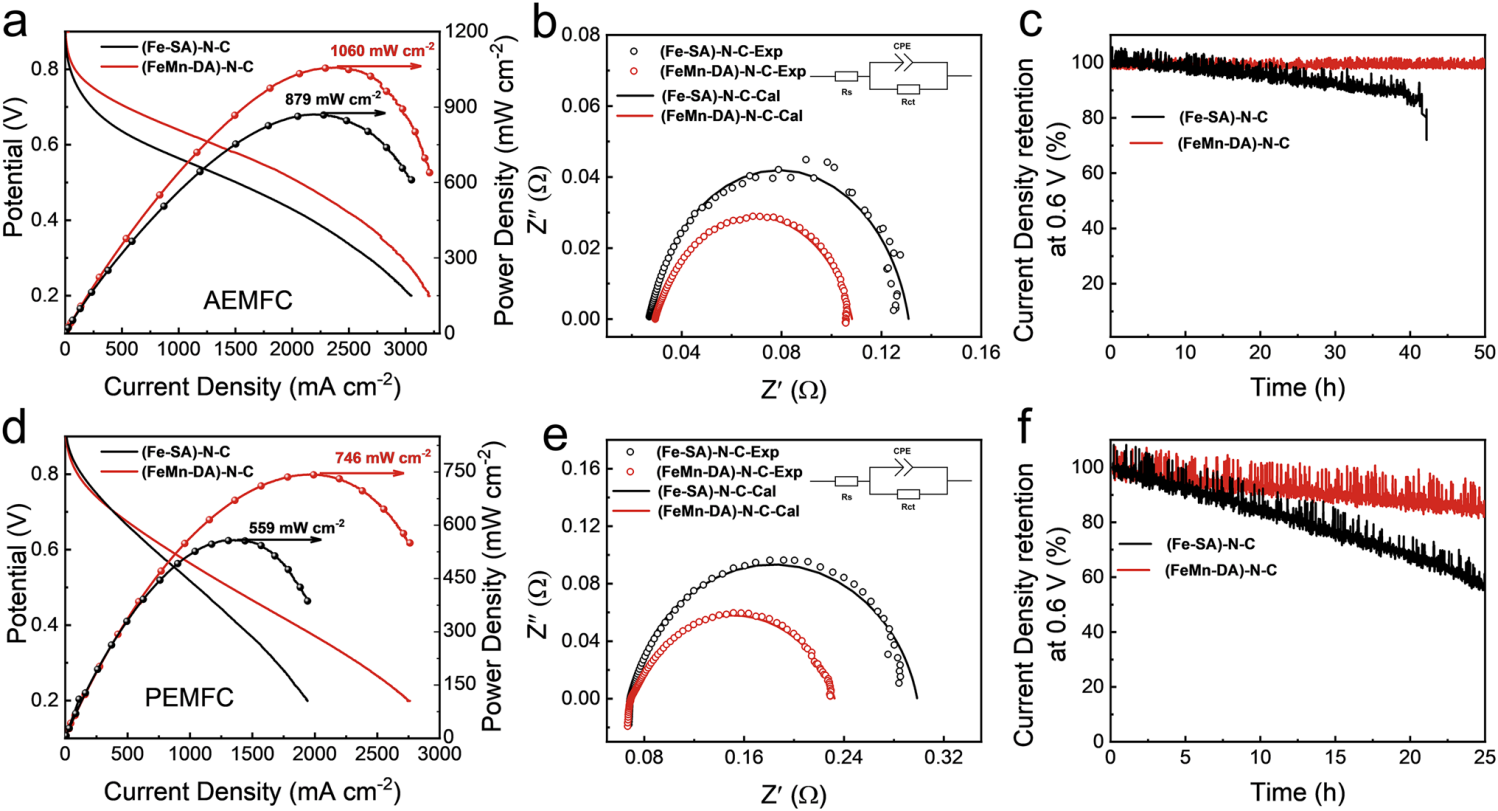
**a** Polarization and power density plots of H_2_−O_2_ AEMFC in alkaline environment with (Fe-SA)–N–C and (FeMn-DA)–N–C as cathode catalysts. **b** Nyquist plots of AEMFC using (Fe-SA)–N–C and (FeMn-DA)–N–C as the cathode catalysts at 0.6 V (*R*_s_: ohmic resistance; *R*_ct_: charge-transfer resistance; CPE: constant phase angle element). **c** Corresponding stability test at a constant voltage of 0.6 V. Test condition: Area, 4 cm^2^; Cathode loading, 3.5 mg cm^−2^; Anode loading, 0.2 mg_pt_ cm^−2^; Back pressure, 2 bar; Membrane, PAP-TP-85; Flow rate, 200 sccm for H_2_ and 500 sccm for O_2_; Cell temperature, 80 °C; Humidify, 100%. **d** Polarization and power density plots of H_2_−O_2_ PEMFC for acid environment with (Fe-SA)–N–C and (FeMn-DA)–N–C as cathode catalysts. **e** Nyquist plots of PEMFC using (Fe-SA)–N–C and (FeMn-DA)–N–C as the cathode catalysts at 0.6 V (*R*_s_: ohmic resistance; *R*_ct_: charge-transfer resistance; CPE: constant phase angle element).** f** Corresponding stability test at constant voltage of 0.6 V. Test condition: Cathode loading, 1 mg cm^−2^; Anode loading, 0.4 mg_PtRu_ cm^−2^; Membrane, Nafion 212. Other conditions are the same as PEMFC

Figure 6a shows the polarization and power density curves of AEMFC by capitalizing on (FeMn-DA)–N–C and (Fe-SA)–N–C as cathodic oxygen reduction catalysts. The AEMFC using (FeMn-DA)–N–C catalyst reaches a current density of 1332 mA cm^−2^ at 0.6 V. In contrast, (Fe-SA)–N–C catalyst only delivers a current density of 732 mA cm^−2^ at 0.6 V. At 0.2 V, the (FeMn-DA)–N–C-based AEMFC has a current density of 3203 mA cm^−2^. Yet, it is 3047 mA cm^−2^ for the (Fe-SA)–N–C-based AEMFC. Under the same test condition, the membrane electrode with (FeMn-DA)–N–C as the cathodic oxygen reduction catalyst achieves a peak power density of 1060 mW cm^−2^, higher than 879 mW cm^−2^ for the (Fe-SA)–N–C-based counterpart. The electrochemical impedance spectroscopy (EIS) measurements were conducted on AEMFC systems (Fig. 6b), utilizing (Fe-SA)–N–C and (FeMn-DA)–N–C catalysts, under an applied potential of 0.6 V. The corresponding fitted data are presented in Table S17. The results indicate that the ohmic resistance (*R*_s_) of both (Fe-SA)–N–C and (FeMn-DA)–N–C are comparable in both AEMFC systems. However, the charge-transfer resistance (*R*_ct_) of (FeMn-DA)–N–C is markedly lower than that of (Fe-SA)–N–C, suggesting an enhanced reaction kinetics in the (FeMn-DA)–N–C-based AEMFC. This demonstrates that the (FeMn-DA)–N–C-based fuel cell exhibits a faster reaction kinetics. The stability of the corresponding AEMFC was further evaluated under a constant applied voltage of 0.6 V (Fig. 6c). The results demonstrate that the AEMFC employing (FeMn-DA)–N–C as the cathode catalyst maintains a 99.6% retention in current density after 50 h, whereas the AEMFC utilizing (Fe-SA)–N–C as the cathode catalyst experiences a reduction in current density retention to 71% after only 42 h.

Figure 6d shows the polarization and power density curves of PEMFC assembled using (FeMn-DA)–N–C and (Fe-SA)–N–C as cathodic oxygen reduction catalysts. The current densities of (FeMn-DA)–N–C as ORR catalysts for membrane electrode are 835 and 2753 mA cm^−2^ at 0.6 and 0.2 V, respectively, greater than (Fe-SA)–N–C as ORR catalysts (710 and 1933 mA cm^−2^, respectively). Moreover, the peak power density of (FeMn-DA)–N–C-based MEA (746 mW cm^−2^) is larger than that of (Fe-SA)-N–C-based MEA (559 mW cm^−2^). For the EIS test, the results of the AEMFC test were similar. The results in Fig. 6e and Table S18 showed that the *R*_ct_ of the (FeMn-DA)–N–C catalysts was larger and showed a higher reaction rate. We also tested the stability of the corresponding PEMFC at a constant voltage of 0.6 V (Fig. 6f). The results show that the current density retention of PEMFC with (FeMn-DA)–N–C as the cathode catalyst is 84.3% after 25 h, while the current density retention of PEMFC with (Fe-SA)-N–C as the cathode catalyst is reduced to 56.8% after 25 h.

More importantly, the ORR catalyst synthesized by Hemin shows a better performance of fuel cells. As shown in Fig. S48, the peak power densities of AEMFC using FeCl_3_·6H_2_O-(Fe-SA)–N–C and FeCl_3_·6H_2_O-(FeMn-DA)–N–C as cathode catalysts are 540 and 652 mW cm^−2^, respectively. The corresponding peak power densities of PEMFC are 478 and 560 mW cm^−2^, respectively.

The fuel cell test results reveal that Fe and Mn synergistically customize the local environment and markedly improve the MEA performance in acidic and alkaline media. Meanwhile, our rational design of catalysts renders them with simultaneous presence of micropores, mesopores and macropores, which plays an important role in the complex heat and mass transfer processes of fuel cells. Clearly, the (FeMn-DA)–N–C catalysts outperformed the ORR catalysts reported in the literature for both AEMFCs and PEMFCs (Tables S19 and S20).

## Conclusion

In summary, we report the crafting of atomically dispersed Fe–Mn dual-metal atoms on N-doped carbon (i.e., (FeMn-DA)–N–C), via a doping-adsorption-pyrolysis approach, as ORR catalysts with concurrently optimized activity and stability for anion-exchange membrane and proton exchange membrane fuel cells. The Mn incorporation altered the electronic structure of Fe, promoting the OH group desorption, raising the limiting potentials, and shifting the position of *d*-band iron active center, as revealed by DFT calculations. Such rationally designed (FeMn-DA)–N–C catalysts exhibit a half-wave potential of 0.92 and 0.82 V in alkaline and acidic environments, respectively, along with outstanding stability. The Mn addition increases the density of active sites and stabilizes Fe. Consequently, the aggregation of Fe and Fenton reaction are effectively prevented, thereby reducing Fe consumption. Moreover, (FeMn-DA)–N–C-based anion/proton exchange membrane fuel cells manifest high power densities (1060 and 746 mW cm^−2^), highlighting their potential for practical application. The judicious synthesis of Fe-containing dual-atom ORR catalysts with simultaneously high activity and stability stands out as a robust route to leveraging their utilities in a wide range of energy conversion and storage materials and devices.

## Supplementary Information

Below is the link to the electronic supplementary material.

Supplementary file1 (DOCX 37324 kb)
