# Supplementary material for: Concurrently Boosting Activity and Stability of Oxygen Reduction Reaction Catalysts via Judiciously Crafting Fe–Mn Dual Atoms for Fuel Cells
Source: Nanomicro Lett. 2024 Dec 16;17:88. doi: 10.1007/s40820-024-01580-5 (PMC11646968; doi:10.1007/s40820-024-01580-5)
Supplement: Supplementary file 1 — Supplementary file1 (DOCX 37324 kb) [file 40820_2024_1580_MOESM1_ESM.docx]

Supporting Information for

**Concurrently Boosting Activity and Stability of Oxygen Reduction Reaction Catalysts via Judiciously Crafting Fe-Mn Dual Atoms for Fuel Cells**

Lei Zhang^1,3^, Yuchen Dong^1^, Lubing Li^1^, Yuchuan Shi^1^, Yan Zhang^1^, Liting Wei^1^, Chung-Li Dong^2^, Zhiqun Lin^3,^*, and Jinzhan Su^1,^*

^1^International Research Center for Renewable Energy, State Key Laboratory of Multiphase Flow in Power Engineering, Xi’an Jiaotong University, Xi’an 710049, P. R. China

^2^ Department of Physics, Tamkang University, New Taipei City, Taiwan 25137, P. R. China

^3^ Department of Chemical and Biomolecular Engineering, National University of Singapore, Engineering Drive 4, Singapore 117585, Singapore

*Corresponding authors. E-mail: [j.su@mail.xjtu.edu.cn](mailto:j.su@mail.xjtu.edu.cn) (Jinzhan Su); [z.lin@nus.edu.sg](mailto:z.lin@nus.edu.sg) (Zhiqun Lin)

**S1 Experimental Section**

**S1.1 Materials**

Zinc nitrate hexahydrate (Zn (NO_3_)_2_·6H_2_O, 98%)-Thermo Scientific, Manganese (Ⅱ) acetate tetrahydrate (Mn (OAc)_2_·4H_2_O, 99%)-Macklin, 2-methylimidazole (2-MeIM, 99%)-Thermo Scientific, Hemin chloride (Hemin, 99%)-Macklin. Methanol, N, N-Dimethylformamide (DMF), isopropanol and Potassium hydroxide (KOH) were obtained from Shanghai Chemical Reagents, China (AR). 0.1 M Perchloric acid (HClO_4_) were obtained from Aladdin. Commercial Pt/C catalyst (20 wt%, Johnson Matthey, HiSPEC 3000) was purchased from Hesen, Shanghai. The deionized water was obtained from a Millipore ultrapure system (18.25 MΩ, Heng Chun, China). All materials were of analytical grade and were used without further purification. Nafion was obtained from Thermo Scientific.

**S1.2 Material Synthesis**

***S1.2.1 Synthesis of Mn-ZIF-8***

2.79 g of Zn (NO_3_)_2_·6H_2_O were dissolved in 75 mL methanol, then 3.08 g 2-MeIM in 75 mL methanol was subsequently injected into the above solution and aged for 24 h at room temperature. The as-obtained precipitates were centrifuged, washed with methanol several times and dried under vacuum at 343 K for 10 h to obtain ZIF-8.

***S1.2.2 Synthesis of Mn-ZIF-8***

2.79 g of Zn (NO_3_)_2_·6H_2_O and 0.558, 1.116, 2.232 g Mn (OAc)_2_·4H_2_O were dissolved in 75 mL methanol, then 3.08 g 2-MeIM in 75 mL methanol was subsequently injected into the above solution and aged for 24 h at room temperature. The as-obtained precipitates were centrifuged, washed with methanol several times and dried under vacuum at 343 K for 10 h to obtain Mn-ZIF-8.

***S1.2.3 Synthesis of ZIF-8@Hemin***

0.5 g ZIF-8 was dissolved in 100 mL DMF, then 25, 50, 100 mg Hemin in 100 mL DMF was subsequently injected into the above solution and aged for 24 h at room temperature. The as-obtained precipitates were centrifuged, washed with methanol several times and dried under vacuum at 343 K for 10 h to obtain ZIF-8@ Hemin.

***S1.2.4 Synthesis of Mn-ZIF-8@Hemin***

0.5 g Mn-ZIF-8 was dissolved in 100 mL DMF, then 50 mg Hemin in 100 mL DMF was subsequently injected into the above solution and aged for 24 h at room temperature. The as-obtained precipitates were centrifuged, washed with methanol several times and dried under vacuum at 343 K for 10 h to obtain Mn-ZIF-8@ Hemin.

***S1.2.5 Synthesis of*** ***(Fe-SA)-N-C, (Mn-SA)-N-C, and (FeMn-DA)-N-C***

The as-synthesized Mn-ZIF-8@Hemin were placed in a tube furnace and then heated to 1193 K for 2 h at the heating rate of 275 K min^-1^ under flowing Ar gas and then naturally cooled to room temperature to obtain samples of (FeMn-DA)-N-C (Other conditions being equal, we chose pyrolysis temperatures of 1173 and 1223 K as controls to explore the effect of temperature).

The synthesis procedure of (Fe-SA)-N-C was the same as that of (FeMn-DA)-N-C, except for replacing Mn-ZIF-8@Hemin with ZIF-8@Hemin; The synthesis procedure of (Mn-SA)-N-C was the same as that of (FeMn-DA)-N-C, except for not adding Hemin. To explore the effect of metal source on catalytic performance, we replace Hemin with equal molar amount of FeCl_3_·6H_2_O under the condition that other conditions remain unchanged.

**S1.3 Characterization**

Powder X-ray diffraction (XRD) data were acquired on a PANalytical X'pert MPD PRO X-ray diffractometer (Cu Ka radiation 45 kV,40 mA). The scanning electron microscope (SEM) images of the samples were obtained from JEOL JSM-7800FE, Japan. Transmission electron microscope (TEM) images were received by a JEOL JEM-F200 (HR) field emission at 200 kV. The energy-dispersive X-ray spectroscopy (EDS) mapping was obtained on a scanning transmission electron microscope with a high-angle annular-dark-field (HAADF-STEM) detector (OXFORD MAX-80). The aberration-corrected transmission electron microscopy (AC-TEM) images were recorded by a JEM-ARM200F at 200 kV. The X-ray photoelectron spectroscopy (XPS) measurement was performed on an X-ray photoelectron spectrometer from Kratos Axis Ultra DLD, Japan. Inductively coupled plasma (ICP) data were obtained by using a NexION ICP-MS 350D instrument. N_2_ adsorption/desorption isotherm at 77 K was measured by an ASAP 2460 analyzer (Micromeritics, U.S.A). The specific surface area and pore size distribution were evaluated by Brunauer-Emmett-Teller (BET) and non-local density functional theory (NLDFT). The Raman spectroscopies were recorded with a LabRAM HR Evolution spectrometer using the 523 nm line for excitation.

The X-ray absorption near-edge structure (XANES) and extended X-ray absorption fine structure (EXAFS) spectra were carried out at BL20A at the National Synchrotron Radiation Research Center, Taiwan. The Fe K-edge/Mn K-edge XANES data were recorded in a transmission mode. Fe foil, FeO, Fe_2_O_3_, Fe_3_O_4_, and FePc (Mn foil, MnO and Mn_2_O_3_, Mn_3_O_4_) were used as references. The acquired EXAFS data were extracted and processed according to the standard procedures using the ATHENA module implemented in the IFEFFIT software packages. The k^3^-weighted EXAFS spectra were obtained by subtracting the post-edge background from the overall absorption and then normalizing concerning the edge-jump step. Subsequently, k^3^-weighted χ(k) data in the k-space ranging from 3–11 Å^−1^ were Fourier transformed to real (R) space using hanging windows (dK = 1.0 Å^−1^) to separate the EXAFS contributions from different coordination shells.

***S1.4 Electrochemical Measurement***

Electrochemical performances were evaluated through the CHI 760E electrochemical workstation (CH Instruments, Shanghai, China) using a three-electrode setup with the electrolyte of 0.1 M KOH or 0.1 M HClO_4_ aqueous solution and rotating ring disk electrode (RRDE, Pine Instruments) at ambient conditions. The working electrode was a glassy carbon rotating disk electrode (RDE) with a diameter of 5.0 mm or a rotating ring disk electrode (RRDE) with a Pt ring (5.6 mm inner diameter and 6.6 mm outer diameter) as the substrate for the catalyst samples. The Hg/HgO electrode and Ag/AgCl electrode were used as reference electrodes for acidic and alkaline electrolyte, respectively. The platinum wire was used as the electrode. The non-noble-metal catalyst inks were prepared by blending 2 mg of the as-synthesized catalyst with 238 μL isopropanol, 238 μL DI tater and 25 μL Nafion solution (5 wt%) and sonicated for 30 min. Then, 24 μL of catalyst dispersion was deposited onto the GC surface, leading to the loading mass of 0.488 mg cm^-2^. The 20% Pt/C ink as a comparison was prepared by dispersing 1 mg of Pt/C in 0.5 mL solution (240 μL isopropanol, 240 μL DI water and 15 μL Nafion solution (5 wt%)) following an ultrasonic bath for 30 min. 25 μL of Pt/C ink was then pipetted onto the GC surface, leading to the loading mass of 0.244 mg cm^-2^. The electrodes were activated by 50 cycles cyclic voltammetry curves (CV) with a faster scan rate of 50 mV s^−1^ conducted in O_2_/N_2_ saturated 0.1 M KOH or 0.1 M HClO_4_ electrolyte and the electrolyte should be purged O_2_ for at least 30 min before testing. Cyclic voltammetry (CV) curves were measured at a scanning rate of 5 mV s^−1^. The linear sweep voltammetry (LSV) tests were performed in an O_2_-saturated electrolyte with a scan rate of 5 mV s^–1^ and a rotation rate of 1600 rpm. Chronoamperometry (CA) and Tafel tests were conducted in an O_2_-saturated electrolyte at a rotation rate of 1600 rpm.

All potentials in this work were converted to the reversible hydrogen electrode (RHE) by the formula:

$E\left( vs.RHE \right)=E\left( vs.Hg/HgO \right)+0.059\times PH+0.098$  (S1)

$E\left( vs.RHE \right)=E\left( vs.Ag/AgCl \right)+0.059\times PH+0.197$ (S2)

The electron-transfer numbers (n) and kinetic current density were calculated according to the Koutechy-Levich (K-L) equation:

$\frac{1}{J}=\frac{1}{J_{k}}+\frac{1}{J_{L}}=\frac{1}{Bw^{\frac{1}{2}}}+\frac{1}{J_{k}}$ (S3)

$B=0.62nFC_{0}D_{0}^{\frac{2}{3}}v^{-\frac{1}{6}}$ (S4)

where *J*, *J_k_*, and *J_L_* are the measured current density, kinetic current density and diffusion-limited current density, respectively; $w$ is the angular velocity of the rotating electrode; *F* is the Faraday constant (*F*=96485 C mol^-1^), and *n* is the electron-transfer rate constant. *C_0_* is the bulk concentration of O_2_ (*C_0_*=1.2×10^-6^ mol cm^-3^); *D_0_* is the diffusion coefficient of O_2_ (*D_0_*=1.9×10^-5^ cm^-2^ s^-1^), and *v* is the kinematic viscosity (*v*=0.01 cm^-2^ s^-1^).

The RRDE examinations were carried out by testing the ring current (*I*_Ring_) with the Pt ring potential at 1.3 V (*vs.* RHE). The peroxide yield (H_2_O_2_%) and the electron transfer number (n) were calculated by

$n=4\times\frac{I_{Disk}}{N^{-1}I_{Ring}+I_{Disk}}$ (S5)

$H_{2}O_{2}\%=200\times\frac{I_{Ring}}{I_{Ring}+{NI}_{Disk}}$ (S6)

where *I_Disk_* is disk current, *I_Ring_* is ring current, and N is the current collection efficiency of the Pt ring, which is provided as 0.37 by the manufacturer.

The ORR stability of the electrocatalysts was first examined in the O_2_-staurated 0.1 M KOH electrolyte by accelerated deterioration tests (ADT) at a scan rate of 100 mV s^-1^ for 30,000 cycles between 0.6 and 1.0 V (vs. RHE). The ORR stability in O_2_-staurated 0.1 M KOH was also performed by chronoamperometry at a potential of 0.70 V (vs. RHE).

**S1.5 Computational Method**

Spin-polarized density functional theory (DFT) calculations with the projector-augmented wave (PAW) method [S1, S2] were performed using the Vienna Ab-initio Simulation Package (VASP) [S3, S4]. The plane wave basis set with a cutoff energy of 600 and 500 eV was applied to the geometric optimization and electronic structure calculations, respectively. The electronic exchange and correlation effects were described within the generalized gradient approximation (GGA) in the Perdew-Burke-Ernzerhof (PBE) form [S5]. The C 2s^2^2p^2^, N 2s^2^2p^3^, Fe 3d^6^4s^2^, and Mn 3p^6^3d^5^4s^2^ electrons were treated as valence electrons. The convergence thresholds were set to be 10^-5^ eV for energy, and 0.02 eV/Å for force. The partial occupancies were determined by the Gaussian smearing method with a smearing width of 0.05 eV. The dispersion correction with the BJ-damping function was included in all calculations [S6, S7].

The FeMnN_6_ and FeN_4_ structures were constructed by modifying a P6/MMM symmetrized monolayer graphene. The a and b lattice parameters were allowed to be relaxed during the geometric optimization processes, while the direction perpendicular to the graphene plane was fixed with a 16 Å vacuum layer to eliminate the spurious interaction of neighboring supercells. The lattice parameters of the optimized FeMnN_6_ and FeN_4_ structures were 14.64 and 14.72 Å, respectively. The Brillouin zone was sampled by a 2×2×1 or 4×4×1 *k*-point mesh for geometric or electronic structure calculations within the Γ-centered Monkhorst-Pack method.

The charge density difference was calculated according to the following equation:

$\rho_{\mathrm{diff}}=\rho_{FeMnN6}-\rho_{MnN4}-\rho_{\mathrm{rest}}$ (S7)

where the subscripts FeMnN_6_, MnN_4_, and rest represent the intact FeMnN_6_ structure, the Mn atom with the 4 neighboring N atoms, and all of the rest atoms, respectively.

The thermal correction was obtained using VASPKIT software [S8] after vibrational frequency calculation, in the form of

$\Delta G=E_{\mathrm{ZPE}}+\Delta U_{0\to T}-T\Delta S$ (S8)

where $E_{\mathrm{ZPE}}$ is the zero-point vibration energy, $\Delta U_{0\to T}$ is the internal energy change between ground state to specific temperature $T$, and $T\Delta S$ is the entropic contributions. Only the adsorbed intermediates (i.e., *OOH, *O, and *OH) were allowed to vibrate in the frequency calculations.

In an alkaline medium, the ORR mechanisms for 4e^−^ processes including the intermediates are (* denotes active sites):

$$*+O_{2}+H_{2}O+e^{-}\to*OOH+OH^{-}$$

$$*OOH+e^{-}\to*O+OH^{-}$$

$$*O+H_{2}O+e^{-}\to*OH+OH^{-}$$

$*OH+e^{-}\to*+OH^{-}$ (S9)

In an acid medium, the ORR mechanisms for 4e^−^ processes including the intermediates are:

$$*+O_{2}+H^{+}+e^{-}\to*OOH$$

$$*OOH+H^{+}+e^{-}\to*O+H_{2}O$$

$$*O+H^{+}+e^{-}\to*OH$$

$*OH+H^{+}+e^{-}\to*O+H_{2}O$ (S10)

The DOS projected onto the *d*-states that interact with the adsorbate state can be characterized by the moments of the d DOS. The first moment is the *d*-band center:

${}_{d}=\frac{\int_{-}^{+} n_{d}\left( \right) d}{\int_{-}^{+} \left( \right)d}$ (S11)

The formation energy difference is firstly calculated based on the chemical potential:

$\Delta E_{\mathrm{formation}}=E_{FeMnN6}-E_{FeN4}-\sum_{i} n_{i}\mu_{i}$ (S12)

$\mathrm{where}n_{i}$ is the number of atoms added (positive) or removed (negative).

**S1.6 MEA Performance Measurement**

Membrane-electrode assemblies (MEAs) were prepared as described in our previous work [S9, S10].

***Proton exchange membrane fuel cell (PEMFC):*** The cathode catalyst ink was obtained by stirring 40 mg catalyst, 606 μL 5 wt% Nafion®, 5.7 mL isopropyl alcohol and 5.7 mL DI water. The homogeneous ink was obtained by using an ultrasonic treatment in an ice bath for 3 h. And the anode catalyst was obtained by mixing 15 mg Pt/C catalyst (20 wt%, Jothson Matthey, JM), 100 μL 5 wt% Nafion®, 2.2 mL isopropyl alcohol and 2.2 mL DI water for 6 h. The ink was transferred on the Nafion® 211 membrane (4 cm^2^) using a spray pen (Ustar S130). The Toray 060 carbon paper (20% PTFE hydrophobic) was used as gas diffusion layer (GDL). Graphite plates with parallel flow fields are used as flow field plates. The catalyst loading of cathode and anode were 3.5 and 0.2 mg·cm^-2^. Membrane electrode assembly (MEA) was prepared by the catalyst coated membrane (CCM) and GDL heated by hot pressing at 120 °C and 0.3 MPa for 2 min. Single cell performance was tested on a fuel cell testing station (Radbee, FCS-100W-SC-EL-ZR-Hermés) with an electrochemical workstation (Biologic, HCP-803).

***Anion exchange membrane fuel cell (AEMFC):*** For the AEMFC, the catalyst ink was prepared by ultrasonically dispersing the catalysts and PAP-TP-100 (5 wt% in ethanol) into water and isopropanol (1:25 v/v) for 2.0 h. The anode catalyst was 60 wt% PtRu/C (Johnson Matthey). Then the catalyst ink was sprayed onto both sides of PAP-TP-85 membrane (15 μm) to fabricate a catalyst-coated membrane (CCM). The catalyst loading of cathode and anode were 0.8 mg·cm^-2^ and 0.4 mg_PtRu_·cm^-2^. All CCMs were immersed into 3 M KOH solution for 2.0 h (exchange the solution every 1.0 h) and then rinsed thoroughly with deionized water to remove all excess KOH. A cold-press procedure was used to realize the MEAs. The other process was the same as PEMFC.

**Supplementary Figures and Tables**


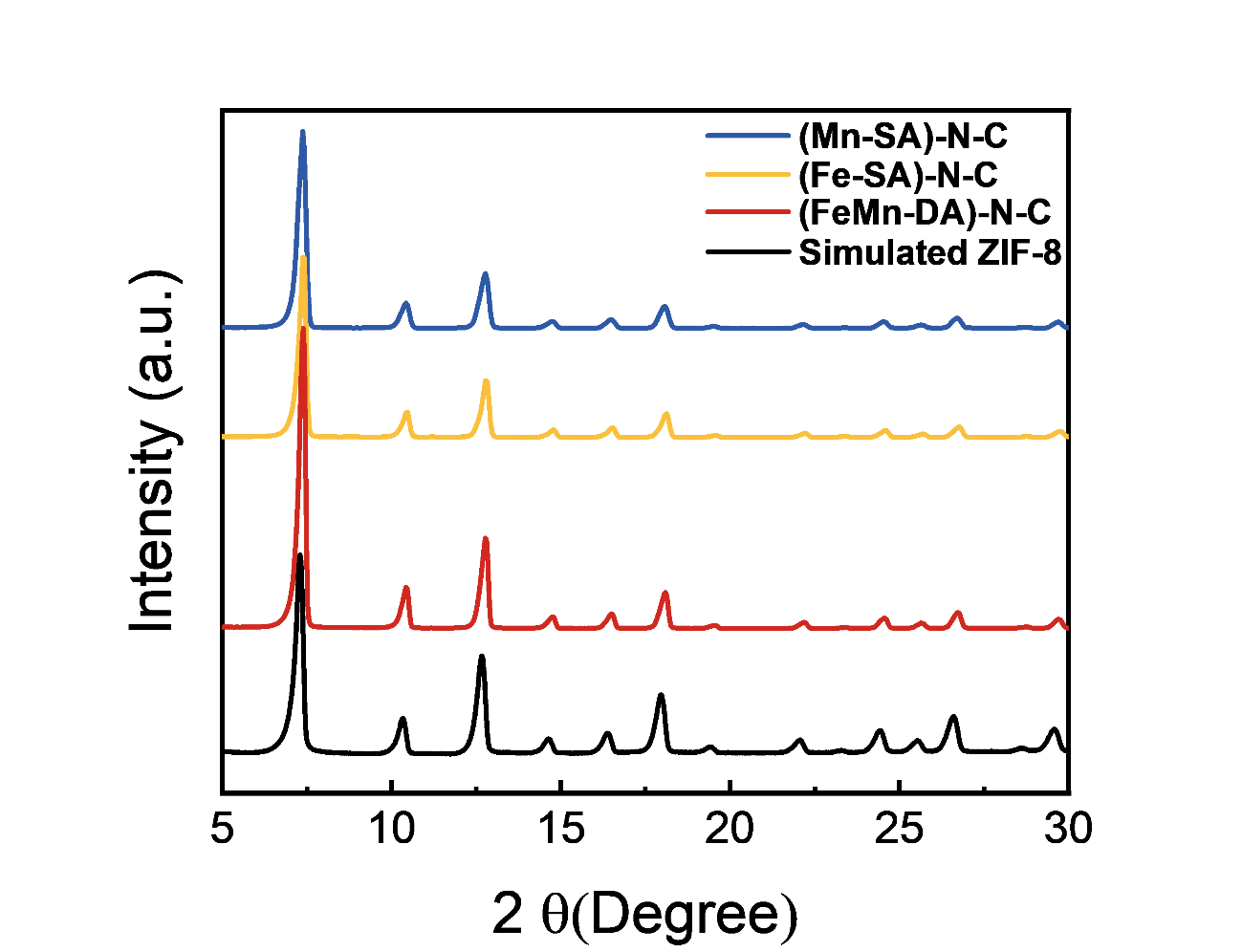


**Fig. S1** XRD patterns of (Mn-SA)-N-C, (Fe-SA)-N-C and (FeMn-DA)-N-C before pyrolysis


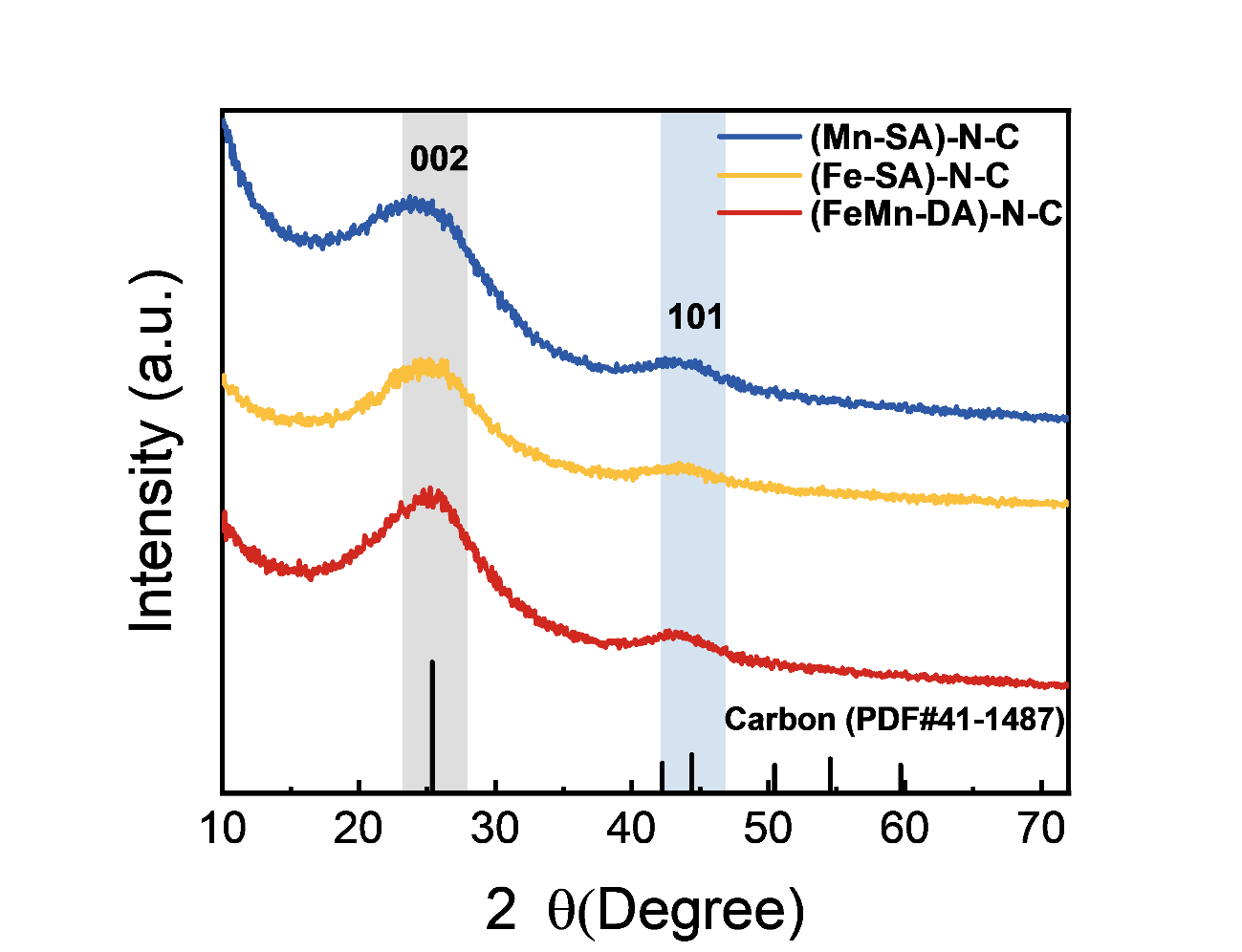


**Fig. S2** XRD patterns of (Mn-SA)-N-C, (Fe-SA)-N-C and (FeMn-DA)-N-C after pyrolysis


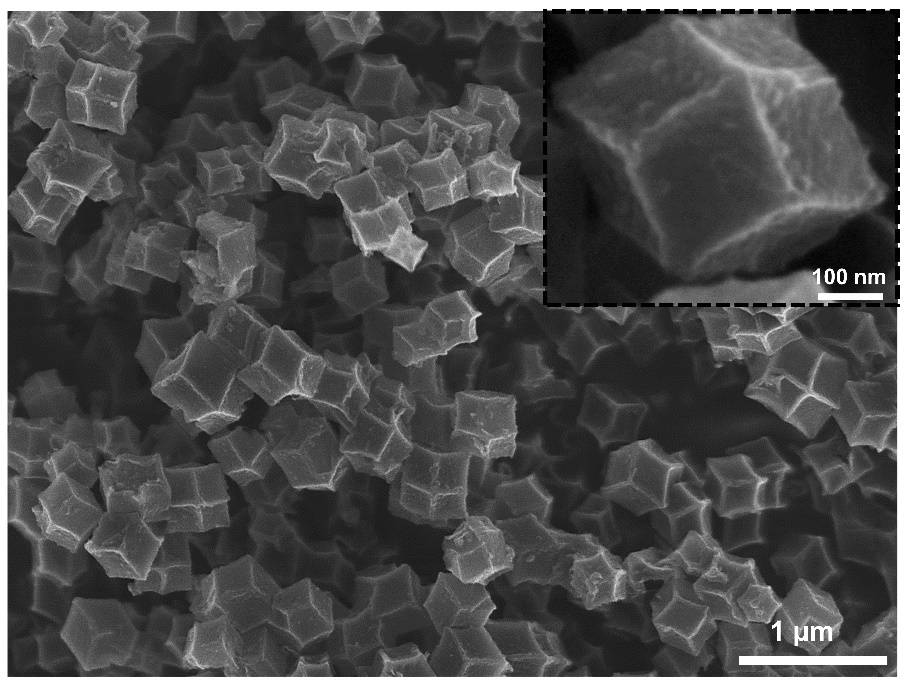


**Fig. S3** SEM image of FeMn-N-C pyrolyzed at 900 ℃

**
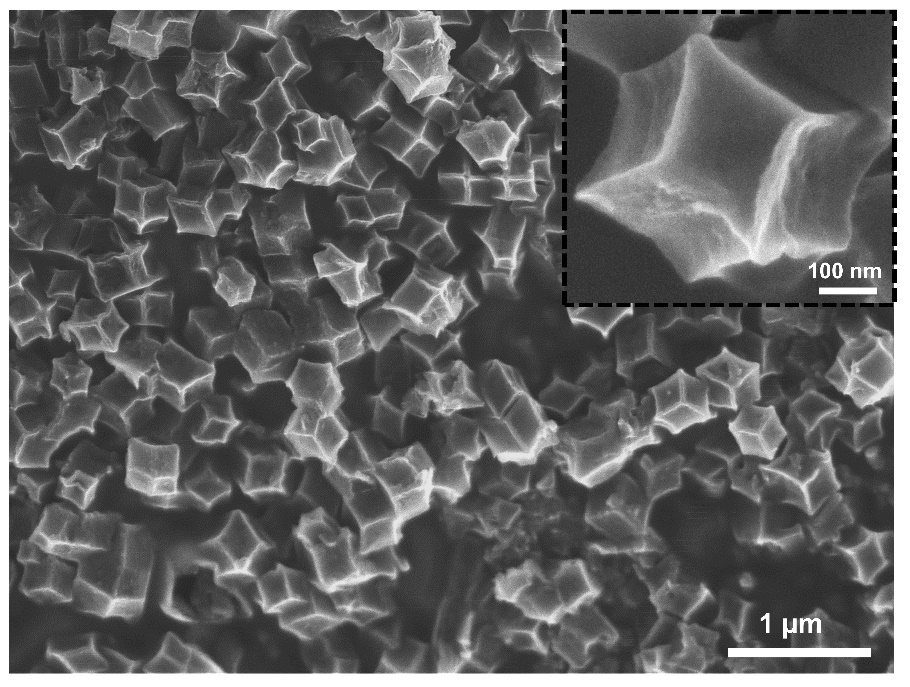
**

**Fig. S4** SEM image of FeMn-N-C pyrolyzed at 950 ℃


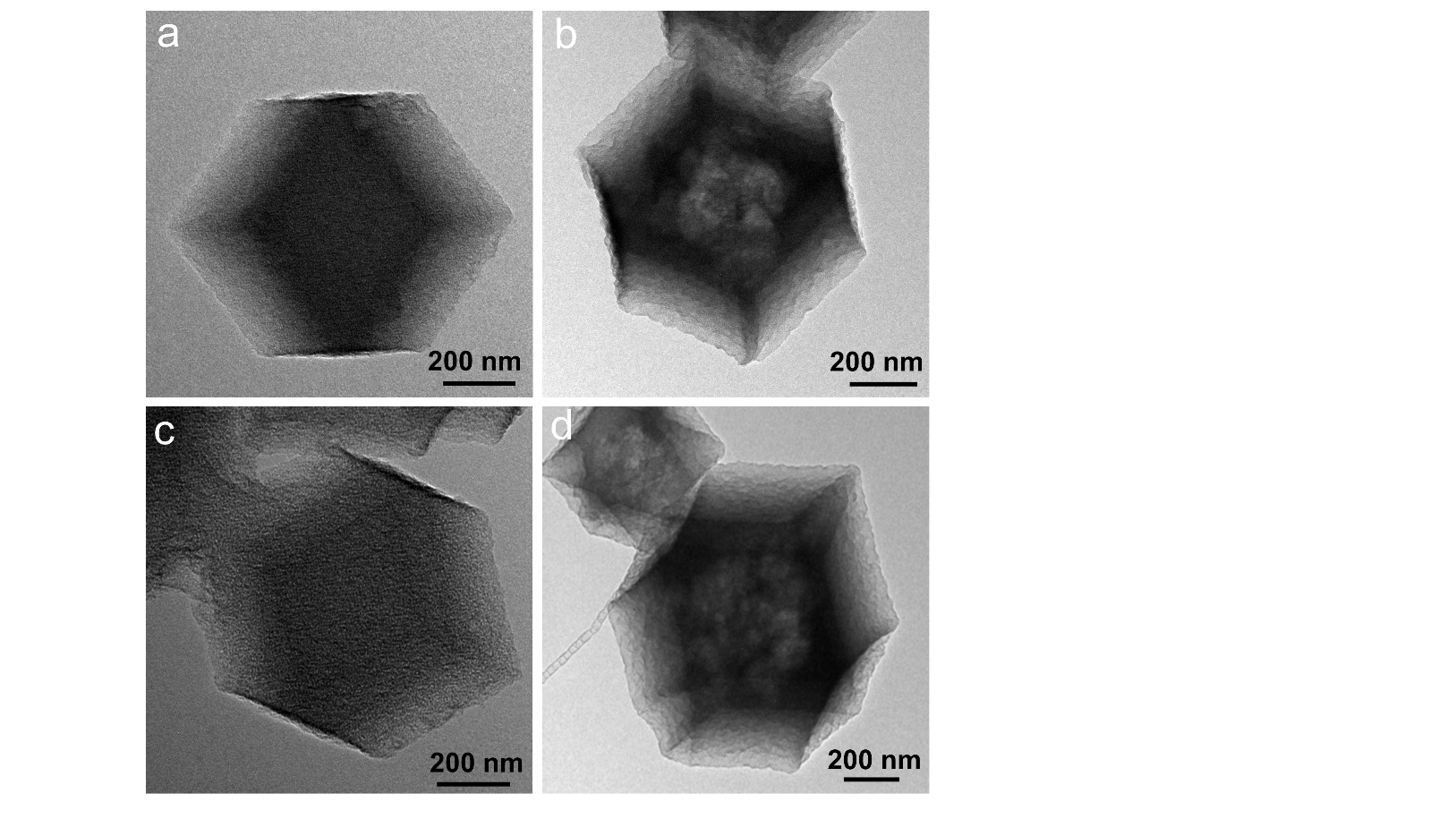


**Fig. S5** TEM image of **a** FeCl_3_·6H_2_O-(Fe-SA)-N-C, **b** (Fe-SA)-N-C, **c** FeCl_3_·6H_2_O-(FeMn-DA)-N-C, **d** (FeMn-DA)-N-C


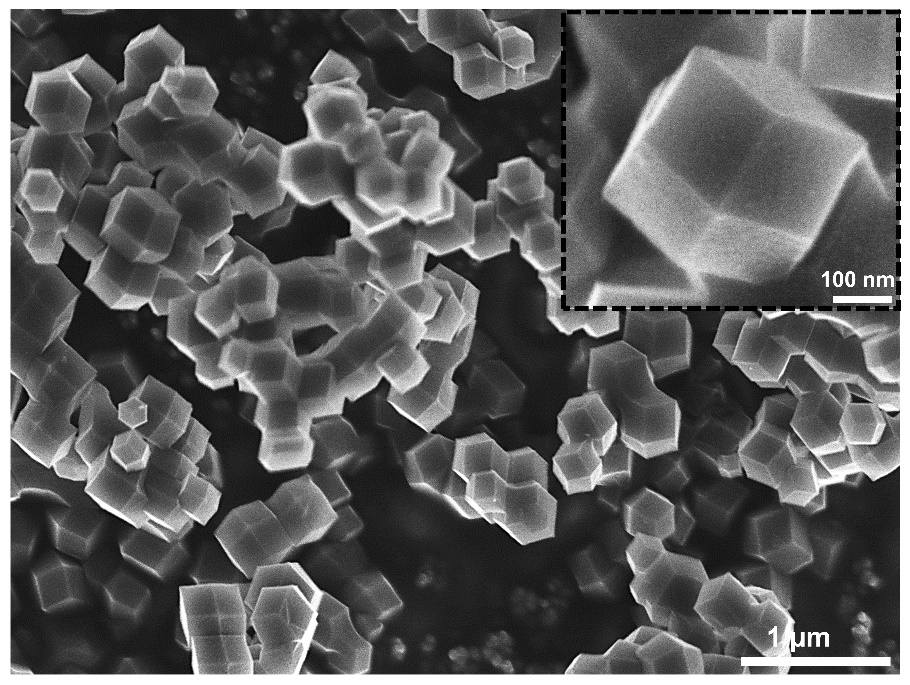


**Fig. S6** SEM image of (Mn-SA)-N-C


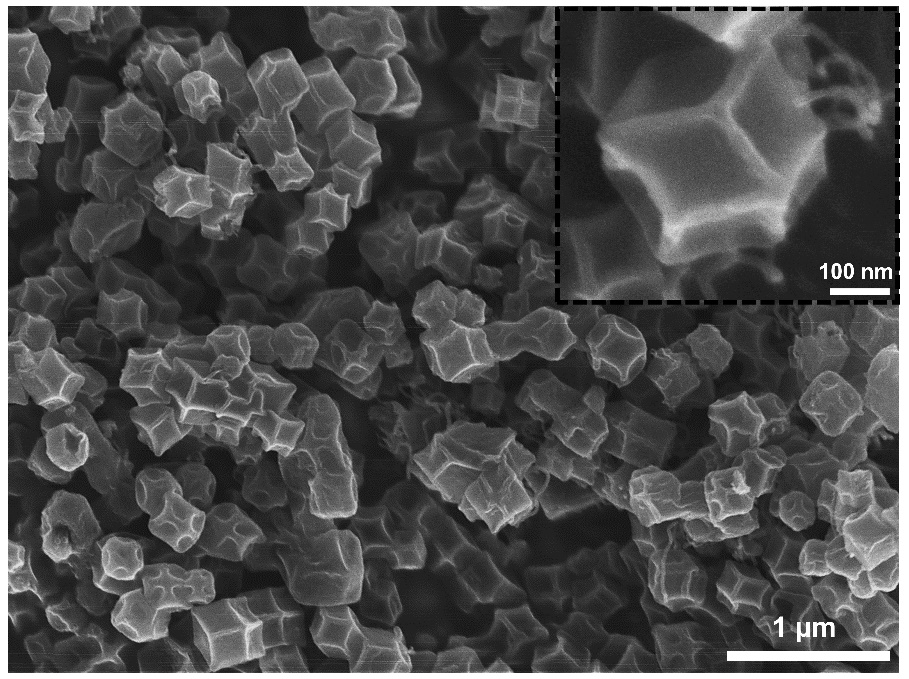


**Fig. S7** SEM image of (Fe-SA)-N-C


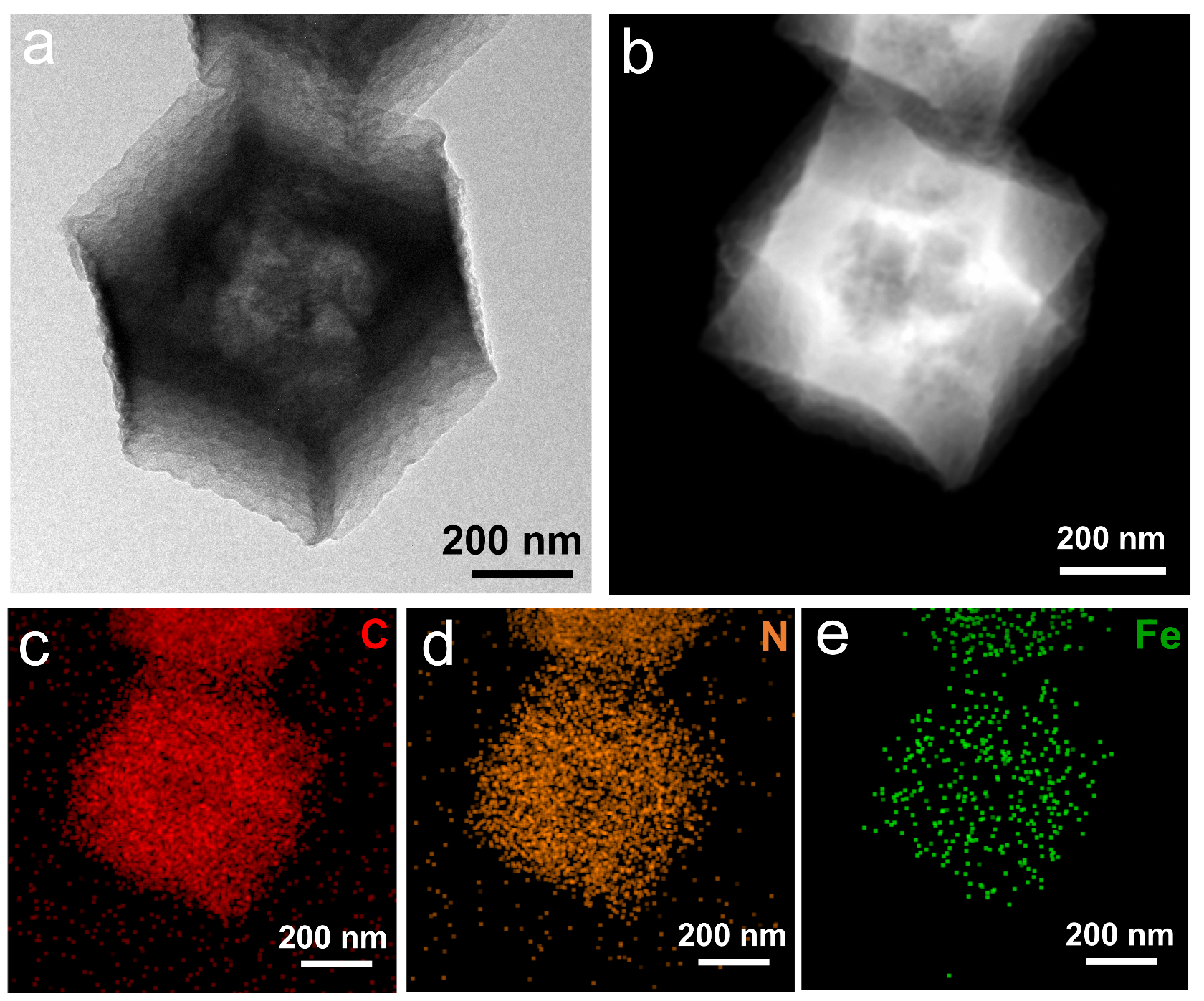


**Fig. S8** **a** TEM image, and **b-e** EDS mapping of (Fe-SA)-N-C


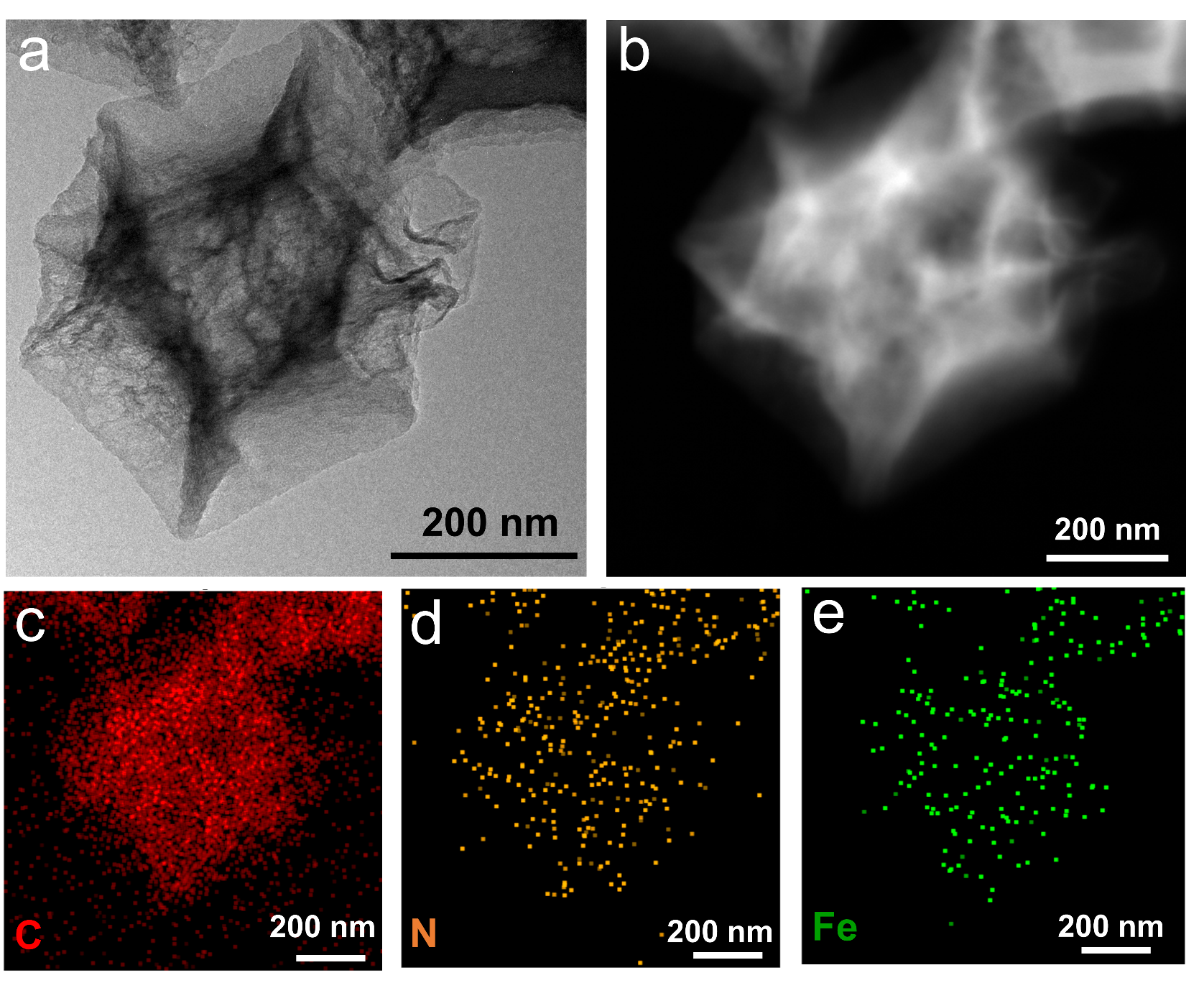


**Fig. S9 a** TEM image, and **b-e** EDS mapping of (Fe-SA)-N-C after 10k th CV cycles in 0.1 M KOH


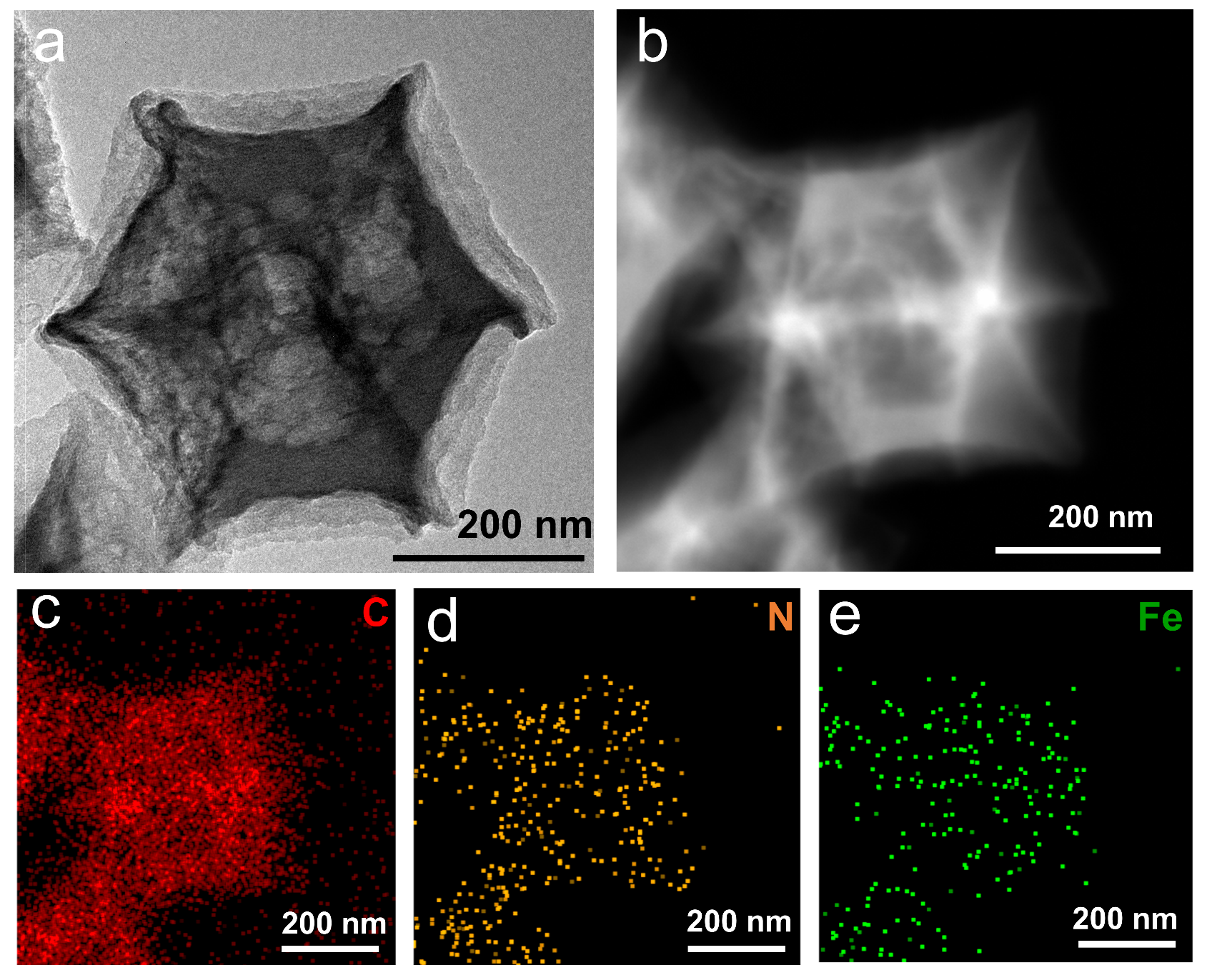


**Fig. S10 a** TEM image, and **b-e** EDS mapping of (Fe-SA)-N-C after 5k th CV cycles in 0.1 M HClO_4_


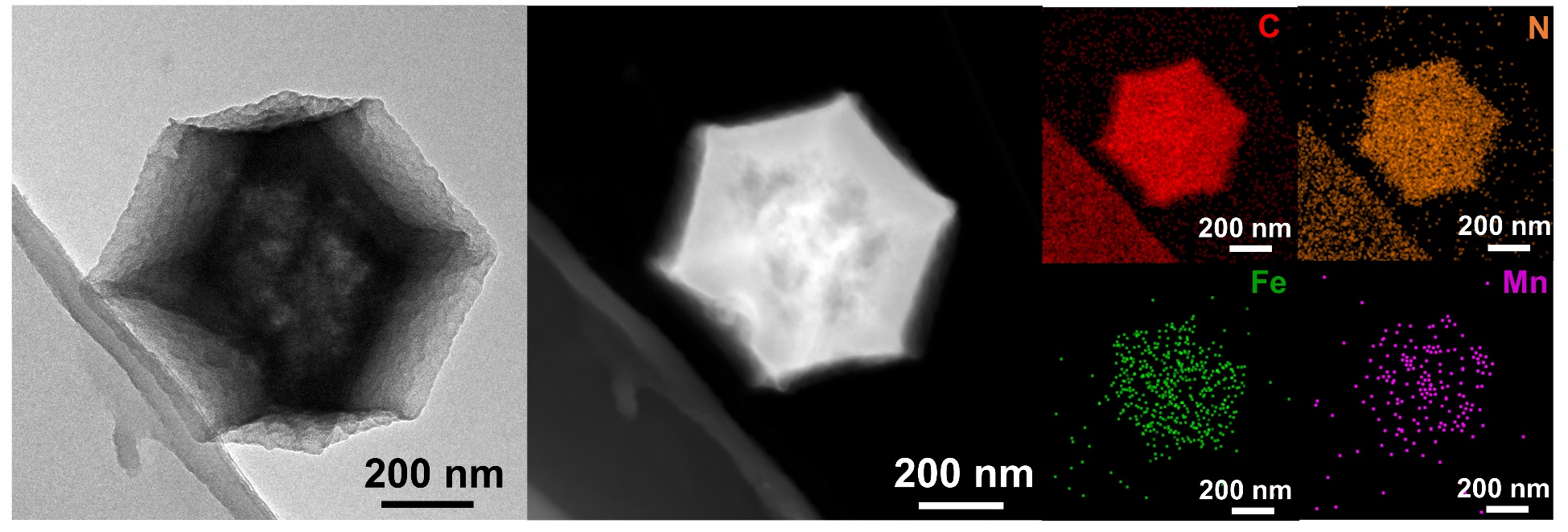


**Fig. S11 a** TEM image, and **b-e** EDS mapping of (FeMn-DA)-N-C after 10k th CV cycles in 0.1 M KOH


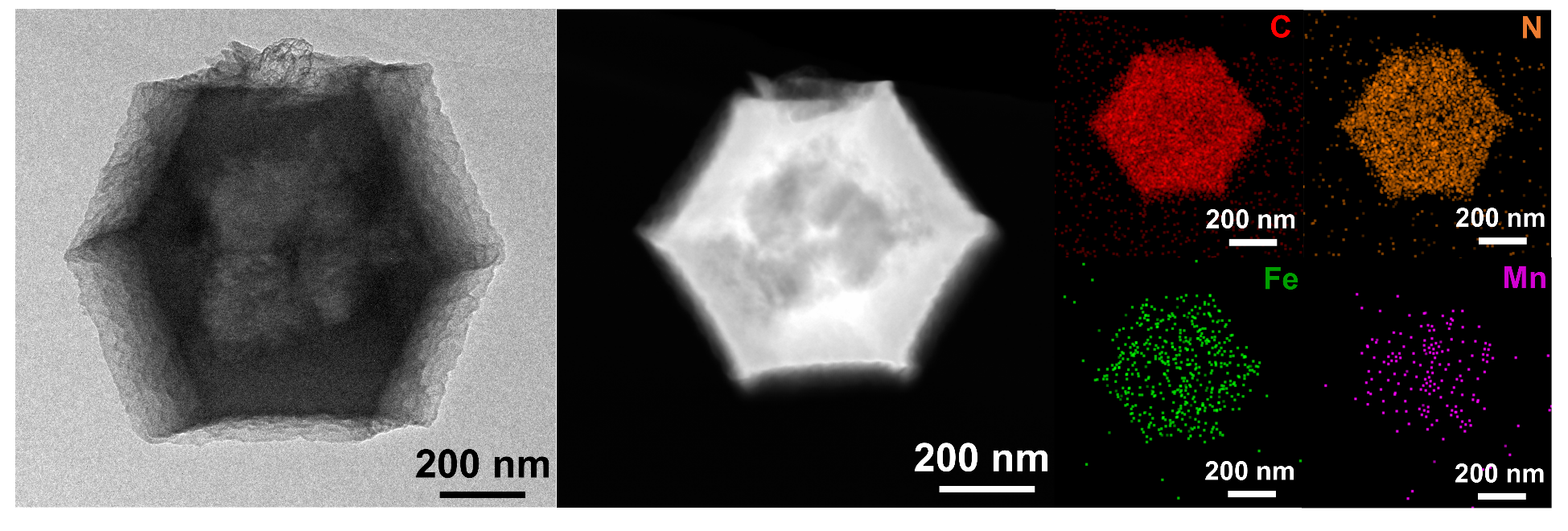


**Fig. S12 a** TEM image, and **b-e** EDS mapping of (FeMn-DA)-N-C after 5k th CV cycles in 0.1 M HClO_4_


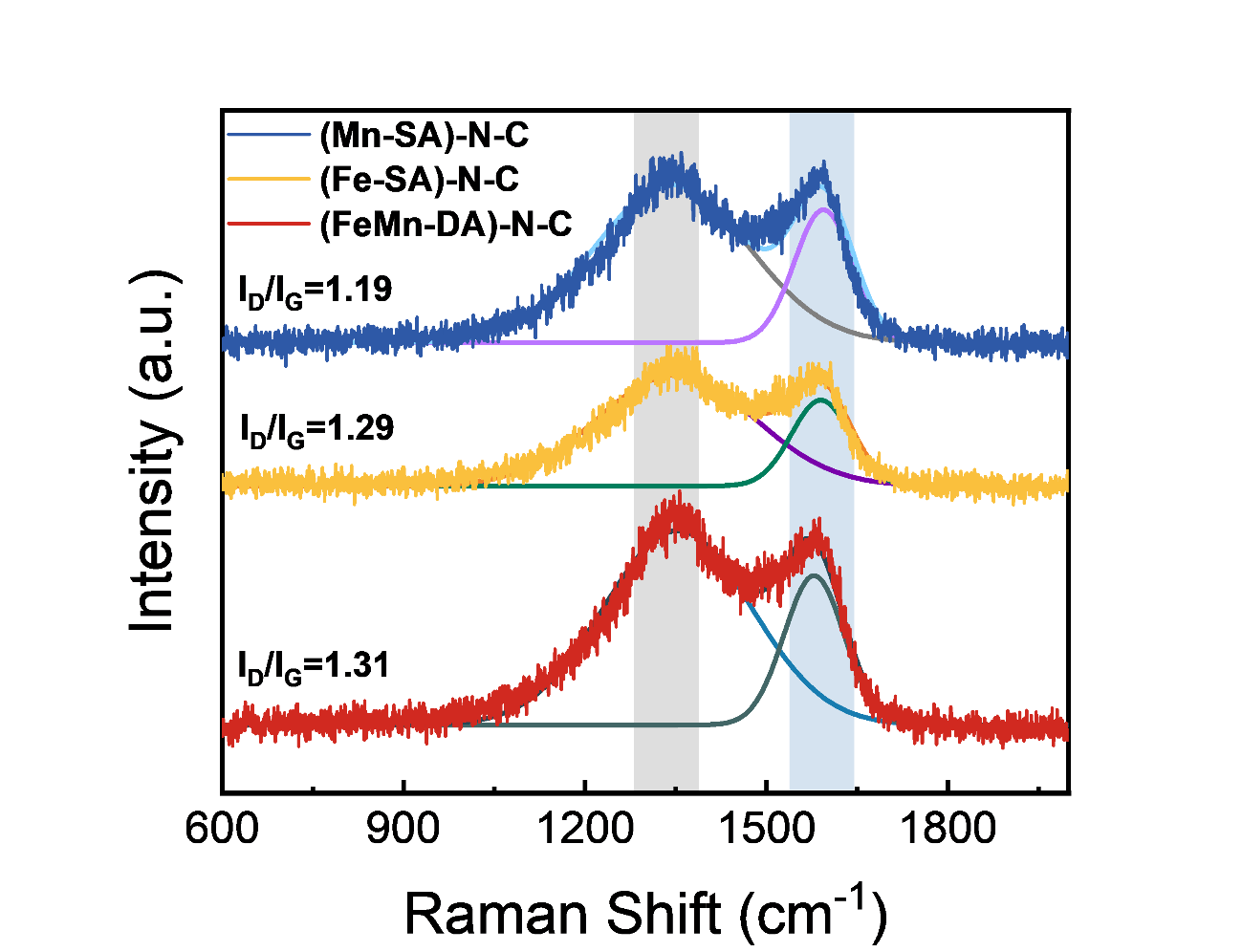


**Fig. S13** Raman spectra of (Mn-SA)-N-C, (Fe-SA)-N-C, and (FeMn-DA)-N-C


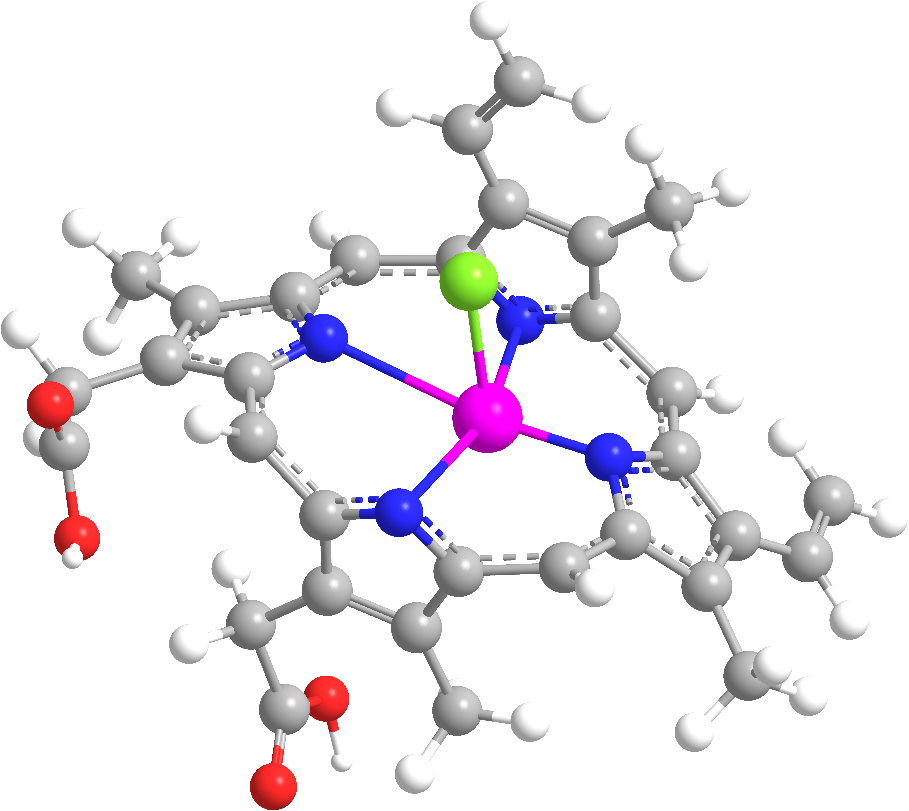


**Fig. S14** Molecular structure of hemin (15.1×15.3×10.6 Å)


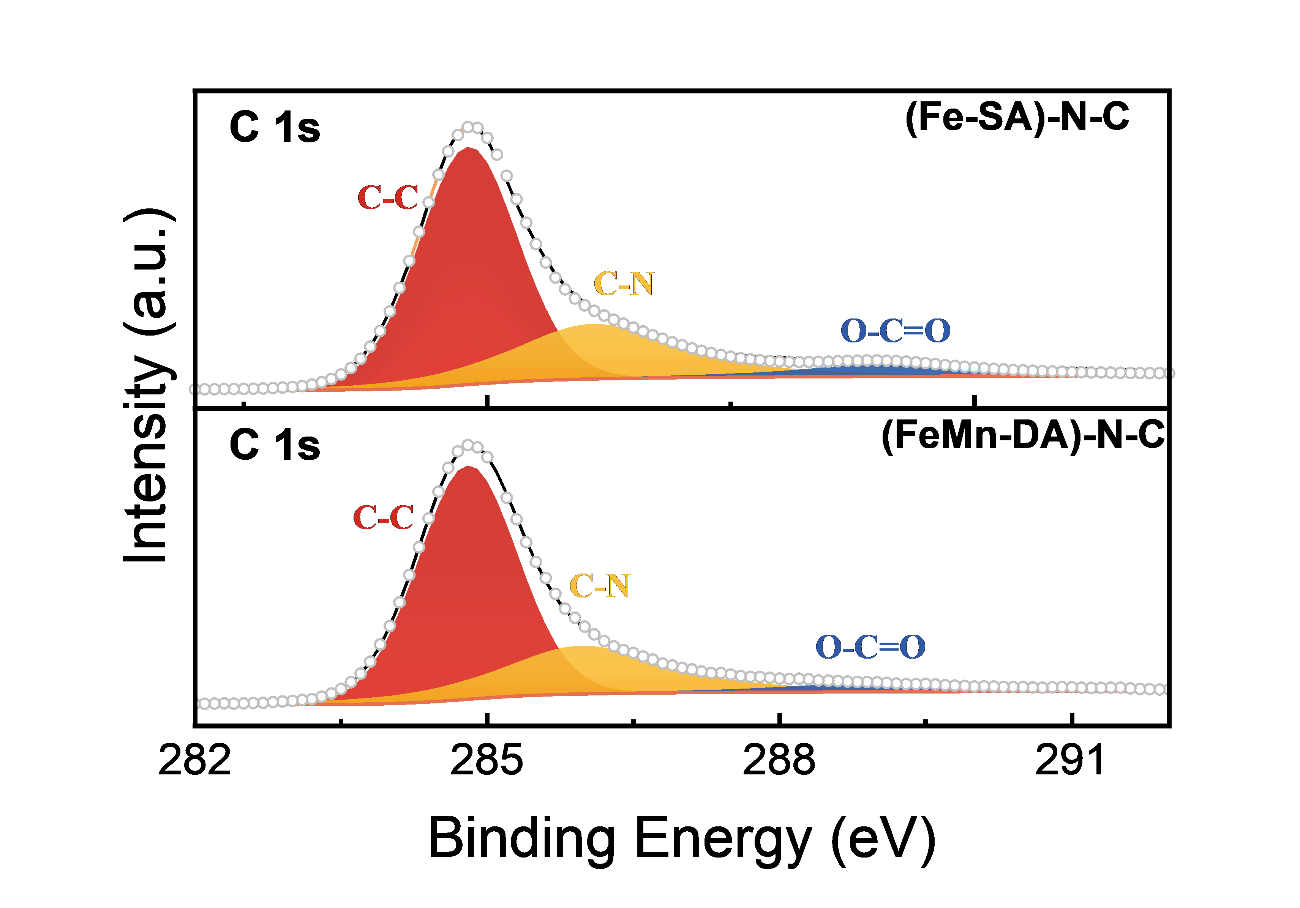


**Fig. S15** XPS spectra of C 1s for (Fe-SA)-N-C and (FeMn-DA)-N-C


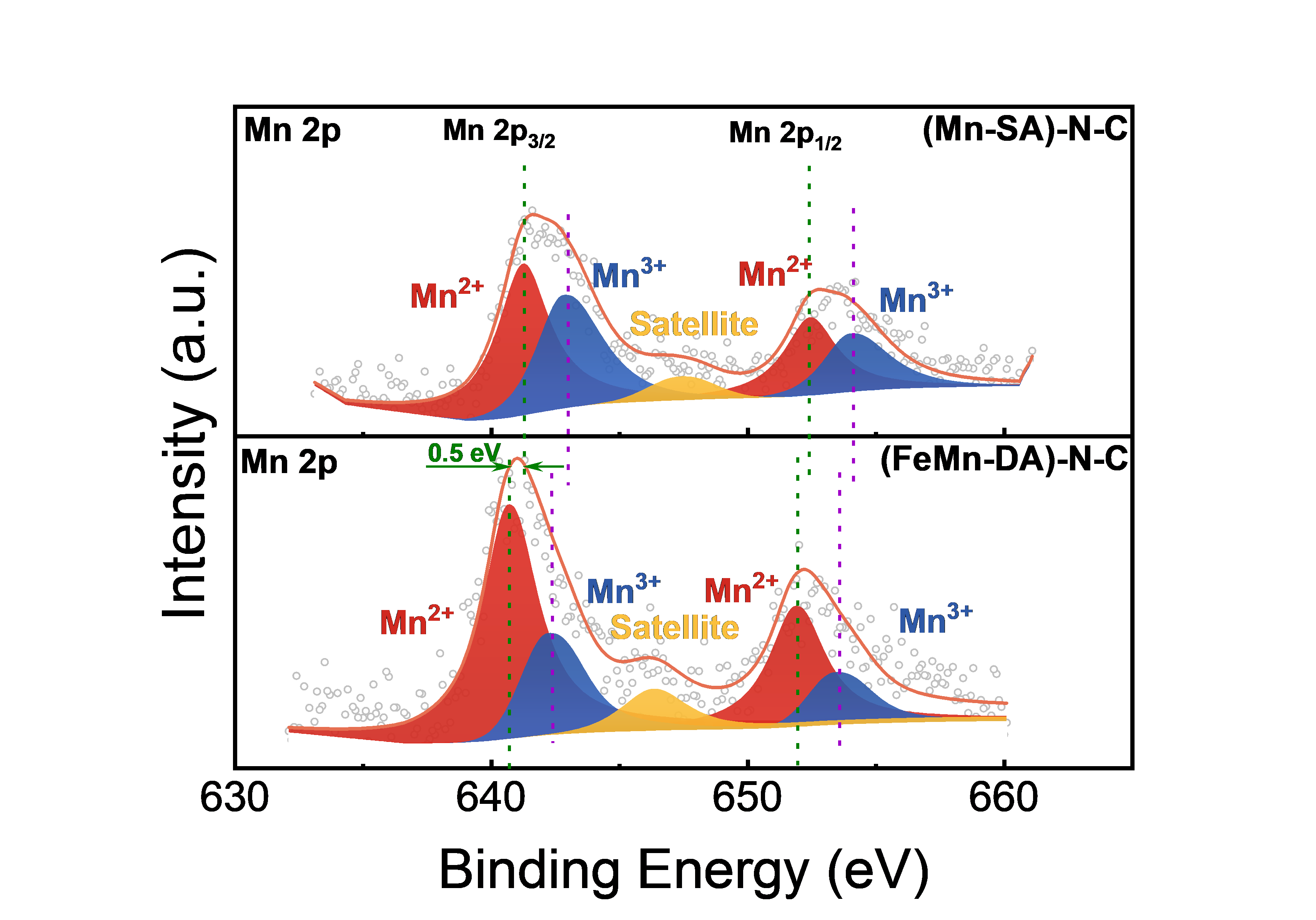


**Fig. S16** XPS spectra of Mn 2p for (Mn-SA)-N-C and (FeMn-DA)-N-C


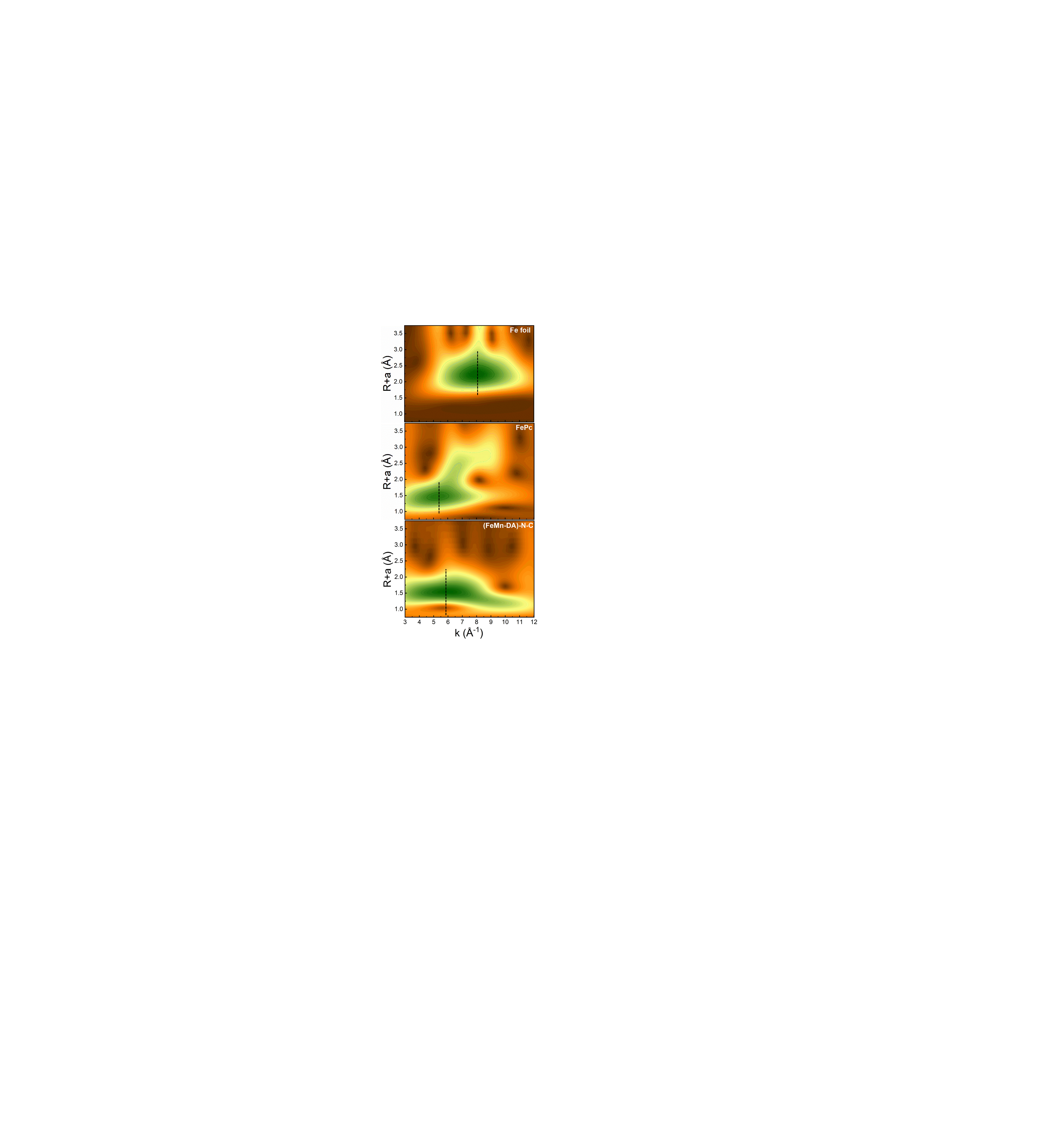


**Fig. S17** WT for the k^3^-weighted EXAFS signals at Fe Mn K-edge.


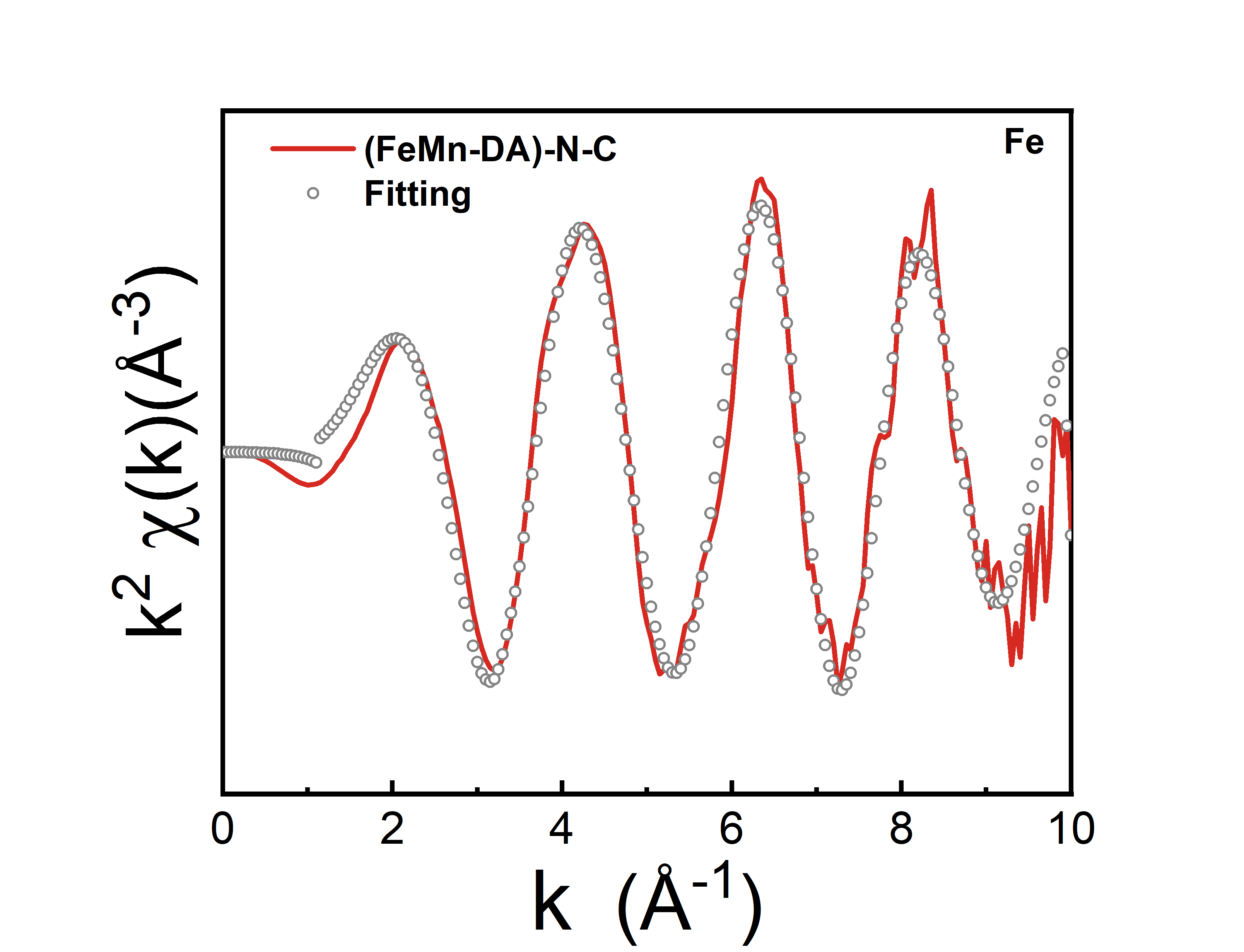


**Fig. S18** Fe K-edge EXAFS fitting curves of (FeMn-DA)-N-C


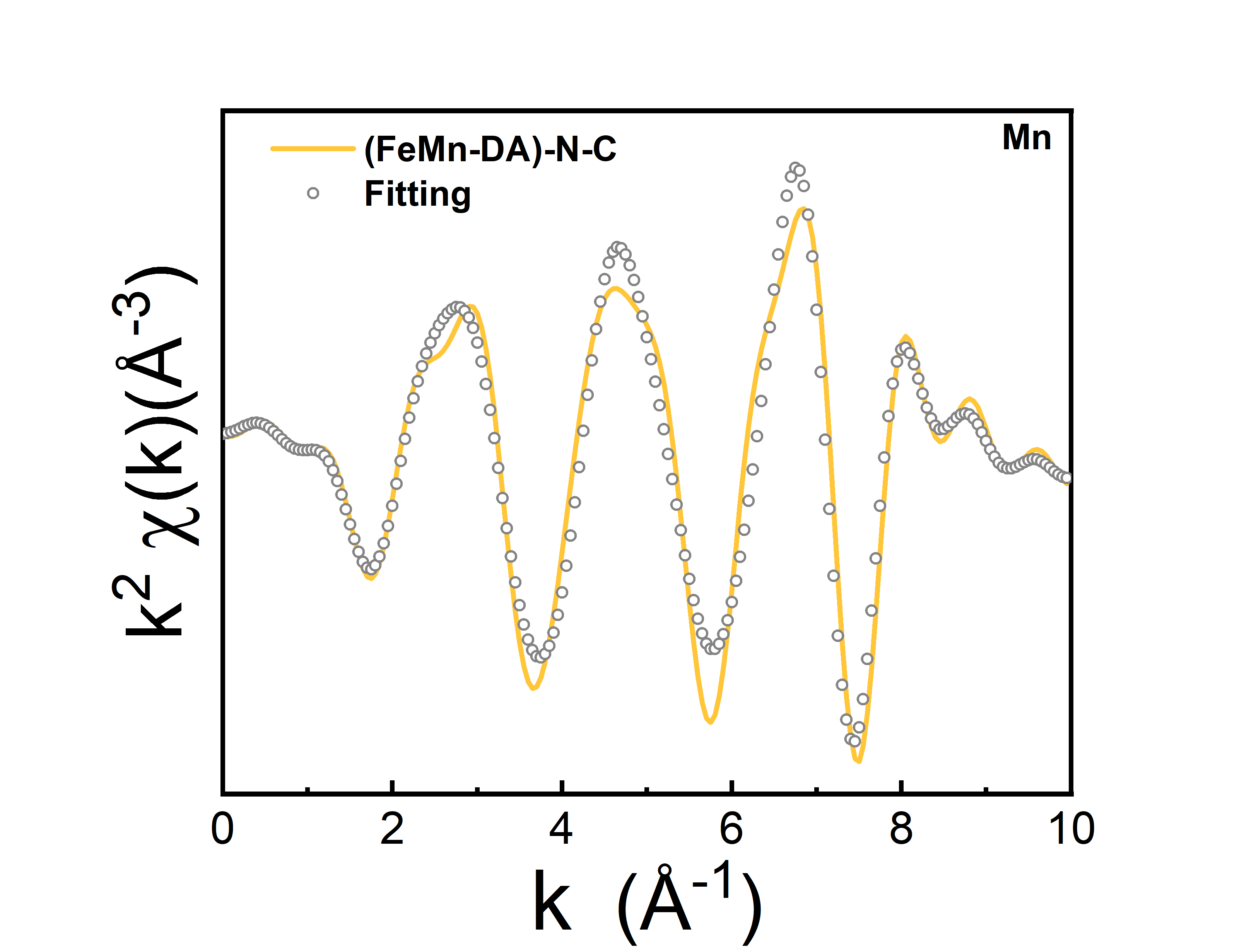


**Fig. S19** Mn K-edge EXAFS fitting curves of (FeMn-DA)-N-C





**Fig. S20** CV curves of FeMn-N-C at 900 ℃, 920 ℃, and 950 ℃ in O_2_-saturated 0.1 M KOH





**Fig. S21** LSV curves of FeMn-N-C at 900 ℃, 920 ℃, and 950 ℃ in O_2_-saturated 0.1 M KOH





**Fig. S22** Tafel slope of FeMn-N-C at 900 ℃, 920 ℃, and 950 ℃ in O_2_-saturated 0.1 M KOH





**Fig. S23** CV curves of FeMn-N-C at 900 ℃, 920 ℃, and 950 ℃ in O_2_-saturated 0.1 M HClO_4_





**Fig. S24** LSV curves of FeMn-N-C at 900 ℃, 920 ℃, and 950 ℃ in O_2_-saturated 0.1 M HClO_4_





**Fig. S25** Tafel slope of FeMn-N-C at 900 ℃, 920 ℃, and 950 ℃ in O_2_-saturated 0.1 M HClO_4_





**Fig. S26** LSV curves of (Fe-SA)-N-C catalysts with different Fe additions in **a** 0.1 M KOH and **b** 0.1 M HClO_4_





**Fig. S27** LSV curves of (FeMn-DA)-N-C catalysts with different Mn additions in **a** 0.1 M KOH and **b** 0.1 M HClO_4_.





**Fig. S28** Polarization curves of the different Fe sources in **a** 0.1 M KOH and **b** 0.1 M HClO_4_


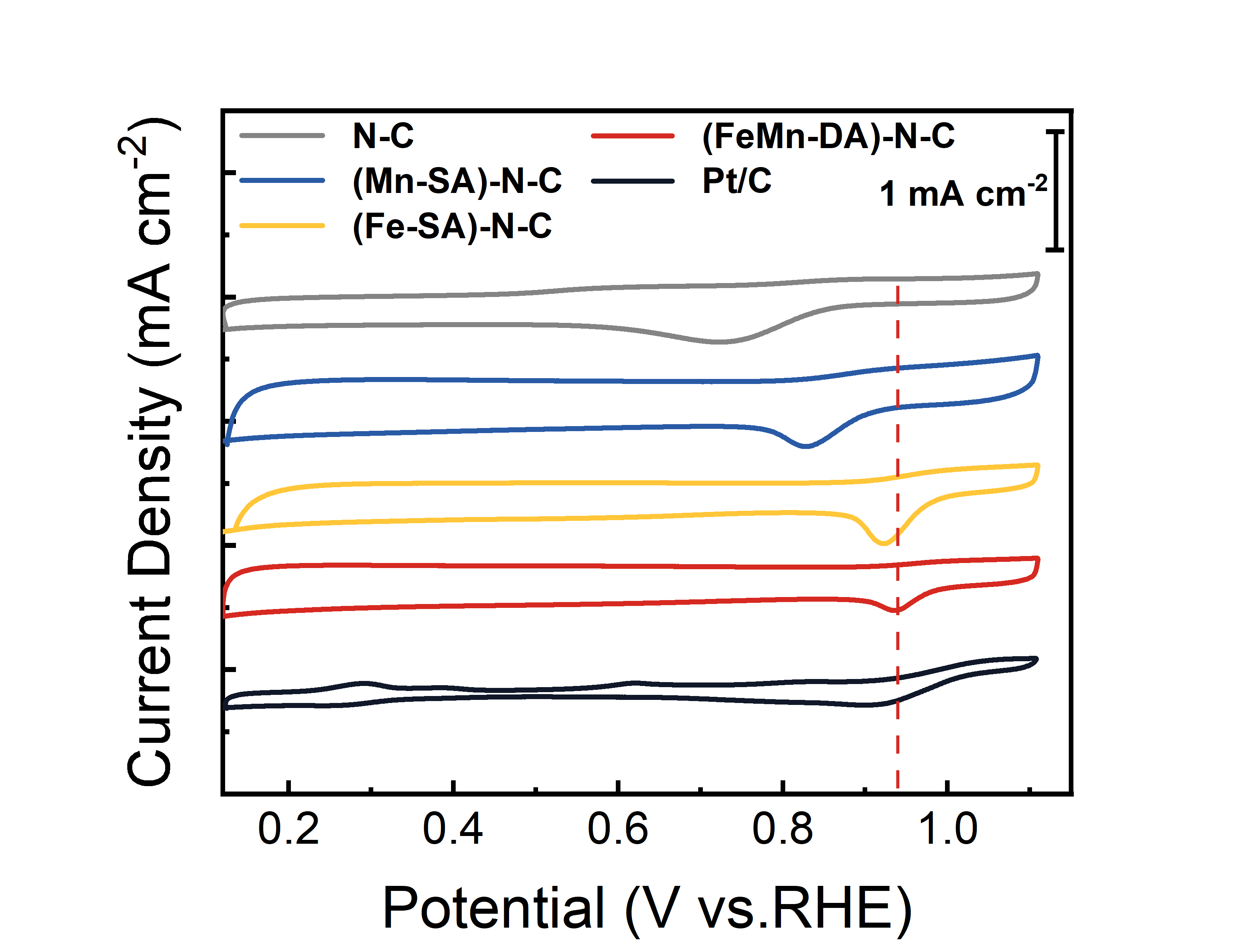


**Fig. S29** CV curves of catalysts in O_2_-saturated 0.1 M KOH


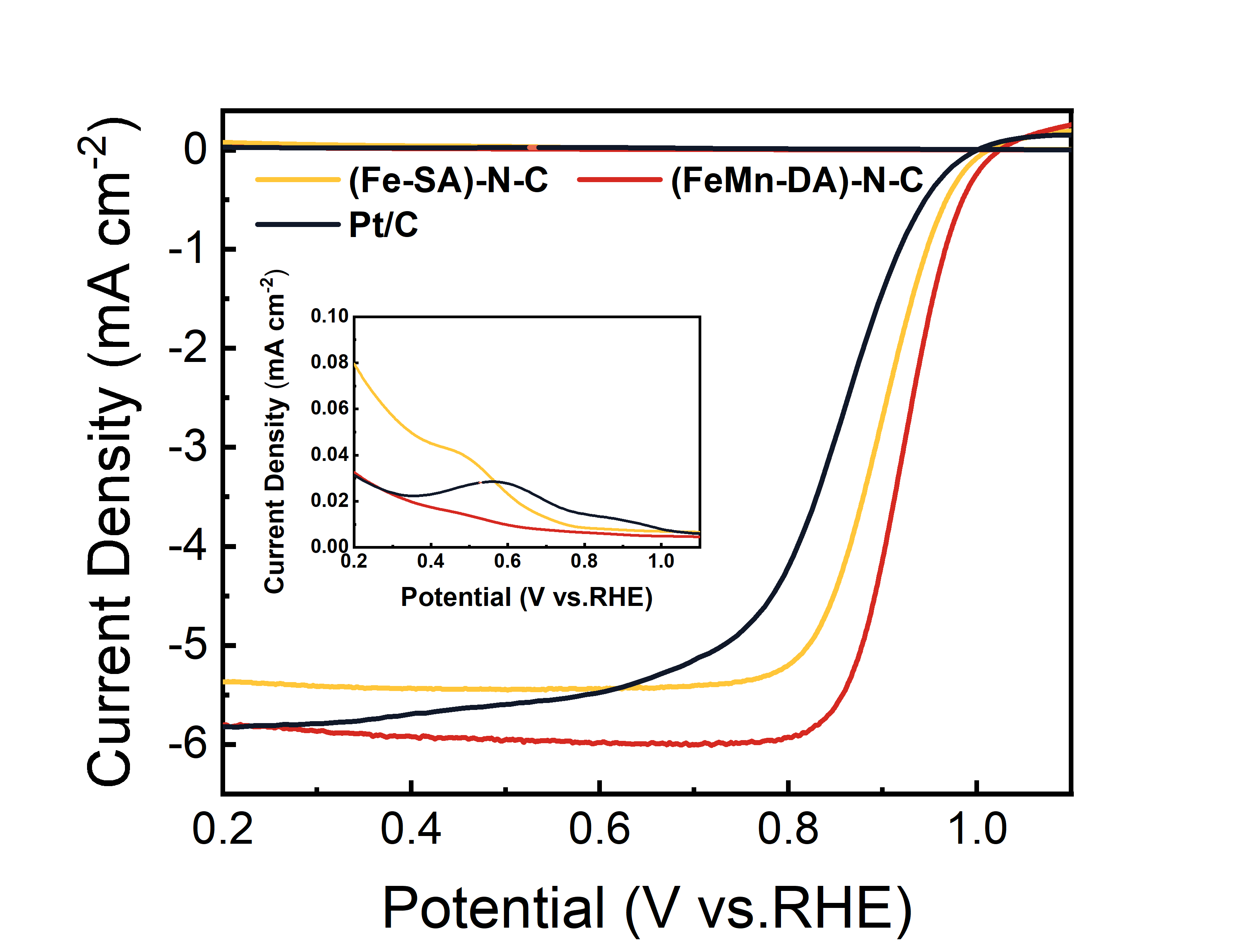


**Fig. S30** RRDE-LSV curves of catalysts at 1600 rpm in 0.1 M KOH


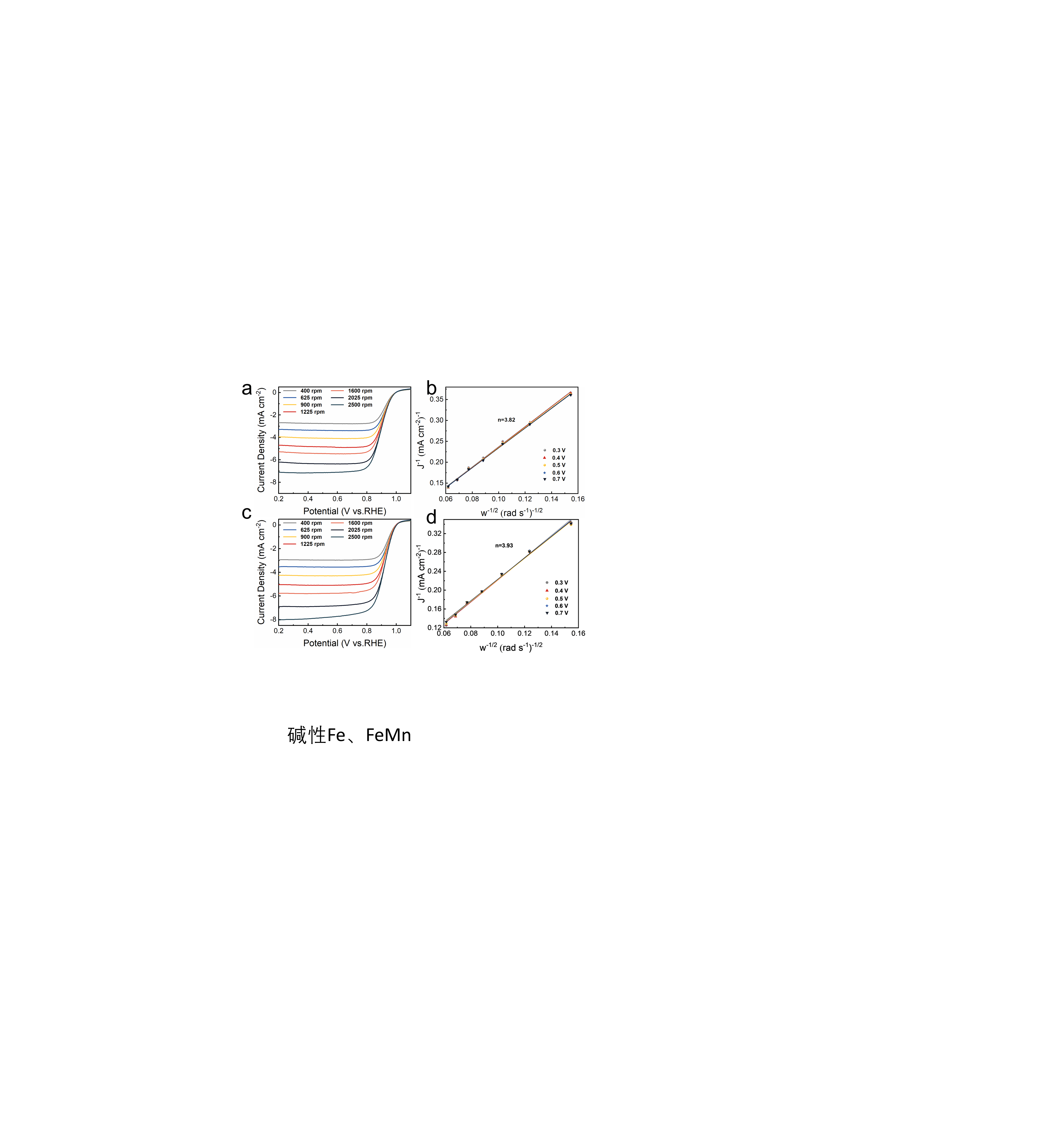


**Fig. S31** LSV curves at different rotating rates and Koutecky-Levich (K-L) plots of **a, b** (Fe-SA)-N-C, and **c,d** (FeMn-DA)-N-C catalysts in O_2_ saturated 0.1 M KOH


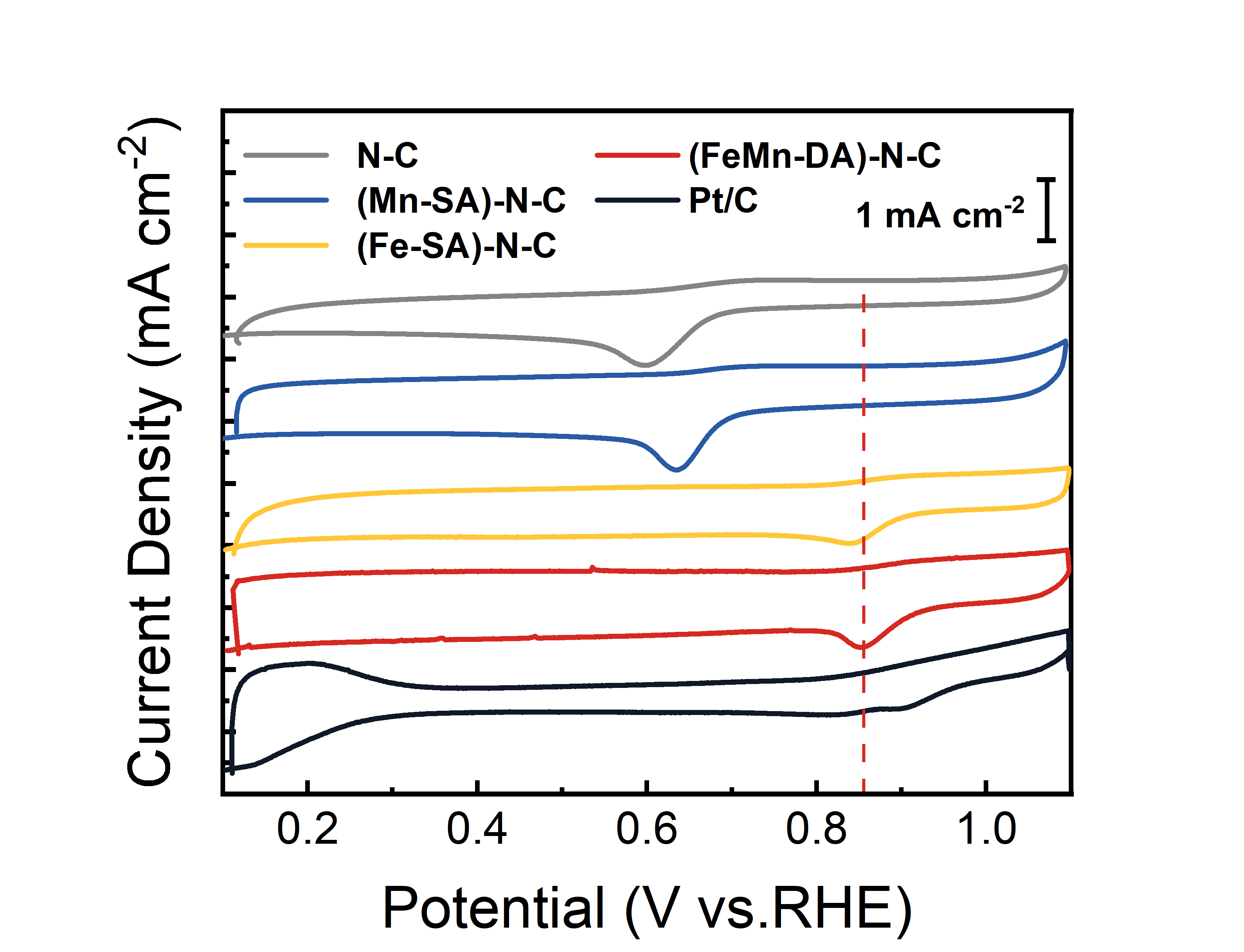


**Fig. S32** CV curves of catalysts in O_2_-saturated 0.1 M HClO_4_


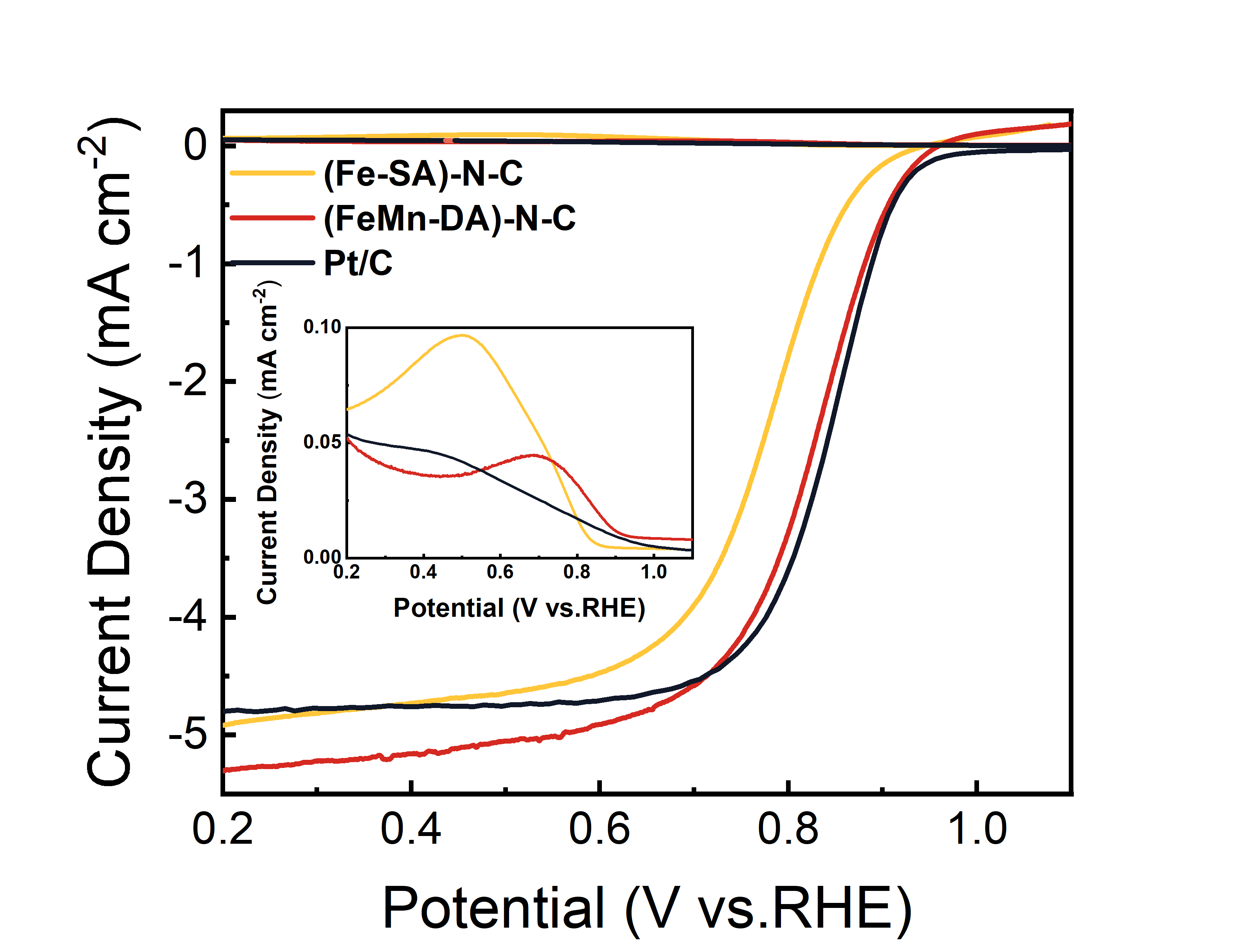


**Fig. S33** RRDE-LSV curves of catalysts at 1600 rpm in 0.1 M HClO_4_


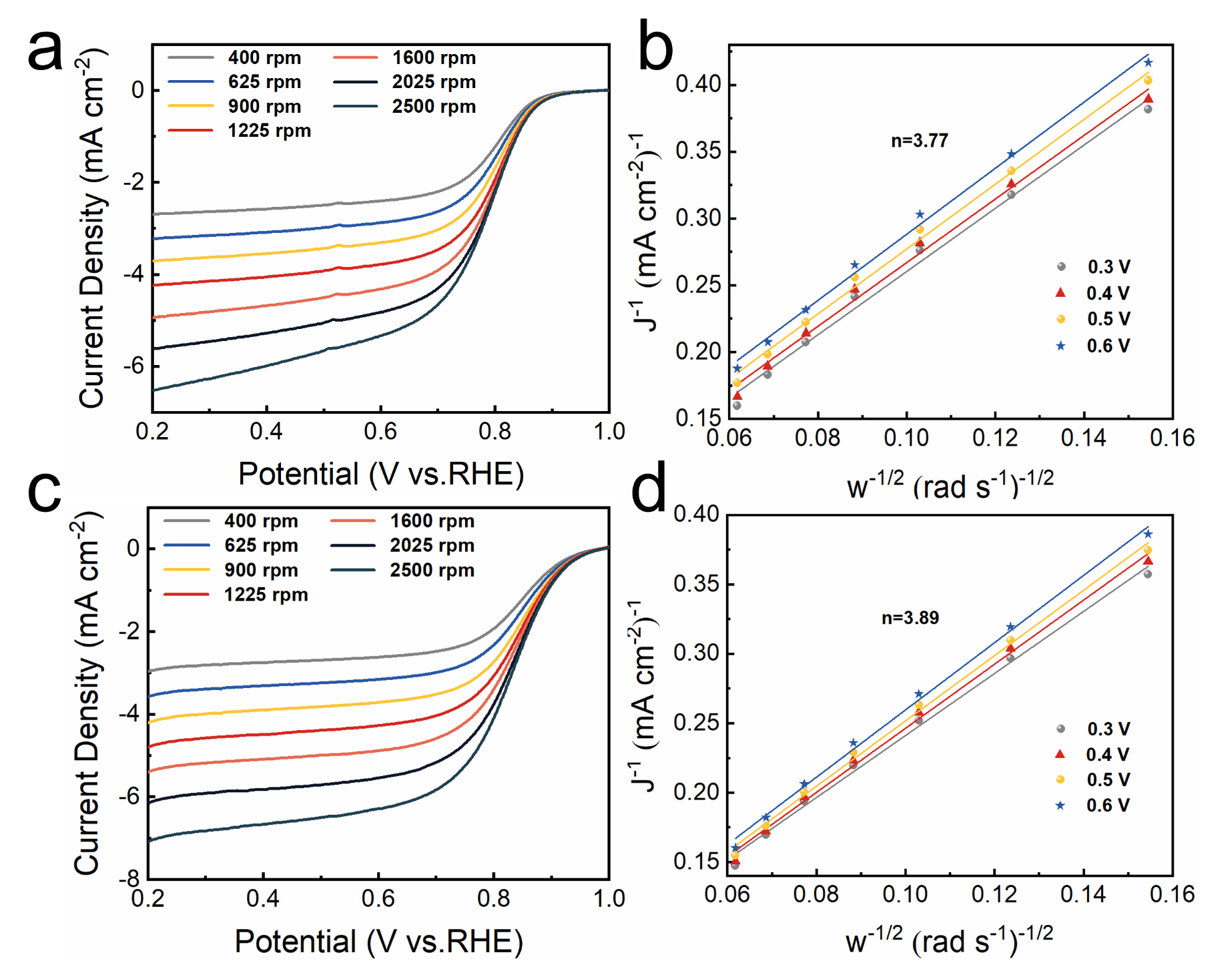


**Fig. S34** LSV curves at different rotating rates and Koutecky-Levich (K-L) plots of **a, b** (Fe-SA)-N-C, and **c, d** (FeMn-DA)-N-C catalysts in O_2_ saturated 0.1 M HClO_4_


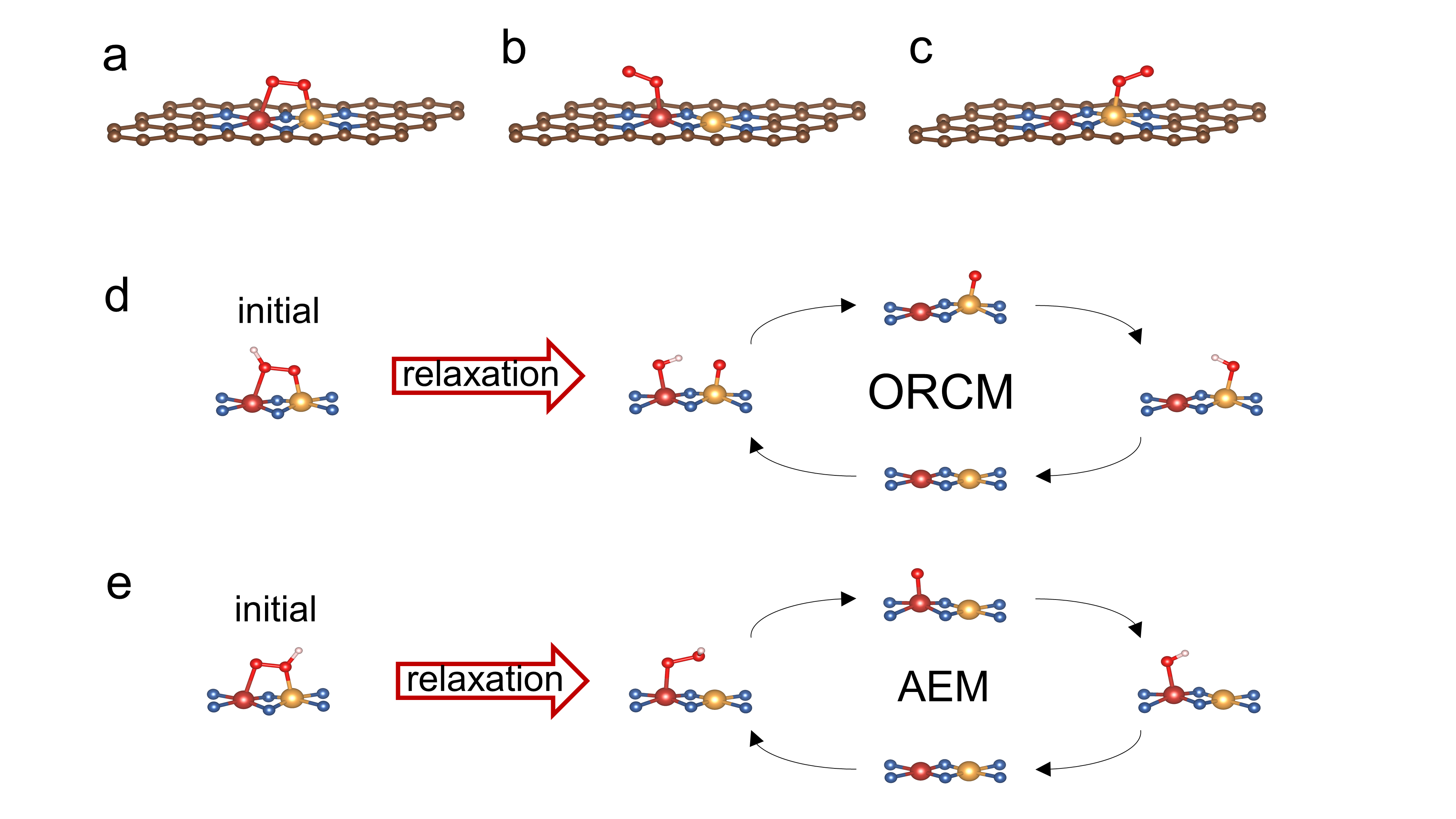


**Fig. S35** **a** FeMn parallel adsorption configuration of O_2_. The perpendicular adsorption configuration on **b** Fe and **c** Mn. The initial structure of proton adsorbing on **d** O_Fe_ and **e** O_Mn_, and the corresponding ORR mechanism


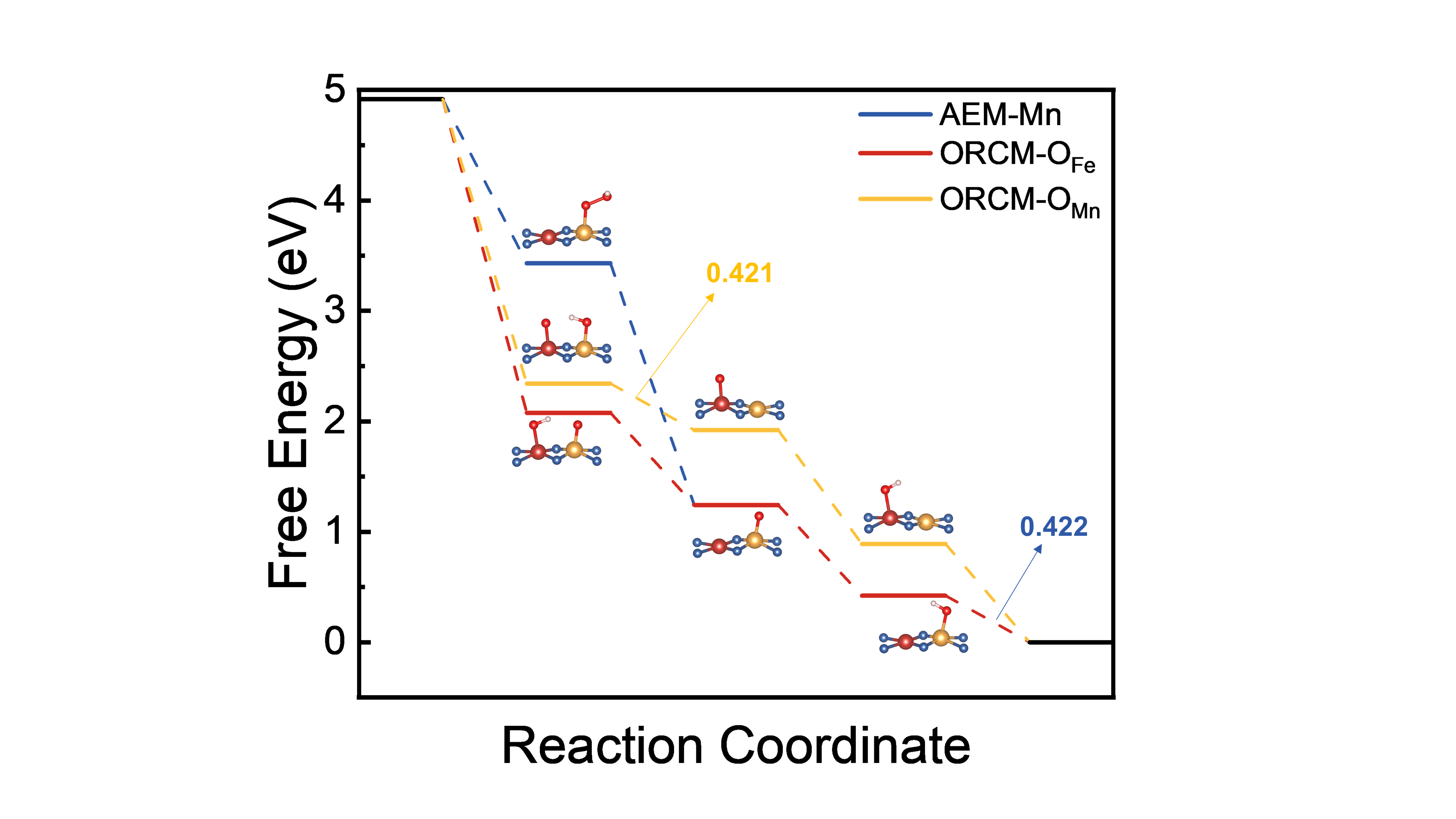


**Fig. S36** The free energy diagram of reverse AEM on the Mn site, and the reverse ORCM initiated by the O_Mn_ or the O_Fe_ site





**Fig. S37** Potential-dependent in-situ Raman spectra during ORR in, **a** alkaline and **b** acidic solutions.

In-situ Raman testing was employed to investigate the real-time response of the catalyst during ORR, providing insights into the transformation of intermediates under both alkaline and acidic conditions. As shown in **Fig. S37a**, the in-situ Raman spectroscopy results show that no significant Raman signals were observed above 0.8 V in alkaline solutions. However, a peak appeared around 730 cm⁻¹ as the potential decreased. According to electrochemical testing and theoretical calculations, this peak can be attributed to the OOH intermediate vibrational mode, a crucial intermediate in the ORR. In contrast, a prominent OH peak was observed in basic solutions, likely due to strong OH adsorption. In acidic conditions(**Fig. S37b**), OOH peaks also exist at about 730 cm, also a vibrational peak at 933 cm⁻¹ corresponds to the ClO₄ vibrations in the acidic solution [S11].


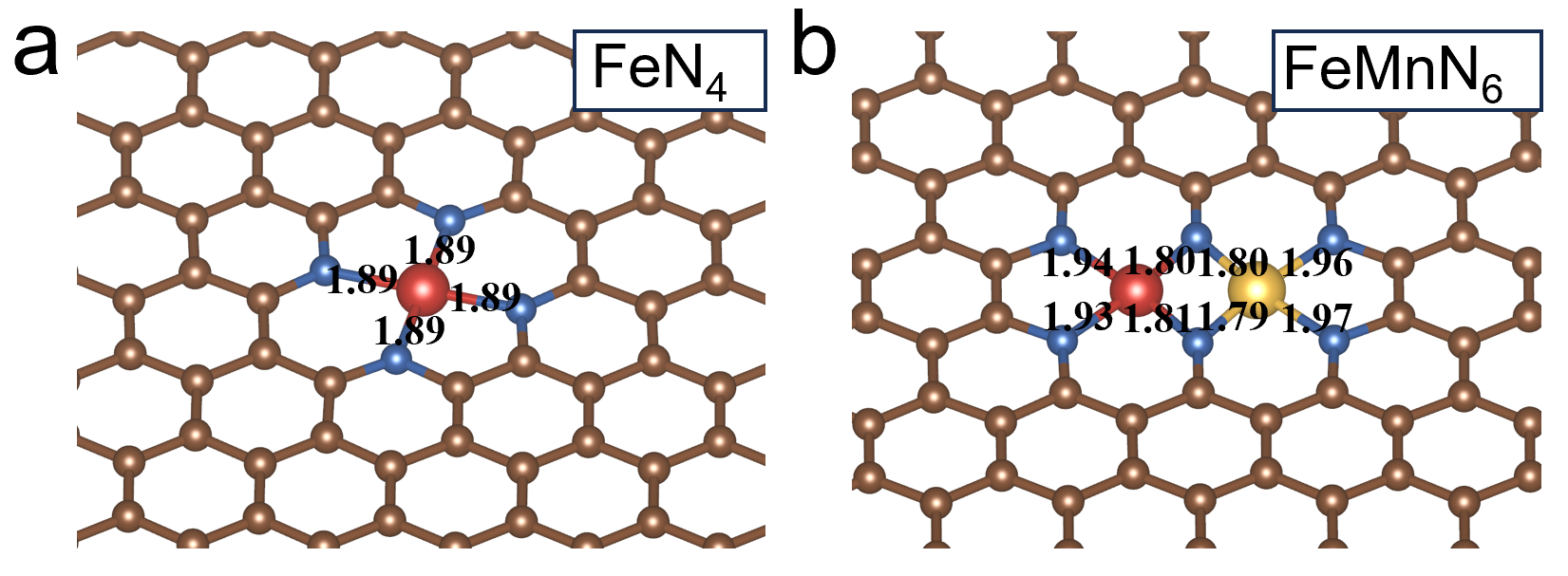


**Fig. S38** Optimized geometry structures of **a** FeN_4_, **b** FeMnN_6_. Length of M-N bonds are also labeled (unit: Å).


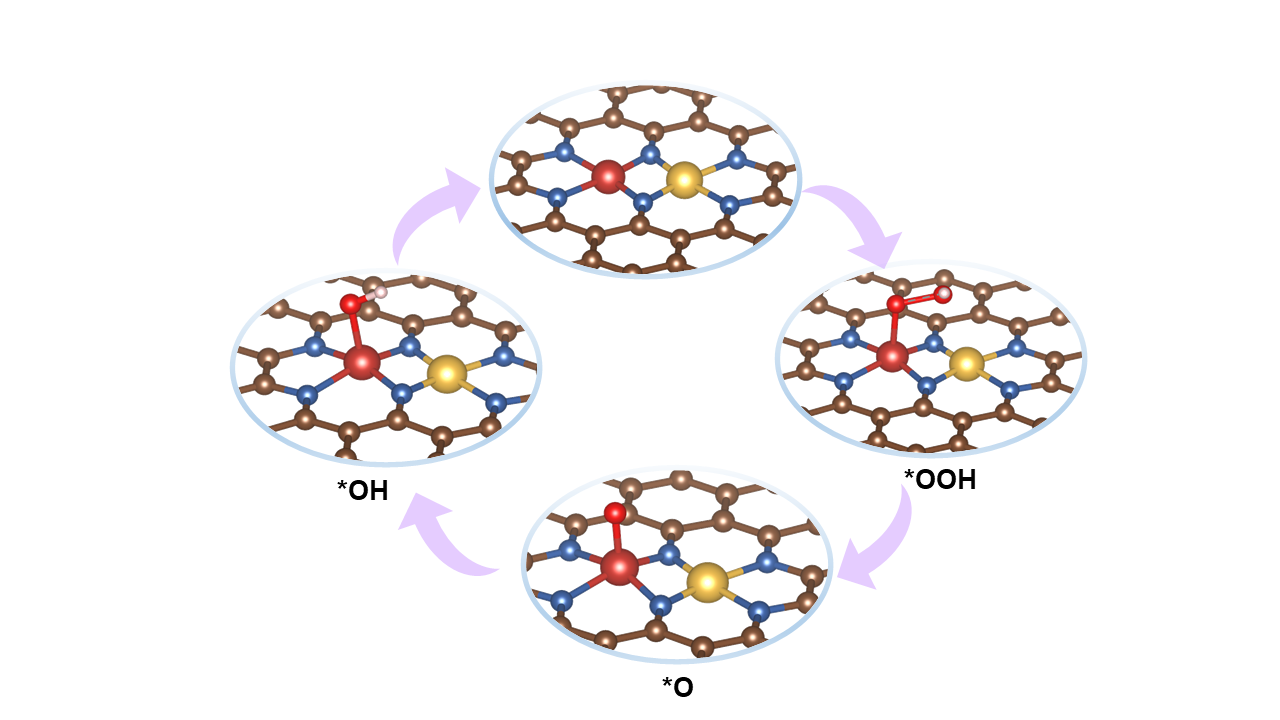


**Fig. S39** Illustrations of ORR process


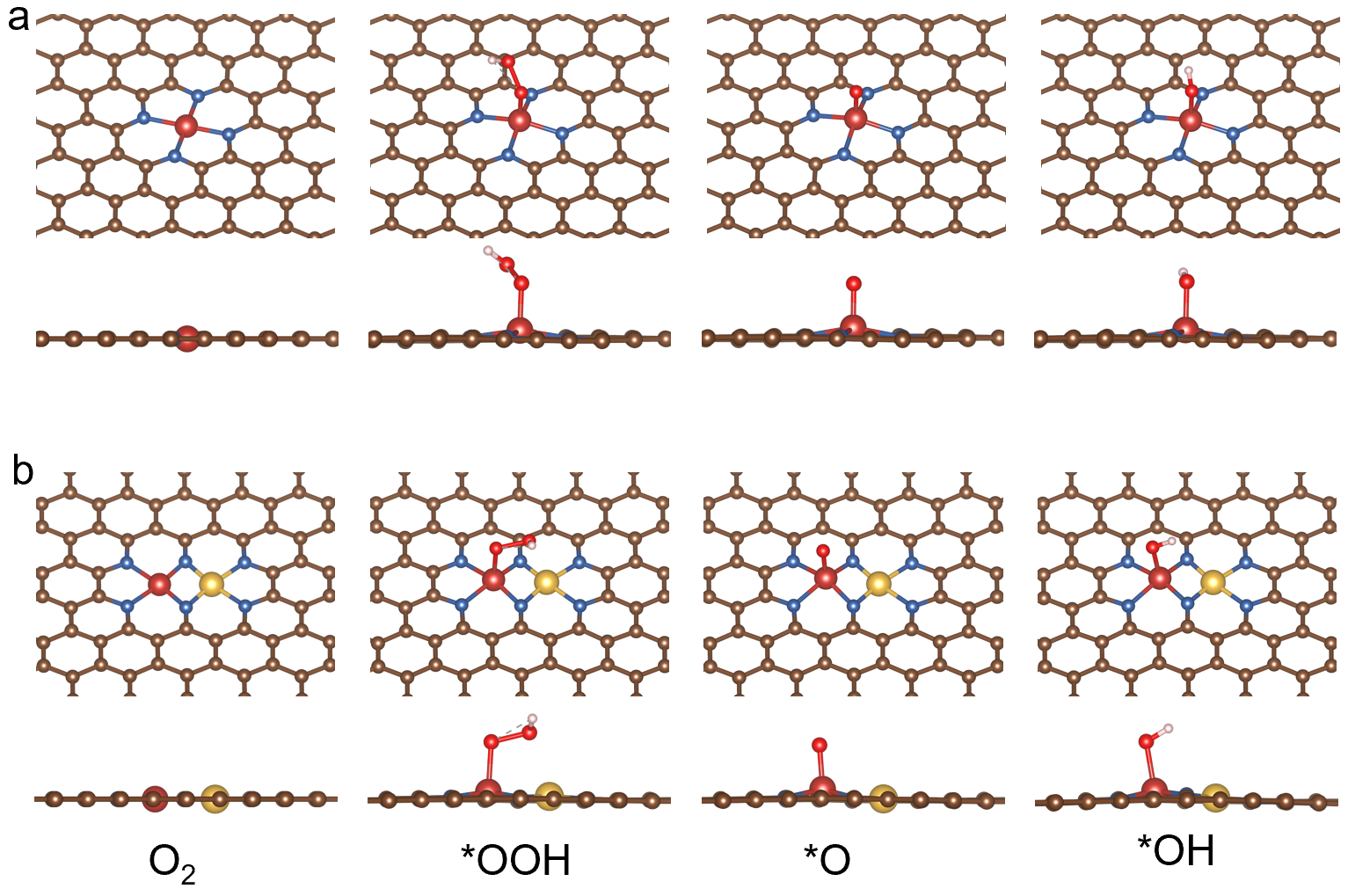


**Fig. S40** Geometrical structure of bare and intermediates adsorbed (i.e., *OOH, *O and *OH) surfaces of **a** FeN_4_ and **b** FeMnN_6_


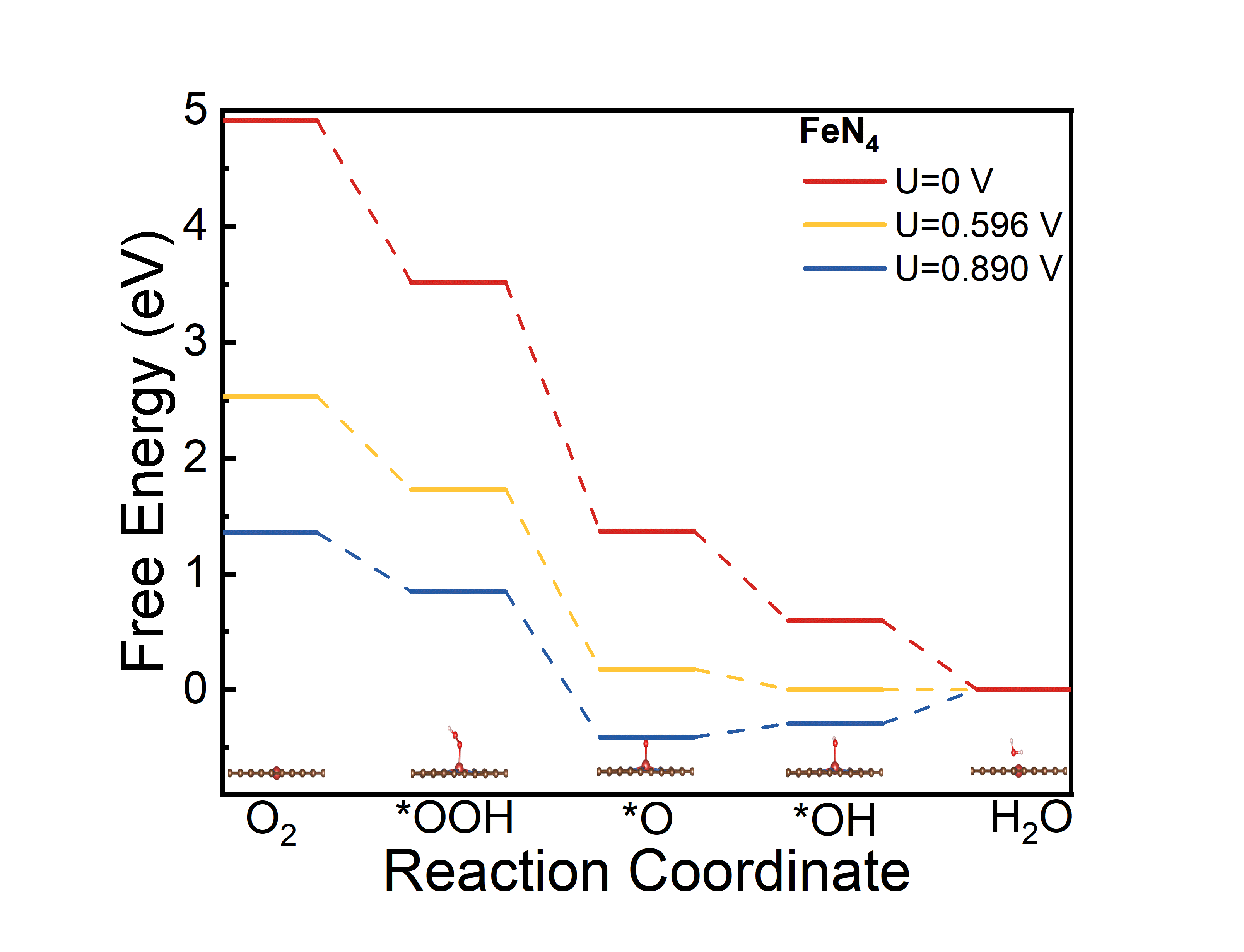


**Fig. S41** Gibbs free energy diagram for ORR of FeN_4_ at different U

**Fig. S42** Gibbs free energy diagram for ORR of FeMnN_6_ at different U


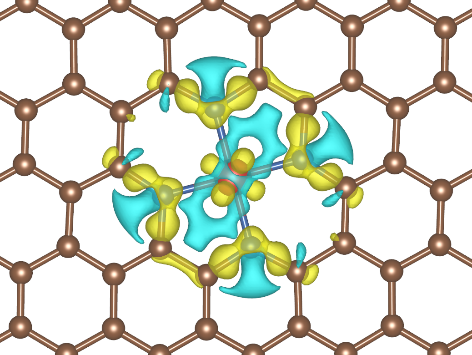


**Fig. S43** Charge density of FeN_4_ (top view)


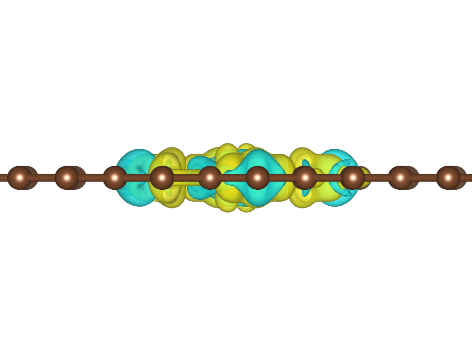


**Fig. S44** Charge density of FeN_4_ (side view)


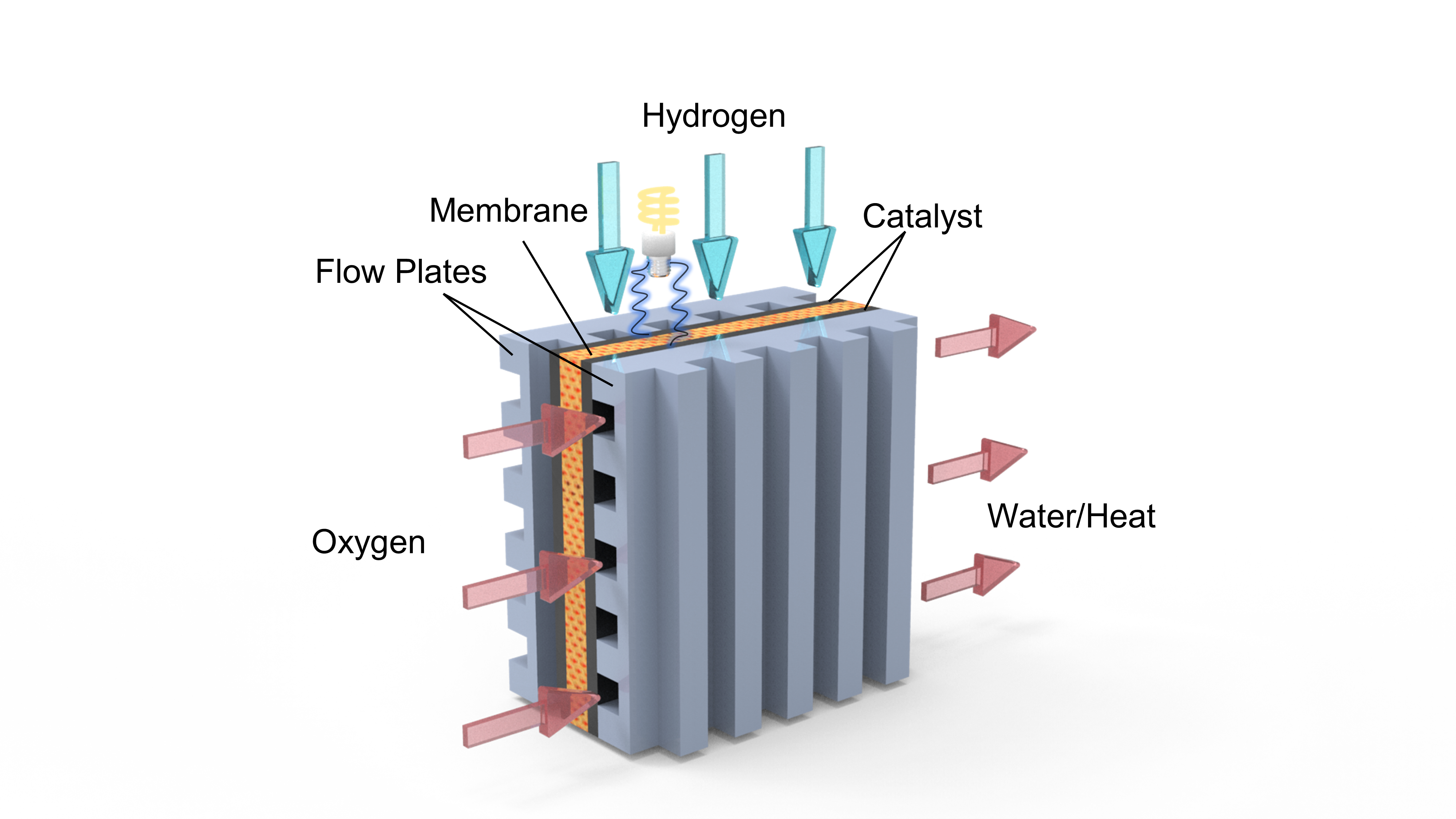


**Fig. S45** Schematic configuration of a fuel cell


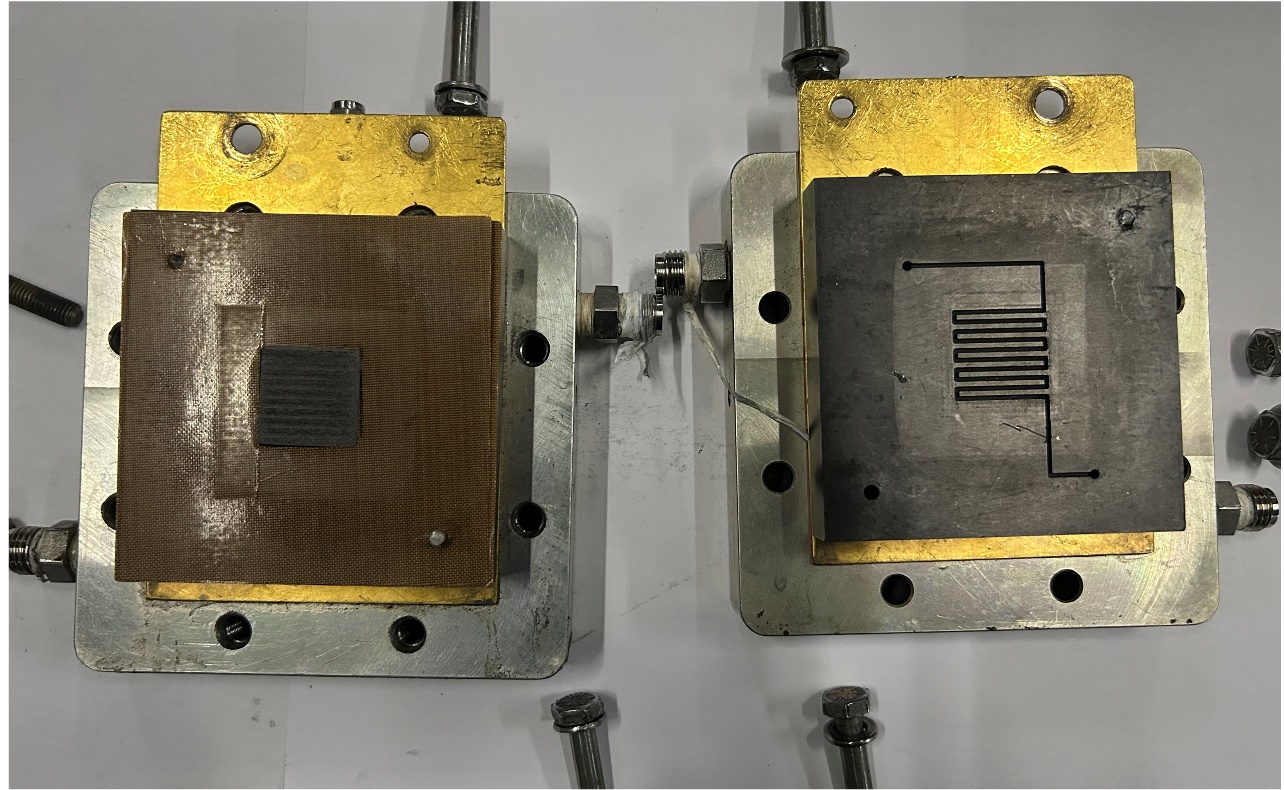


**Fig. S46** MEA of fuel cell


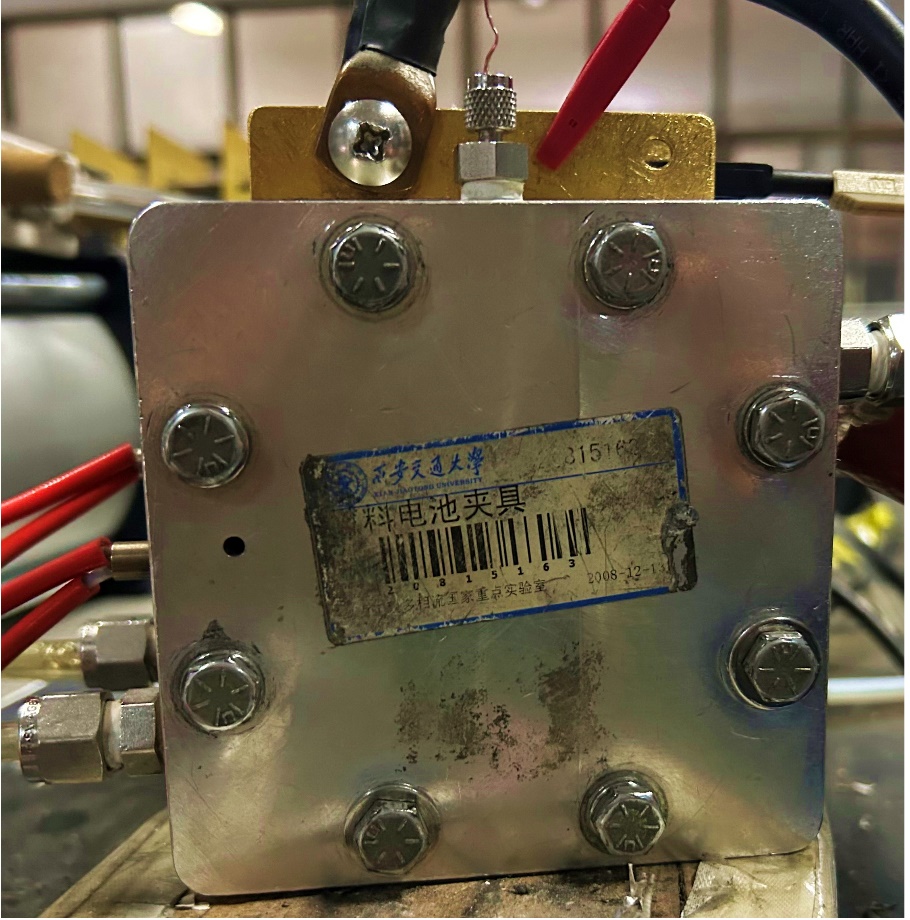


**Fig. S47** Fell cell test


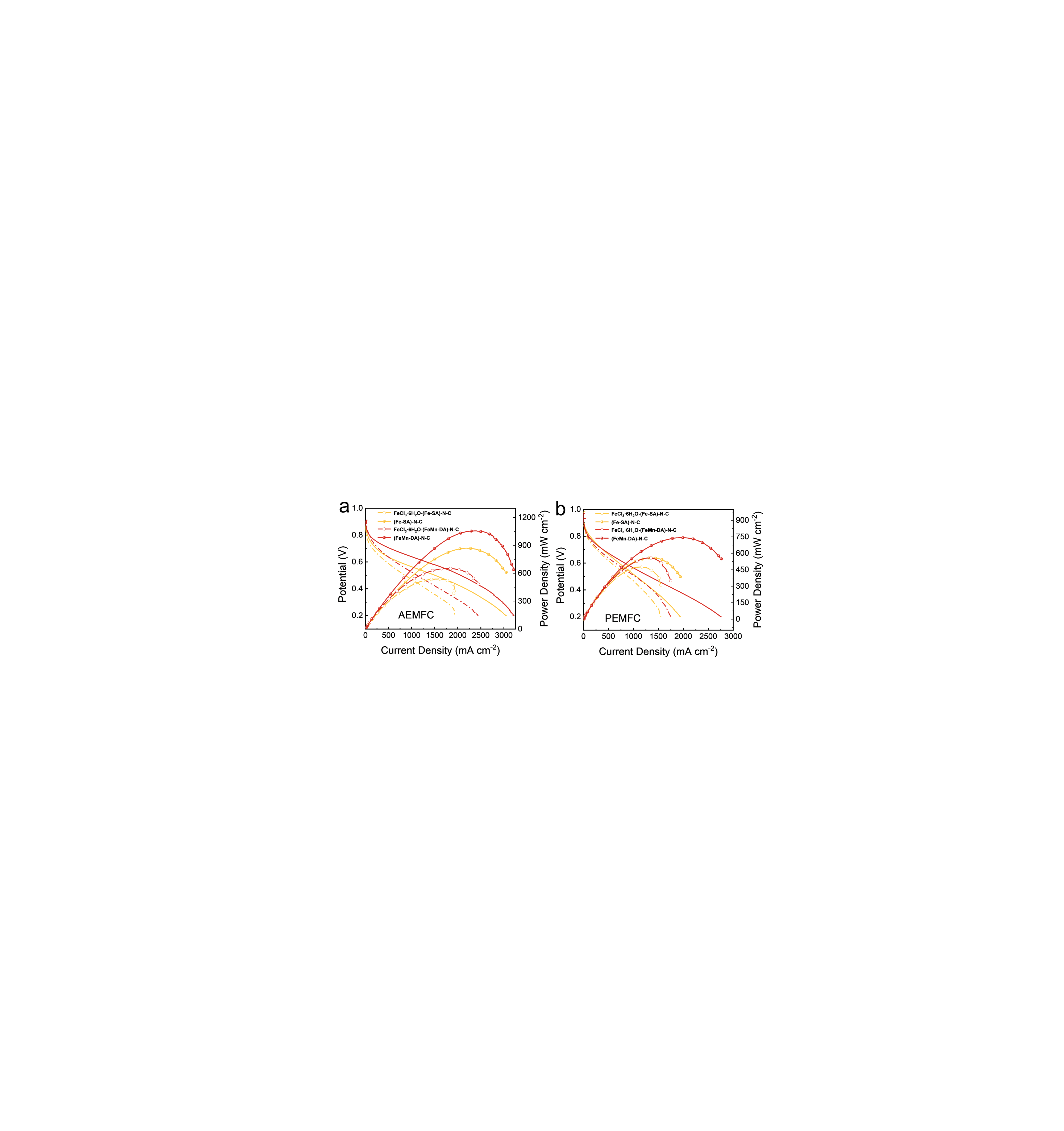


**Fig. S48** Polarization and power density curves of the different Fe source in **a** AEMFC and **b** PEMFC. The test conditions are the same as described in **Fig. 6**

**Table S1** Elemental compositions of Fe and Mn contents before and after ADT in alkaline and acid measured by ICP-MS

| Catalysts | Before (wt%) | | After ADT in 0.1 M  KOH (wt%） | | After ADT in 0.1 M HClO_4_ (wt%） | |
| --- | --- | --- | --- | --- | --- | --- |
|  | Fe | Mn | Fe | Mn | Fe | Mn |
| (Fe-SA)-N-C | 3.03 | / | 2.52 | / | 2.04 | / |
| (FeMn-DA)-N-C | 3.27 | 1.48 | 2.95 | 1.32 | 2.79 | 1.24 |

**Table S2** Structural parameters extracted from the EXAFS fitting

| Sample | Path | N | R (Å) | σ^2^ (Å^2^) | R-factor |
| --- | --- | --- | --- | --- | --- |
| Fe in (FeMn-DA)-N-C | Fe-N | 4.06±0.46 | 2.03 | 0.00419 | 0.00158 |
|  | Fe-Mn | 0.7±1.38 | 2.50 | 0.01305 |  |
| Mn in (FeMn-DA)-N-C | Mn-N | 4.59±0.23 | 2.05 | 0.0014 | 0.00143 |
|  | Mn-Fe | 1.09±0.56 | 2.52 | 0.013 |  |

**Table S3** Comparison of ORR performance of this work with different catalyst in O_2_-saturated 0.1 M KOH at 1600 rpm

| Catalyst | *E_onset_* (V) | E_1/2_ (V) | J_k_ at 0.9 V (mA cm^-2^) | K_tafel_ (mV dec^-1^) |
| --- | --- | --- | --- | --- |
| N-C | 0.86 | 0.76 | 0.08 | 63.13 |
| (Mn-SA)-N-C | 0.9 | 0.81 | 0.16 | 81.78 |
| (Fe-SA)-N-C | 0.99 | 0.9 | 5.13 | 59.79 |
| (FeMn-DA)-N-C | 1.02 | 0.92 | 15.42 | 47.16 |
| Pt/C | 0.98 | 0.85 | 1.86 | 74.02 |

**Table S4** Comparison of ORR performance of this work with different catalyst in O_2_-saturated 0.1 M HClO_4_ at 1600 rpm

| Catalyst | E_onset_ (V) | E_1/2_ (V) | J_k_ at 0.75 V (mA cm^-2^) | K_tafel_ (mV dec^-1^) |
| --- | --- | --- | --- | --- |
| N-C | 0.79 | 0.67 | 0.29 | 68.1 |
| (Mn-SA)-N-C | 0.82 | 0.72 | 1.22 | 66.29 |
| (Fe-SA)-N-C | 0.9 | 0.78 | 6.91 | 61.25 |
| (FeMn-DA)-N-C | 0.94 | 0.82 | 12.74 | 45.63 |
| Pt/C | 0.96 | 0.85 | 1.86 | 65.9 |

**Table S5** Comparison of ORR performance of (FeMn-DA)-N-C with those reported in literature literature in alkaline

| Catalyst | E_onset_  (V) | E_1/2_  (V) | J_k_  (mA cm^-2^) | K_tafel_  (mV dec^-1^) | Decay by Chronoamperometric | References |
| --- | --- | --- | --- | --- | --- | --- |
| (FeMn-DA)-N-C | 1.02 | 0.92 | 15.42 at 0.9 V | 47.16 | **10.6 %/100 h** | **this work** |
| FeN_4_–Ti_3_C_2_S_x_ | 1.01 | 0.89 | / | 48 | 16 %/24h | [S12] |
| Co-N_x_/C-MnO | 0.93 | 0.87 | / | 79.5 | 20.95 %/100 h | [S13] |
| Pt@CoN_4_-G | 1.001 | 0.893 | 17.89 at 0.85 V | / | 5 %/200 h | [S14] |
| Fe-N_4_SP/NPS-HC | / | 0.912 | 60 at 0.85V | 39.18 | 5 %/70 h | [S15] |
| FeN_4_-700/900 | / | 0.904 | [28.4 at 0.8](mailto:28.4@0.8) V | 71 | ≈26 %/11.1 h | [S16] |
| Fe_3_Co_7_-NC | 1.02 | 0.893 | / | 56 | / | [S17] |
| Fe/Fe_x_C@Fe-N-C-900 | 1.01 | 0.91 | [19.84 at 0.85](mailto:19.84@0.85) V | 44.8 | ≈10%/13.9 h | [S18] |
| Co@C-CoNC | 0.988 | 0.906 | 29.67 [at 0.85](mailto:V@0.85) V | 65 | / | [S19] |
| Fe@C-FeNC | 1.025 | 0.917 | [30.96 at 0.85](mailto:30.96@0.85) V | 64 | ≈18 %/2.8 h | [S19] |
| Cu@C-CuNC | 0.976 | 0.829 | [3.03 at 0.85](mailto:3.03@0.85) V | 71 | / | [S19] |
| Co-SAC/NC | 1.019 | 0.884 | 3.48 at 0.9 V | / | 10 %/~8.3 h | [S20] |
| Fe/Zn-N-C | 0.985 | 0.906 | / | 47 | / | [S21] |
| Fe-1 | / | 0.882 | [30.5 at 0.82](mailto:30.5@0.82) V | 82 | 4.6 %/10 h | [S22] |
| FeNSC-2Fe | 1.007 | 0.913 | [15.7 at 0.85](mailto:15.7@0.85) V | 59 | ≈0.8 %/11.1 h | [S23] |
| Fe@Fe-N-C | / | 0.916 | 60.9 | 66.3 | 1.5 %/16 h | [S24] |
| Fe-N-C | / | 0.905 | 23.9 | 64.3 | 5.3 %/16 h | [S24] |
| FeCo-NSC | ~0.98 | 0.86 | / | 75 | 13.9 %/~11.1 h | [S25] |
| Fe-N-C-900 | 0.99 | 0.927 | 11 at 0.9 V | 71.8 | 8.7 %/~14.4 h | [S26] |
| Fe-N/P-C-700 | 0.941 | 0.867 | 7.63 at 0.85 V | 68.6 | 5 %/10 h | [S27] |
| Cu_SA_/Cu_CT_@NPC | 1 | 0.88 | / | 69 | ≈21.1 %/13.9 h | [S28] |
| Fe_SA_/NMCs-800 | 0.98 | 0.8 | / | 104 | / | [S29] |
| Se@NC-1000 | 0.95 | 0.86 | 5.53 at 0.85V | 52 | 4 %/10 h | [S30] |

**Table S6** Comparison of ORR performances of (FeMn-DA)-N-C with those reported in literature in acid

| Catalyst | E_onset_  (V) | E_1/2_  (V) | J_k_  (mA cm^-2^) | K_tafel_  (mV dec^-1^) | Decay by Chronoamperometric | Reference |
| --- | --- | --- | --- | --- | --- | --- |
| (FeMn-DA)-N-C | 0.94 | 0.82 | 12.74 at 0.75 V | 45.63 | **14 %/50 h** | **this work** |
| Cox/C-MnO | 0.8 | 0.66 | / | 76.3 | 5.6 %/100 h | [S13] |
| Fe-N_4_SP/NPS-HC | / | 0.814 | 46.7 at 0.7 V | 49.72 | 3 %/70 h | [S15] |
| Fe/Fe_x_C@Fe-N-C900 | 0.96 | 0.81 | 95.81 at 0.75 V | 81.9 | ≈14 %/13.9 h | [S18] |
| Fe/Zn-N-C | / | 0.808 | / | 79 | 9.6 %/20 h | [S21] |
| Fe@Fe-N-C | 0.952 | 0.829 | [11.1 at 0.8](mailto:11.1@0.8) V | 75.5 | 11.2 %/10 h | [S24] |
| Fe-N-C | 0.931 | 0.807 | [5.07 at 0.8](mailto:5.07@0.8) V | 76.3 | 20.7 %/10 h | [S24] |
| OAC | 0.86 | 0.71 | [6.57 at 0.7](mailto:6.57@0.7) V | 61 | ≈12 %/5.6 h | [S31] |
| Fe_SA/_NMC-800 | 0.93 | 0.71 | 41 at 0.6 V | 68 | 15 %/20 h | [S29] |
| Cu_SA_/Cu_CT_@NPC | 0.87 | 0.80 | / | 61 | 37.5%/13.9 h | [S28] |
| Fe_1_/d-CN | 0.91 | 0.79 | 25.6 at 0.75 V | 64 | / | [S32] |
| Co-Se-N-C-2 | 0.84 | 0.764 | / | 54 | ≈5 %/3.9 h | [S33] |
| Fe,Mn/N-C | 0.97 | 0.804 | 7.75 | 79 | ≈7.4 %/11.1 h | [S34] |
| CoFe-NC | 0.938 | 0.804 | / | 76.8 | / | [S35] |
| Fe-NX/C-NC 1100 | 0.94 | 0.811 | / | 48 | 19.1 %/50 h | [S36] |
| Co_2_/Fe-N@CHC | 0.96 | 0.812 | 8.36 at 0.8 V | 73 | / | [S37] |
| Co-SAs/N-C/rGO | 0.89 | 0.77 | 7.09 at 0.75 V | 65 | 26.44 %/20 h | [S38] |
| Co-N/C+NP+NG | 0.96 | 0.815 | / | 75 | / | [S39] |
| S-POP | 0.82 | 0.71 | / | 89 | ≈10 %/5.56 h | [S40] |
| Fe-N-C/N-OMC | 0.91 | 0.73 | 0.21 | 76 | / | [S41] |
| ZnCo-NC-II | 0.941 | 0.786 | / | 79.67 | / | [S42] |

**Table S7** O_2_ adsorption energy of different configurations. (Unit: eV)

| configuration | surface | O_2_ | $G_{\mathrm{total}}$ | $G_{\mathrm{ads}}$ |
| --- | --- | --- | --- | --- |
| FeMn | -644.100 | -9.942 | -655.177 | -1.135 |
| Fe |  |  | -654.886 | -0.844 |
| Mn |  |  | -655.095 | -1.053 |

**Table S8** The reaction free energy of reverse ORCM initiated by the O_Fe_ site. (Unit: eV)

| structure | O_2_ | *OH+*O | *O | *OH | surface |
| --- | --- | --- | --- | --- | --- |
| DFT energy | -9.942 | -660.290 | -650.286 | -654.518 | -644.100 |
| ΔG |  | -2.838 | -0.836 | -0.821 | **-0.422** |

**Table S9** The adsorption free energy of intermediates. (Unit: eV)

| ΔG | *O | *OH |
| --- | --- | --- |
| AEM-Fe | 1.921 | 0.890 |
| ORCM-O_Fe_ | 1.242 | 0.422 |

**Table S10** The reaction free energy of reverse AEM on the Mn site. (Unit: eV)

| structure | O_2_ | *OOH | *O | *OH | surface |
| --- | --- | --- | --- | --- | --- |
| DFT energy | -9.942 | -658.937 | -650.286 | -654.518 | -644.100 |
| ΔG |  | -1.485 | -2.189 | -0.821 | **-0.422** |

**Table S11** The reaction free energy of reverse ORCM initiated by the O_Mn_ site. (Unit: eV)

| structure | O_2_ | *OH+*O | *O | *OH | surface |
| --- | --- | --- | --- | --- | --- |
| DFT energy | -9.942 | -660.026 | -649.608 | -654.049 | -644.100 |
| ΔG |  | -2.574 | **-0.421** | -1.031 | -0.890 |

**Table S12** Valence state of atoms in FeMnN_6_ and FeN_4_ by bader charge analysis

| structure | Fe | Mn | N(average) |  |
| --- | --- | --- | --- | --- |
| FeMnN_6_ | +1.180 | +1.231 | -0.995 |  |
| FeN_4_ | +0.964 | - | -0.812 |  |

**Table S13** Free energy of reactant and product molecules

|  | Temperature/K | Partial pressure/atm | $E_{0}$/eV | $\Delta G$/eV | $G$/eV |
| --- | --- | --- | --- | --- | --- |
| H_2_O(l) | 298.15 | 0.035 | -14.248 | -0.002 | -14.250 |
| H_2_(g) | 298.15 | 1 | -6.777 | -0.045 | -6.821 |
| O_2_(g) | 298.15 | 1 | - | - | -9.942 |

**Table S14** Free energy of bare and intermediates adsorbed surfaces

| surfaces | energy | FeN_4_ | FeMnN_6_ |
| --- | --- | --- | --- |
| bare | *E*/eV | -655.436 | -644.100 |
| *OOH | *E*/eV | -670.538 | -658.941 |
|  | Δ*G*/eV | 0.350 | 0.387 |
|  | *G*/eV | -670.188 | -658.554 |
| *O | *E*/eV | -661.547 | -649.656 |
|  | Δ*G*/eV | 0.052 | 0.048 |
|  | *G*/eV | -661.495 | -649.608 |
| *OH | *E*/eV | -665.983 | -654.343 |
|  | Δ*G*/eV | 0.304 | 0.294 |
|  | *G*/eV | -665.679 | -654.049 |

**Table S15** Adsorption free energy of intermediates and free energy change of each elementary step. (U=0 V)

| Δ*G*/eV | *OOH | *O | *OH | Step1 | Step2 | Step3 | Step4 |
| --- | --- | --- | --- | --- | --- | --- | --- |
| FeN_4_ | 3.516 | 1.370 | 0.596 | -1.400 | -2.146 | -0.774 | -0.596 |
| FeMnN_6_ | 3.814 | 1.921 | 0.890 | -1.102 | -1.893 | -1.031 | -0.890 |

**Table S16** The chemical potential of elements in their most stable state. (Unit: eV)

| element | C(graphite) | N(nitrogen) | Fe(bulk) | Mn(bulk) |
| --- | --- | --- | --- | --- |
| $\mu$ | -9.368 | -8.335 | -8.606 | -9.376 |

**Table S17** Ohmic (Rs) and charge-transfer (Rct) impedance of AEMFC using (Fe-SA)-N-C and (FeMn-DA)-N-C as the cathode catalysts at 0.6 V

| AEMFC | Rs (*Ω cm^2^*) | Rct (*Ω cm^2^*) |
| --- | --- | --- |
| (Fe-SA)-N-C | 0.027283 | 0.10361 |
| (FeMn-DA)-N-C | 0.029691 | 0.078566 |

**Table S18** Ohmic and charge-transfer impedance of PEMFC using (Fe-SA)-N-C and (FeMn-DA)-N-C as the cathode catalysts at 0.6 V

| PEMFC | Rs (*Ω cm^2^*) | Rct (*Ω cm^2^*) |
| --- | --- | --- |
| (Fe-SA)-N-C | 0.06809 | 0.230557 |
| (FeMn-DA)-N-C | 0.06983 | 0.16264 |

**Table S19** Comparison of performance of AEMFCs using non-noble metal cathode catalysts

| Cathode catalyst | Temperature  (℃) | Back pressure (bar) | Membrane | Peak power density  (w cm^-2^) | Reference |
| --- | --- | --- | --- | --- | --- |
| (FeMn-DA)-N-C | 80 | 2 | PAP-TP-85 | **1.06** | **this work** |
| α-Mn_2_O_3_/FeNC | 60 | 0 | ETFE | 1.04 | [S43] |
| Fe-N-C | 80 | / | VTLCPET | 0.76 | [S44] |
| Ce/Fe-NCNW | 80 | 2 | Poly (terphenylene) | 0.5 | [S45] |
| Fe/N/C | 60 | 1 | Quaternary ammonia polysulfone | 0.475 | [S46] |
| N-C | 60 | 0 | Poly (ethylene- cotetrafluoroethylene) | 0.703 | [S47] |
| CoMn_2_O_4_ | 80 | 1 | QAPTT | 0.97 | [S48] |
| NiCo-N-C | 50 | 0 | FAA-3-50  FuMa-Tech | 0.065 | [S49] |
| Co-N-C | 60 | 0 | FAA-3-50  FuMA-Tech | 0.051 | [S50] |
| TiCDC/CNT (1:3)/FePc | 60 | 2 | HMT-PMBI | 0.182 | [S51] |
| FeS-iNC_50a | 60 | / | FAA-3-50  FuMa-Tech | 0.208 | [S41] |
| Fe-N-C | 60 | 2 | HMT-PMBI | 0.22 | [S52] |
| CoFe-N-OMC/CNT | 65 | 2 | HMT-PMBI | 0.336 | [S53] |
| Fe-N-C-1000 | 60 | 1 | FAA-3-50  FuMa-Tech | 0.149 | [S38] |
| ZnNC | 80 | 2 | PAP-TP-8  5 | 0.83 | [S54] |

**Table S20** Comparison of performance of the H_2_/O_2_ PEMFCs using non-noble metal cathode catalysts

| Cathode catalyst | Temperature (℃) | Back pressure (bar) | H_2_/O_2_ Flow  rate (sccm) | Peak power density (w cm^-2^) | Reference |
| --- | --- | --- | --- | --- | --- |
| (FeMn-DA)-N-C | 80 | 2 | 200 /500 | **0.746** | **this work** |
| Fe-ZIF/CN-UC | 70 | 2 | 200/500 | 0.484 | [S9] |
| Fe-N_X_/C-NC  1100 | 70 | 1 | 300/400 | 0.656 | [S36] |
| Ce SAS/HPNC | 80 | 2 | / | 0.525 | [S55] |
| Fe-N-C/MA | 80 | 1 | 200/200 | 0.47 | [S56] |
| 3D Fe-N-C | 70 | 1 | 300/400 | 0.6 | [S57] |
| FeNC-1:15 | 80 | 1 | 200/300 | 0.63 | [S58] |
| Fe (7.5) NC_950 | 80 | 2 | 200/200 | 0.353 | [S59] |
| Spa-S-Fe,Co/NC | 80 | 2 | 300/300 | 0.663 | [S60] |
| 0.14Co0.01Fe-CB | 80 | 1 | 400/800 | 0.465 | [S61] |
| Ce SAS/HPNC | 80 | 2 | / | 0.525 | [S62] |
| PCN-A@Fe SA | 70 | 2 | / | 0.514 | [S63] |
| SA-Fe-NHPC | 80 | / | 200/1000 | 0.423 | [S64] |
| FeMn(mlm)-N-C | 80 | 1 | / | 0.61 | [S65] |

**Supplementary References**

1. P. E. Blöchl. Projector augmented-wave method. Phys. Rev. B **50**(24), 17953-17979 (1994). <https://doi.org/10.1103/PhysRevB.50.17953>
2. G. Kresse, D. Joubert. From ultrasoft pseudopotentials to the projector augmented-wave method. Phys. Rev. B **59**(3), 1758-1775 (1999). <https://doi.org/10.1103/PhysRevB.59.1758>
3. G. Kresse, J. Furthmüller. Efficiency of ab-initio total energy calculations for metals and semiconductors using a plane-wave basis set. Comput. Mater. Sci. **6**(1), 15-50 (1996). <https://doi.org/10.1016/0927-0256(96)00008-0>
4. G. Kresse, J. Furthmüller. Efficient iterative schemes for ab initio total-energy calculations using a plane-wave basis set. Phys. Rev. B **54**(16), 11169-11186 (1996). <https://doi.org/10.1103/PhysRevB.54.11169>
5. J. P. Perdew, K. Burke, M. Ernzerhof. Generalized gradient approximation made simple. Phys. Rev. Lett. **77**(18), 3865-3868 (1996). <https://doi.org/10.1103/PhysRevLett.77.3865>
6. S. Grimme, J. Antony, S. Ehrlich, H. Krieg. A consistent and accurate ab initio parametrization of density functional dispersion correction (dft-d) for the 94 elements h-pu. J. Chem. Phys. **132**(15), 154104 (2010). <https://doi.org/10.1063/1.3382344>
7. S. Grimme, S. Ehrlich, L. Goerigk. Effect of the damping function in dispersion corrected density functional theory. J. Comput. Chem. **32**(7), 1456-1465 (2011). <https://doi.org/10.1002/jcc.21759>
8. V. Wang, N. Xu, J.-C. Liu, G. Tang, W.-T. Geng. Vaspkit: A user-friendly interface facilitating high-throughput computing and analysis using vasp code. Comput. Phys. Commun. **267**, 108033 (2021). <https://doi.org/10.1016/j.cpc.2021.108033>
9. L. Zhang, L. Li, Z. Gao, L. Guo, M. Li et al., Porous hierarchical iron/nitrogen co‐doped carbon etched by g‐c_3_n_4_ pyrolysis as efficient non‐noble metal catalysts for pem fuel cells. ChemElectroChem **9**(6), e202101681 (2022). <https://doi.org/10.1002/celc.202101681>
10. L. Li, L. Zhang, T. Zhai, S. Yang, W. Wang et al., Structurally ordered ptni intermetallic nanoparticles as efficient and stable cathode catalysts for proton exchange membrane fuel cells. Chem-Eur J. **29**(27), e202300099 (2023). <https://doi.org/10.1002/chem.202300099>
11. Y.-H. Wang, J.-B. Le, W.-Q. Li, J. Wei, P. M. Radjenovic et al., In situ spectroscopic insight into the origin of the enhanced performance of bimetallic nanocatalysts towards the oxygen reduction reaction (ORR). Angew. Chem. Int. Ed. **58**(45), 16062-16066 (2019). https://doi.org/10.1002/anie.201908907
12. K. Miao, W. Jiang, Z. Chen, Y. Luo, D. Xiang et al., Hollow-structured and polyhedron-shaped high entropy oxide toward highly active and robust oxygen evolution reaction in a full pH range. Adv. Mater. **36**(8), 2308490 (2024). <https://doi.org/10.1002/adma.202308490>
13. Z. Qiu, Y. Li, Y. Gao, Z. Meng, Y. Sun et al., 2d mof-assisted pyrolysis-displacement-alloying synthesis of high-entropy alloy nanoparticles library for efficient electrocatalytic hydrogen oxidation. Angew. Chem. Int. Ed. **62**(33), e202306881 (2023). <https://doi.org/10.1002/anie.202306881>
14. M. Zhang, H. Li, J. Chen, F.-X. Ma, L. Zhen et al., A low-cost, durable bifunctional electrocatalyst containing atomic co and pt species for flow alkali-al/acid hybrid fuel cell and zn–air battery. Adv. Funct. Mater. **33**(47), 2303189 (2023). <https://doi.org/10.1002/adfm.202303189>
15. J. Liu, W. Chen, S. Yuan, T. Liu, Q. Wang. High-coordination fe–n_4_sp single-atom catalysts via the multi-shell synergistic effect for the enhanced oxygen reduction reaction of rechargeable zn–air battery cathodes. Energy Environ. Sci. **17**(1), 249-259 (2024). <https://doi.org/10.1039/D3EE03183G>
16. B. Zhao, D. Xue, P. Yuan, W. Yan, J. Zhang et al., Optimizing electrocatalytic oxygen reduction by adjacent c-o-c structure-driven charge separation on fen_4_ active sites. Appl. Catal. B **324**, 122251 (2023). <https://doi.org/10.1016/j.apcatb.2022.122251>
17. T. Gu, D. Zhang, Y. Yang, C. Peng, D. Xue et al., Dual-sites coordination engineering of single atom catalysts for full-temperature adaptive flexible ultralong-life solid-state zn−air batteries. Adv. Funct. Mater. **33**(8), 2212299 (2023). <https://doi.org/10.1002/adfm.202212299>
18. M. Chen, F. Kong, H. Yao, Y. Chen, G. Meng et al., Dual metal-organic frameworks-derived fe-atomic sites bounded to fine fe/fe_x_c nanoparticles for enhanced oxygen electroreduction. Chem. Eng. J. **453**, 139820 (2023). <https://doi.org/10.1016/j.cej.2022.139820>
19. S. Chandrasekaran, R. Hu, L. Yao, L. Sui, Y. Liu et al., Mutual self-regulation of d-electrons of single atoms and adjacent nanoparticles for bifunctional oxygen electrocatalysis and rechargeable zinc-air batteries. Nano-Micro Lett. **15**(1), 48 (2023). <https://doi.org/10.1007/s40820-023-01022-8>
20. P. Rao, J. M. Luo, D. X. Wu, J. Li, Q. Chen et al., Isolated co atoms anchored on defective nitrogen-doped carbon graphene as efficient oxygen reduction reaction electrocatalysts. Energy Environ. Mater. **6**, (2023). <https://doi.org/10.1002/eem2.12371>
21. H. Li, S. Di, P. Niu, S. Wang, J. Wang et al., A durable half-metallic diatomic catalyst for efficient oxygen reduction. Energy Environ. Sci. **15**(4), 1601-1610 (2022). <https://doi.org/10.1039/D1EE03194E>
22. J. Sheng, S. Sun, G. Jia, S. Zhu, Y. Li. Doping effect on mesoporous carbon-supported single-site bifunctional catalyst for zinc–air batteries. ACS Nano **16**(10), 15994-16002 (2022). <https://doi.org/10.1021/acsnano.2c03565>
23. C. Shao, L. Wu, Y. Wang, K. Qu, H. Chu et al., Engineering asymmetric fe coordination centers with hydroxyl adsorption for efficient and durable oxygen reduction catalysis. Appl. Catal. B **316**, 121607 (2022). <https://doi.org/10.1016/j.apcatb.2022.121607>
24. L. Li, Y. Wen, G. Han, F. Kong, L. Du et al., Architecting fen_x_ on high graphitization carbon for high-performance oxygen reduction by regulating d-band center. Small **19**(22), 2300758 (2023). <https://doi.org/10.1002/smll.202300758>
25. Y. Y. Wu, C. C. Ye, L. Yu, Y. F. Liu, J. F. Huang et al., Soft template-directed interlayer confinement synthesis of a fe-co dual single-atom catalyst for zn-air batteries. Energy Stor. Mater. **45**, 805-813 (2022). <https://doi.org/10.1016/j.ensm.2021.12.029>
26. C. Z. Zhu, Q. R. Shi, B. Z. Xu, S. F. Fu, G. Wan et al., Hierarchically porous m-n-c (m = co and fe) single-atom electrocatalysts with robust mn_x_ active moieties enable enhanced orr performance. Adv. Energy Mater. **8**(29), 1801956 (2018). <https://doi.org/10.1002/aenm.201801956>
27. H. Liu, L. Jiang, Y. Wang, X. Wang, J. Khan et al., Boosting oxygen reduction with coexistence of single-atomic Fe and Cu sites decorated nitrogen-doped porous carbon. Chem. Eng. J. **452**, 38938 (2023). <https://doi.org/10.1016/j.cej.2022.138938>
28. Z. Yang, K. Jiang, G. Tong, C. Ke, H. Wu et al., Copper-involved highly efficient oxygen reduction reaction in both alkaline and acidic media. Chem. Eng. J. **437**, 135377 (2022). <https://doi.org/10.1016/j.cej.2022.135377>
29. H. Xie, B. Du, X. Huang, D. Zeng, H. Meng et al., High density single fe atoms on mesoporous n-doped carbons: Noble metal-free electrocatalysts for oxygen reduction reaction in acidic and alkaline media. Small **19**(32), 2303214 (2023). <https://doi.org/10.1002/smll.202303214>
30. H. Hu, J. J. Wang, B. F. Cui, X. R. Zheng, J. G. Lin et al., Atomically dispersed selenium sites on nitrogen-doped carbon for efficient electrocatalytic oxygen reduction. Angew. Chem. Int. Ed. **61**(3), (2022). <https://doi.org/10.1002/anie.202114441>
31. L. Deng, L. Qiu, R. Hu, L. Yao, Z. Zheng et al., Restricted diffusion preparation of fully-exposed fe single-atom catalyst on carbon nanospheres for efficient oxygen reduction reaction. Appl. Catal. B **305**, 121058 (2022). <https://doi.org/10.1016/j.apcatb.2021.121058>
32. M. Zhao, H. Liu, H. Zhang, W. Chen, H. Sun et al., A ph-universal orr catalyst with single-atom iron sites derived from a double-layer mof for superior flexible quasi-solid-state rechargeable zn–air batteries. Energy Environ. Sci. **14**(12), 6455-6463 (2021). <https://doi.org/10.1039/d1ee01602d>
33. J. Lian, J. Zhao, X. Wang, Q. Bai. Zif-derived porous carbon supported cobalt and selenium dual sites enhanced oxygen reduction reaction. Carbon **213,** 118257 (2023). <https://doi.org/10.1016/j.carbon.2023.118257>
34. G. Yang, J. Zhu, P. Yuan, Y. Hu, G. Qu et al., Regulating fe-spin state by atomically dispersed mn-n in fe-n-c catalysts with high oxygen reduction activity. Nat. Commun. **12**(1), 1734 (2021). <https://doi.org/10.1038/s41467-021-21919-5>
35. K. Wang, J. P. Liu, Z. H. Tang, L. G. Li, Z. Wang et al., Establishing structure/property relationships in atomically dispersed co-fe dual site m-n_x_ catalysts on microporous carbon for the oxygen reduction reaction. J. Mater. Chem. A **9**(22), 13044-13055 (2021). <https://doi.org/10.1039/d1ta02925h>
36. X. M. Qu, Y. Han, Y. H. Chen, J. X. Lin, G. Li et al., Stepwise pyrolysis treatment as an efficient strategy to enhance the stability performance of fe-n_x_/c electrocatalyst towards oxygen reduction reaction and proton exchange membrane fuel cell. Appl. Catal. B **295**, 120311 (2021). <https://doi.org/10.1016/j.apcatb.2021.120311>
37. Z. Wang, X. Jin, C. Zhu, Y. Liu, H. Tan et al., Atomically dispersed Co_2_–N_6_ and Fe–N_4_ costructures boost oxygen reduction reaction in both alkaline and acidic media. Adv. Mater. **33**(49), 2104718 (2021). <https://doi.org/10.1002/adma.202104718>
38. W. da Silva Freitas, A. D'Epifanio, C. L. Vecchio, I. Gatto, V. Baglio et al., Tailoring MOF structure via iron decoration to enhance orr in alkaline polymer electrolyte membrane fuel cells. Chem. Eng. J. **465**, 142987 (2023). <https://doi.org/10.1016/j.cej.2023.142987>
39. X. Zhang, X. Xu, S. Yao, C. Hao, C. Pan et al., Boosting electrocatalytic activity of single atom catalysts supported on nitrogen-doped carbon through n coordination environment engineering. Small **18**(10), 2105329 (2022). <https://doi.org/10.1002/smll.202105329>
40. W. Yan, S. Cao, Z. Xiao, F. Dai, T. Xing et al., Novel heteroatom sulfur porphyrin organic polymer as a metal-free electrocatalyst for acidic oxygen reduction reaction. Electrochim. Acta **377**, 138107 (2021). <https://doi.org/10.1016/j.electacta.2021.138107>
41. H. S. Kim, C. H. Lee, J.-H. Jang, M. S. Kang, H. Jin et al., Single-atom oxygen reduction reaction electrocatalysts of fe, si, and n co-doped carbon with 3d interconnected mesoporosity. J. Mater. Chem. A **9**(7), 4297-4309 (2021). <https://doi.org/10.1039/D0TA11208A>
42. K. Song, Y. Feng, X. Zhou, T. Qin, X. Zou et al., Exploiting the trade-offs of electron transfer in mof-derived single zn/co atomic couples for performance-enhanced zinc-air battery. Appl. Catal. B **316**, 121591 (2022). <https://doi.org/10.1016/j.apcatb.2022.121591>
43. P. G. Santori, F. D. Speck, S. Cherevko, H. A. Firouzjaie, X. Peng et al., High performance fenc and mn-oxide/fenc layers for aemfc cathodes. J. Electrochem. Soc. **167**(13), 134505 (2020). <https://doi.org/10.1149/1945-7111/abb7e0>
44. Y. J. Sa, J. Woo, M. G. Kim, T.-Y. Kim, S. H. Joo. A general approach to preferential formation of active fe-n_x_ sites in fe-n/c electrocatalysts for high-performance polymer electrolyte fuel cells. Electrochemical Society Meeting Abstracts 230. 38), 2683-2683 (2016)
45. J.-C. Li, S. Maurya, Y. S. Kim, T. Li, L. Wang et al., Stabilizing single-atom iron electrocatalysts for oxygen reduction via ceria confining and trapping. ACS Catal. **10**(4), 2452-2458 (2020). <https://doi.org/10.1021/acscatal.9b04621>
46. H. Ren, Y. Wang, Y. Yang, X. Tang, Y. Peng et al., Fe/N/C nanotubes with atomic Fe sites: A highly active cathode catalyst for alkaline polymer electrolyte fuel cells. ACS Catal. **7**(10), 6485-6492 (2017). <https://doi.org/10.1021/acscatal.7b02340>
47. Y. Lu, L. Wang, K. Preuß, M. Qiao, M.-M. Titirici et al., Halloysite-derived nitrogen doped carbon electrocatalysts for anion exchange membrane fuel cells. J. Power Sources **372**, 82-90 (2017). <https://doi.org/10.1016/j.jpowsour.2017.10.037>
48. Y. Yang, H. Peng, Y. Xiong, Q. Li, J. Lu et al., High-loading composition-tolerant co–mn spinel oxides with performance beyond 1 w/cm^2^ in alkaline polymer electrolyte fuel cells. ACS Energy Lett. **4**(6), 1251-1257 (2019). <https://doi.org/10.1021/acsenergylett.9b00597>
49. S. Hanif, N. Iqbal, X. Shi, T. Noor, G. Ali et al., Nico–n-doped carbon nanotubes based cathode catalyst for alkaline membrane fuel cell. Renew Energ. **154**(508-516 (2020). <https://doi.org/10.1016/j.renene.2020.03.060>
50. P. Teppor, R. Jäger, M. Paalo, A. Adamson, M. Härmas et al., Peat as a carbon source for non-platinum group metal oxygen electrocatalysts and aemfc cathodes. Int. J. Hydrogen Energy **47**(38), 16908-16920 (2022). <https://doi.org/10.1016/j.ijhydene.2022.03.199>
51. R. Praats, M. Käärik, A. Kikas, V. Kisand, J. Aruväli et al., Electrocatalytic oxygen reduction reaction on iron phthalocyanine-modified carbide-derived carbon/carbon nanotube composite electrocatalysts. Electrochim. Acta **334**, 135575 (2020). <https://doi.org/10.1016/j.electacta.2019.135575>
52. J. Lilloja, M. Mooste, E. Kibena-Põldsepp, A. Sarapuu, B. Zulevi et al., Mesoporous iron-nitrogen co-doped carbon material as cathode catalyst for the anion exchange membrane fuel cell. J. Power Sources **8**, 100052 (2021). <https://doi.org/10.1016/j.powera.2021.100052>
53. J. Lilloja, M. Mooste, E. Kibena-Põldsepp, A. Sarapuu, A. Kikas et al., Cobalt-, iron-and nitrogen-containing ordered mesoporous carbon-based catalysts for anion-exchange membrane fuel cell cathode. Electrochim. Acta **439**, 141676 (2023). <https://doi.org/10.1016/j.electacta.2022.141676>
54. P. Sun, Z. Qiao, S. Wang, D. Li, X. Liu et al., Atomically dispersed zn‐pyrrolic‐n_4_ cathode catalysts for hydrogen fuel cells. Angew. Chem. Int. Ed. **135**(6), e202216041 (2023). <https://doi.org/10.1002/ange.202216041>
55. M. Z. Zhu, C. Zhao, X. K. Liu, X. L. Wang, F. Y. Zhou et al., Single atomic cerium sites with a high coordination number for efficient oxygen reduction in proton-exchange membrane fuel cells. ACS Catal. **11**(7), 3923-3929 (2021). <https://doi.org/10.1021/acscatal.0c05503>
56. Y. Zhou, G. Chen, Q. Wang, D. Wang, X. Tao et al., Fe-n-c electrocatalysts with densely accessible fen_4_ sites for efficient oxygen reduction reaction. Adv. Funct. Mater. **31**(34), 2102420 (2021). <https://doi.org/10.1002/adfm.202102420>
57. R. G. Wang, Y. Y. Yang, Y. Zhao, L. J. Yang, P. F. Yin et al., Multiscale structural engineering of atomically dispersed fen_4_ electrocatalyst for proton exchange membrane fuel cells. J. Energy Chem. **58**, 629-635 (2021). <https://doi.org/10.1016/j.jechem.2020.10.036>
58. Y. L. Wu, J. L. Huang, Z. P. Lin, L. F. Li, G. F. Liang et al., Fe-n_x_ doped carbon nanotube as a high efficient cathode catalyst for proton exchange membrane fuel cell. Chem. Eng. J. **423**(130241 (2021). <https://doi.org/10.1016/j.cej.2021.130241>
59. A. Mehmood, B. Ali, M. Gong, M. Gyu Kim, J. Y. Kim et al., Development of a highly active fenc catalyst with the preferential formation of atomic iron sites for oxygen reduction in alkaline and acidic electrolytes. J. Colloid Interface Sci. **596**, 148-157 (2021). <https://doi.org/10.1016/j.jcis.2021.03.081>
60. F. Liu, L. Shi, X. Lin, B. Zhang, Y. Long et al., Fe/co dual metal catalysts modulated by s-ligands for efficient acidic oxygen reduction in pemfc. Sci. Adv. **9**(23), eadg0366 (2023). <https://doi.org/10.1126/sciadv.adg0366>
61. W. Zhu, Y. Pei, J. C. Douglin, J. Zhang, H. Zhao et al., Multi-scale study on bifunctional co/fe–n–c cathode catalyst layers with high active site density for the oxygen reduction reaction. Appl. Catal. B **299**, 120656 (2021). <https://doi.org/10.1016/j.apcatb.2021.120656>
62. M. Zhu, C. Zhao, X. Liu, X. Wang, F. Zhou et al., Single atomic cerium sites with a high coordination number for efficient oxygen reduction in proton-exchange membrane fuel cells. ACS Catal. **11**(7), 3923-3929 (2021). <https://doi.org/10.1021/acscatal.0c05503>
63. X. Shi, Z. Pu, B. Chi, M. Liu, S. Yu et al., Nitrogen and atomic fe dual-doped porous carbon nanocubes as superior electrocatalysts for acidic h_2_-o_2_ pemfc and alkaline zn-air battery. J. Energy Chem. **59**, 388-395 (2021). <https://doi.org/10.1016/j.jechem.2020.11.026>
64. G. Chen, P. Liu, Z. Liao, F. Sun, Y. He et al., Zinc‐mediated template synthesis of fe‐n‐c electrocatalysts with densely accessible fe‐n_x_ active sites for efficient oxygen reduction. Adv. Mater. **32**(8), 1907399 (2020). <https://doi.org/10.1002/adma.201907399>
65. S. Zhao, Z. Ma, Z. Wan, J. Li, X. Wang. Noble-metal-free femn-nc catalyst for efficient oxygen reduction reaction in both alkaline and acidic media. J. Colloid Interface Sci. **642**, 800-809 (2023). <https://doi.org/10.1016/j.jcis.2023.03.206>
